# Supplementary material for: Photoactuating artificial muscle from supramolecular assembly of an overcrowded alkene-derived molecular switch
Source: Nat Commun. 2025 Apr 24;16:3897. doi: 10.1038/s41467-025-58468-0 (PMC12022091; doi:10.1038/s41467-025-58468-0)
Supplement: Supplementary file 1 — Supplementary Information [file 41467_2025_58468_MOESM1_ESM.pdf]

Supplementary Information

Photoactuating Artificial Muscle from Supramolecular Assembly of an Overcrowded Alkene-derived Molecular Switch

Adrien Combe,[1] Shaoyu Chen,*[1,2] Gianni Pacella,[3] Marc C. A. Stuart,[1] John Y. de Boer,[1] Giuseppe Portale,[3] and Ben L. Feringa*[1]

[1] Centre for System Chemistry, Stratingh Institute for Chemistry, University of Groningen, 9747 AG Groningen, The Netherlands
[2] School of Fashion and Textiles, The Hong Kong Polytechnic University, Hong Kong, 999077, China
[3] Macromolecular Chemistry and New Polymeric Materials, Zernike Institute for Advanced Materials, University of Groningen, 9747 AG Groningen, The Netherlands

*Corresponding authors: shaoyu.chen@polyu.edu.hk; b.l.feringa@rug.nl

1. Supplementary Methods: Synthesis and Characterization

1.1 Synthetic route of overcrowded alkene SA

1.1.1 Synthesis of upper-half hydrazone **6**

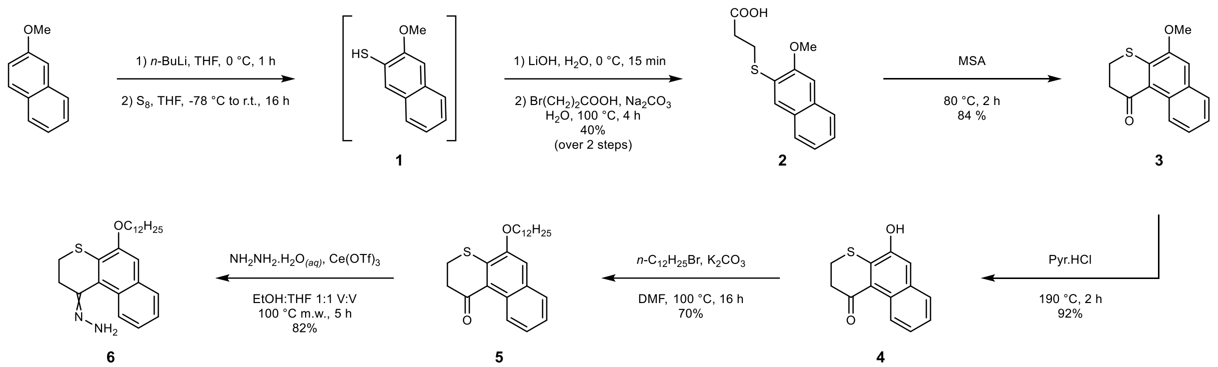

Supplementary Fig. 1 | Synthesis scheme of upper-half precursor 6. Schematic illustration of the synthetic route of the hydrazone **6**.

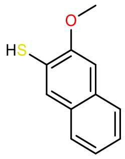

3-Methoxynaphthalene-2-thiol (**1**)

This procedure is a modified version based on our previous method.[1] Under a nitrogen atmosphere, in a 500 mL three-neck round-bottom flask, 2-methoxynaphthalene (8.032 g, 49.76 mmol, 1.0 eq.) was dissolved in dry THF (150 mL, 0.35 M) and the solution was placed at 0 °C. Then, a solution of *n*-butyllithium (1.6 M in *n*-Hex, 37.0 mL, 59.2 mmol, 1.2 eq.) was slowly added on the mixture before to be stirred at 0 °C during 1 h. The flask was placed at -78 °C in dry iced acetone bath before to add carefully cyclooctasulfur (2.309 g, 9.004 mmol, 0.18 eq.) as a solid. The mixture was slowly warmed to room temperature and stirred overnight. The reaction was quenched carefully with an aqueous solution of NaOH (1 M, 150 mL), and the resulting aqueous layer was washed with Et_2O (150 mL). The aqueous phase was acidified with an aqueous solution of HCl (3 M, 150 mL), and the product was extracted with Et_2O (3 x 200 mL). The combined organic layer was washed with brine (300 mL), dried with Na_2SO_4, and concentrated under vacuum to obtain a rust-brown solid as a crude (8.104 g), containing isomers and their corresponding disulfides. The compound was used in the next step without further purification. Note: This compound is highly unstable and oxidizes quickly to form its corresponding disulfide. Therefore, it is advised to start the next step immediately after the isolation of the crude product.

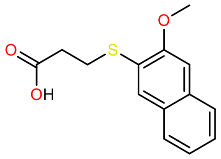

3-((3-Methoxynaphthalen-2-yl)thio)propanoic acid (**2**)

Under a nitrogen atmosphere, in a 500 mL three-neck round-bottom flask, crude thiophenol **1** (8.104 g) and lithium hydroxide (0.916 g, 38.2 mmol, 0.75 eq.) were dissolved in ice-cold water (100 mL, 0.5 M) and the mixture was stirred for 15 min. Separately, 3-bromopropanoic acid (5.320 g, 33.73 mmol, 0.75 eq.) and sodium carbonate (4.001 g, 37.74 mmol, 0.75 eq.) were dissolved in ice-cold water (100 mL, 0.35 M) and the mixture was stirred for 15 min. The solution containing carboxylate was added to the thiophenolate solution, and the mixture was heated at 100 °C and stirred for 4 h. The resulting mixture

was washed with EtOAc (200 mL) to remove impurities. Then, the aqueous layer was acidified with an aqueous solution of HCl (3 M, 200 mL), and the product was extracted with EtOAc (2 x 200 mL). The combined organic phase was washed with brine (200 mL), dried with Na_2SO_4, and concentrated under vacuum to obtain a brown solid as a crude. The product was recrystallized from a mixture of *n*-Hept:EtOAc (7:3 V:V) before being filtered and washed with a minimum amount of cold *n*-Pent to obtain the purified product as beige crystals (5.229 g, 19.93 mmol, 40% over 2 steps). ^{1}H NMR (600 MHz, DMSO-d_6): δ = 12.39 (br s, 1H), 7.80 (dd, J = 7.9, 1.2 Hz, 1H), 7.78 (dd, J = 8.2, 1.2 Hz, 1H), 7.71 (s, 1H), 7.40 (ddd, J = 8.1, 6.8, 1.3 Hz, 1H), 7.34 (ddd, J = 8.1, 6.8, 1.3 Hz, 1H), 7.32 (s, 1H), 3.92 (s, 3H), 3.20 (t, J = 7.1 Hz, 2H), 2.64 (t, J = 7.0 Hz, 2H) ppm; ^{13}C NMR (151 MHz, DMSO-d_6): δ = 172.8, 154.3, 132.0, 128.8, 127.1, 126.5, 126.4, 125.6, 124.4, 124.0, 105.3, 55.9, 33.1, 25.6 ppm; IR (FT-IR): ν = 3300–2300 (br w, O–$H_{carboxylic\ acid}$), 3027 (w, C–$H_{aromatic}$), 2967, 2942, 2917 (m, C–H_{alkane}), 1700 (s, C=$O_{carboxylic\ acid}$), 1588 (m, C=$C_{aromatic}$) cm^{-1}; HRMS (ESI neg.) calculated for $C_{14}H_{13}O_3S$ ([M - H]$^-$): 261.0591, found 261.0595.

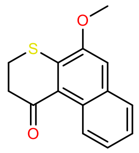

5-Methoxy-2,3-dihydro-1*H*-benzo[*f*]thiochromen-1-one (**3**)

Under a nitrogen atmosphere, in a 250 mL round-bottom flask, carboxylic acid **2** (2.173 g, 8.282 mmol, 1.0 eq.) was dissolved in methanesulfonic acid (40 mL, 0.2 M). The solution was heated at 80 °C and stirred for 2 h. Then, the mixture was carefully poured into water (100 mL) and ice (100 g), and the product was extracted with DCM (2 x 200 mL). The combined organic phase was washed with an aqueous solution of NaOH (1 M, 200 mL) and brine (200 mL), dried with $MgSO_4$, and concentrated under vacuum to obtain a dark brown oil as a crude. The product was purified by flash chromatography (SiO_2, *n*-Pent:EtOAc 19:1 V:V) to obtain the purified product as a pale yellow viscous oil (1.694 g, 6.934 mmol, 84%). ^{1}H NMR (600 MHz, $CDCl_3$): δ = 9.07–9.02 (m, 1H), 7.70–7.68 (m, 1H), 7.47 (ddd, J = 8.6, 6.9, 1.6 Hz, 1H), 7.42 (ddd, J = 8.0, 6.8, 1.3 Hz, 1H), 7.20 (s, 1H), 4.02 (s, 3H), 3.29–3.23 (m, 2H), 3.11–3.06 (m, 2H) ppm; ^{13}C NMR (151 MHz, $CDCl_3$): δ = 196.5, 152.7, 139.1, 132.0, 128.0, 127.2, 127.1, 126.7, 126.1, 126.1, 110.2, 56.4, 40.9, 25.4 ppm; IR (FT-IR): ν = 3062 (w, C–$H_{aromatic}$), 2979, 2939, 2913, 2836 (m, C–H_{alkane}), 1662 (s, C=O_{ketone}), 1596 (m, C=$C_{aromatic}$) cm^{-1}; HRMS (ESI neg.) calculated for $C_{14}H_{11}O_2S$ ([M - H]$^-$): 243.0485, found 243.0484.

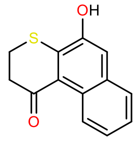

5-Hydroxy-2,3-dihydro-1*H*-benzo[*f*]thiochromen-1-one (**4**)

Under a nitrogen atmosphere, thiochromanone **3** (1.694 g, 6.934 mmol, 1.0 eq.) was placed in a 250 mL round-bottom flask with pyridinium chloride (25.25 g, 218.5 mmol, 30 eq.) and the mixture was heated at 190 °C and stirred for 2 h. Then, water (200 mL) was carefully added immediately before cooling down and the product was extracted with EtOAc (200 mL). The organic phase was washed with an aqueous solution of HCl (3 M, 3 x 100 mL) and brine (200 mL), dried with Na_2SO_4, and concentrated under vacuum to obtain the expected product as a light green powder without further purification (1.477 g, 6.414 mmol, 92%). ^{1}H NMR (600 MHz, DMSO-d_6): δ = 10.73 (br s, 1H), 8.95–8.90 (m, 1H), 7.72–7.68 (m, 1H), 7.40–7.34 (m, 2H), 7.32 (s, 1H), 3.30–3.25 (m, 2H), 2.99–2.95 (m, 2H) ppm; ^{13}C NMR (151 MHz, DMSO-d_6): δ = 196.3, 150.6, 138.7, 131.7, 126.7, 126.6, 126.2, 125.5, 125.4, 125.3, 112.9, 40.4, 24.2 ppm; IR (FT-IR): ν = 3050 (br m, O–H_{phenol}), 2947 (m, C–H_{alkane}), 1631 (m, C=O_{ketone}), 1593 (s, C=$C_{aromatic}$) cm^{-1}; HRMS (ESI neg.) calculated for $C_{13}H_9O_2S$ ([M - H]$^-$): 229.0329, found 229.0330.

Note: This phenol may oxidize upon air exposure. Therefore, it is advised to use it for the next step immediately after isolation.

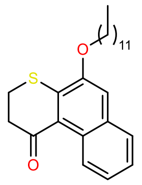

5-(Dodecyloxy)-2,3-dihydro-1*H*-benzo[*f*]thiochromen-1-one (**5**)

Under a nitrogen atmosphere, in a 250 mL three-neck round-bottom flask, phenol **4** (1.460 g, 6.340 mmol, 1.0 eq.) and anhydrous potassium carbonate (4.996 g, 36.15 mmol, 5.0 eq.) were dissolved in dry DMF (50 mL, 0.15 M). Then, 1-bromododecane (4.5 mL, 19 mmol, 3.0 eq.) was added to the solution, and the mixture was heated at 100 °C and stirred overnight. The mixture was cooled down at room temperature, and the reaction was quenched with water (250 mL) before extraction of the product with EtOAc (250 mL). The organic layer was washed with an aqueous solution of LiCl (10 wt.%, 5 x 150 mL), dried with $MgSO_4$, and concentrated under vacuum to obtain a brown oil as a crude. The product was purified by flash chromatography (SiO_2, *n*-Pent:Et_2O 19:1 V:V) to obtain the pure product as a yellow paste (1.767 g, 4.433 mmol, 70%). ^{1}H NMR (600 MHz, $CDCl_3$): δ = 9.08–9.05 (m, 1H), 7.65 (dd, J = 8.0, 1.6 Hz, 1H), 7.46 (ddd, J = 8.6, 6.8, 1.5 Hz, 1H), 7.40 (ddd, J = 8.0, 6.8, 1.3 Hz, 1H), 7.17 (s, 1H), 4.14 (t, J = 6.4 Hz, 2H), 3.26–3.23 (m, 2H), 3.09–3.05 (m, 2H), 1.93–1.87 (m, 2H), 1.58–1.52 (m, 2H), 1.43–1.37 (m, 2H), 1.37–1.21 (m, 14H), 0.89 (t, J = 7.0 Hz, 3H) ppm; ^{13}C NMR (151 MHz, $CDCl_3$): δ = 196.6, 152.1, 139.7, 132.0, 127.9, 127.1, 126.9, 126.5, 126.0, 126.0, 110.9, 69.3, 40.8, 32.0, 29.8, 29.8, 29.7, 29.7, 29.5, 29.5, 29.1, 26.2, 25.3, 22.8, 14.3 ppm; IR (FT-IR): ν = 3053 (w, C–$H_{aromatic}$), 2954, 2921, 2848 (s, C–H_{alkane}), 1661 (s, C=O_{ketone}), 1596 (m, C=$C_{aromatic}$) cm^{-1}; HRMS (ESI pos.) calculated for $C_{25}H_{35}O_2S$ ([M + H]$^+$): 399.2352, found 399.2343.

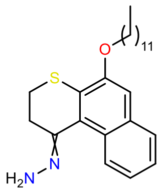

(5-(Dodecyloxy)-2,3-dihydro-1*H*-benzo[*f*]thiochromen-1-ylidene)hydrazine (**6**)

Under air atmosphere, in a 35 mL pressure tube containing a stirring egg, thiochromanone **5** (0.416 g, 0.981 mmol, 1.0 eq.) and cerium(III) trifluoromethanesulfonate (48 mg, 82 µmol, 0.08 eq.) were dissolved in a mixture of EtOH:THF (1:1 V:V, 14 mL, 0.075 M). Then, an aqueous solution of hydrazine monohydrate (80 wt.%, 1.8 mL, 29 mmol, 30 eq.) was added to the solution and the tube was placed for 10 min in an ultrasound bath. The tube was heated at 100 °C for 5 h using microwaves in nitrogen medium, before being placed in the fridge overnight. The solvent was removed under vacuum and the product was purified by flash chromatography (SiO_2, *n*-Pent:EtOAc 3:1 V:V) to obtain both isomers as a pale yellow oil (0.354 g, 0.806 mmol, 82%, ratio *E*:*Z* 17:3). ^{1}H NMR (600 MHz, $CDCl_3$): [*(E)*-**6**] δ = 8.57 (dd, J = 7.9, 1.8 Hz, 1H), 7.65 (dd, J = 7.3, 1.9 Hz, 1H), 7.37–7.31 (m, 2H), 7.01 (s, 1H), 5.52 (br s, 2H), 4.12 (t, J = 6.7 Hz, 2H), 3.06–3.01 (m, 2H), 3.00–2.96 (m, 2H), 1.96–1.86 (m, 2H), 1.56–1.49 (m, 2H), 1.43–1.36 (m, 2H), 1.36–1.21 (m, 14H), 0.88 (t, J = 6.9 Hz, 3H) ppm, [*(Z)*-**6**] δ = 7.71–7.68 (m, 1H), 7.53–7.49 (m, 1H), 7.40–7.37 (m, 2H), 7.10 (s, 1H), 5.24 (br s, 2H), 4.14 (t, J = 6.5 Hz, 2H), 3.37 (dt, J = 11.9, 5.4 Hz, 1H), 3.31–3.21 (m, 1H), 3.14–3.04 (m, 1H), 2.76 (dt, J = 12.3, 5.5 Hz, 1H), 1.93–1.84 (m, 2H), 1.58–1.48 (m, 2H), 1.44–1.38 (m, 2H), 1.37–1.21 (m, 14H), 0.88 (t, J = 6.6 Hz, 3H) ppm; ^{13}C NMR (151 MHz, $CDCl_3$): [*(E)*-**6**] δ = 153.2, 146.1, 133.7, 131.1, 130.0, 127.5, 127.2, 127.1, 125.8, 124.5, 106.3, 69.2, 32.2, 30.4, 30.0, 30.0, 29.9, 29.9, 29.7, 29.7, 29.4, 27.0, 26.4, 23.0, 14.4 ppm, [*(Z)*-**6**] δ = 153.8, 144.7, 131.8, 131.3, 127.6, 125.9, 125.8, 125.7, 124.8, 123.8, 107.3, 69.3, 32.3, 32.2,

30.0, 30.0, 30.0, 29.9, 29.7, 29.7, 29.5, 29.4, 26.4, 23.0, 14.5 ppm; IR (FT-IR): ν = 3395, 3299, 3223 (br w, N–H*hydrazone*), 3054 (w, C–H*aromatic*), 2923, 2854 (s, C–H*alkane*), 1613 (w, C=N*hydrazone*), 1592, 1557 (m, C=C*aromatic*) cm^{-1}; HRMS (ESI pos.): calculated for $C_{25}H_{37}N_2OS$ ([M + H]$^+$): 413.2621, found 413.2626.
Note: On the ^{1}H NMR spectrum (see Supplementary Fig. 28), both isomers are present. The integrations have been done for *(E)*-**6** only.

1.1.2 Synthesis of lower-half thioketone **8**

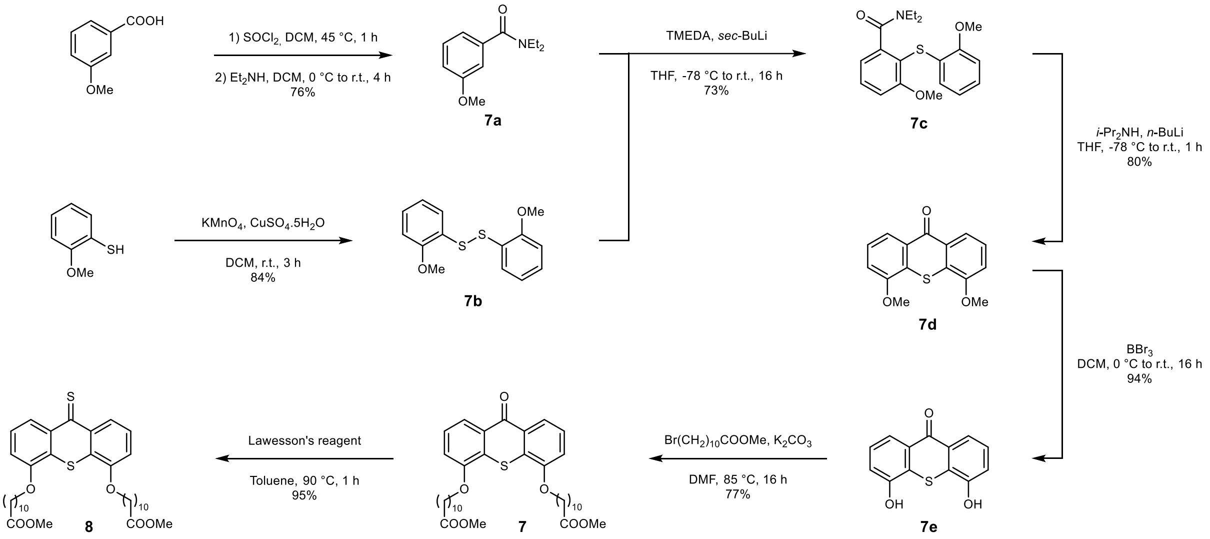

Supplementary Fig. 2 | Synthetic scheme of lower-half precursor 8. Schematic illustration of the synthetic route of the thioketone **8**.

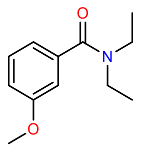

N,N-Diethyl-3-methoxybenzamide (**7a**)

This procedure is a modified version based on our previous method.[1] Under nitrogen atmosphere, in a 100 mL three-neck round-bottom flask, 3-methoxybenzoic acid (4.970 g, 32.34 mmol, 1.0 eq.) was dissolved in dry dichloromethane (30 mL, 1.0 M). Then, thionyl chloride (6.0 mL, 80 mmol, 2.5 eq.) was added slowly at room temperature before to be heated at 45 °C and stirred during 1 h. The mixture was cooled down to room temperature, concentrated into vacuum, placed under nitrogen atmosphere and redissolved in dry dichloromethane (30 mL, 1.0 M). In same time, under nitrogen atmosphere, in a 250 mL three-neck round-bottom, diethylamine (13.5 mL, 131 mmol, 4.0 eq.) was dissolved in dry dichloromethane (30 mL, 4.0 M), before to be placed at 0 °C. Then, the 3-methoxybenzoyl chloride solution was added carefully on diethylamine solution and the resulting mixture was slowly warm to room temperature and stirred during 4 h. The mixture was quenched with distilled water (30 mL) before to extract the product. The organic layer was washed with an aqueous solution of HCl (10 wt.%, 2 x 30 mL), a saturated aqueous solution of $NaHCO_3$ (2 x 30 mL) and brine (30 mL), dried with $MgSO_4$, and concentrated into vacuum to obtain the pure product as a yellow oil (5.172 g, 24.70 mmol, 76%). ^{1}H NMR (600 MHz, $CDCl_3$): δ = 7.28 (t, J = 7.8 Hz, 1H), 6.94–6.87 (m, 3H), 3.81 (s, 3H), 3.53 (br s, 2H), 3.25 (br s, 2H), 1.23 (br s, 3H), 1.11 (br s, 3H) ppm; ^{13}C NMR (151 MHz, $CDCl_3$): δ = 171.1, 159.7, 138.6, 129.6, 118.5, 115.1, 111.8, 55.4, 43.4 (br), 39.3 (br), 14.4 (br), 13.0 (br) ppm; IR (FT-IR): ν = 3060 (w, C–H*aromatic*), 2973, 2936, 2877, 2837 (m, C–H*alkane*), 1628 (s, C=O*amide*), 1579 (s, C=C*aromatic*) cm^{-1}; HRMS (ESI pos.) calculated for $C_{12}H_{18}NO_2$ ([M + H]$^+$): 208.1332, found 208.1336.

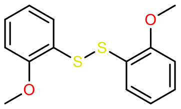

1,2-Bis(2-methoxyphenyl)disulfane (**7b**)

This procedure is a modified version based on our previous method.[1] Under air atmosphere, in a 500 mL round-bottom flask, 2-methoxybenzenethiol (21.5 mL, 171 mmol, 1.0 eq.) was dissolved in dry dichloromethane (170 mL, 1.0 M). Then, potassium permanganate (61.60 g, 389.8 mmol, 2.2 eq.) and copper(II) sulfate pentahydrate (61.20 g, 245.1 mmol, 1.4 eq.) were added as solid to the solution, before to be stirred at room temperature during 3 h. Then, the resulting mixture was filtered on a plug of celite, and the "cake" was washed with DCM. The filtrate was concentrated under vacuum and the compound was recrystallized in a mixture of *n*-Hept:$CHCl_3$ (7:3 V:V) to obtain the pure product as gray crystals (21.67 g, 76.30 mmol, 84%). ^{1}H NMR (600 MHz, $CDCl_3$): δ = 7.59 (ddd, J = 7.8, 1.4, 1.2 Hz, 2H), 7.21 (dt, J = 7.8, 1.7 Hz, 2H), 6.94 (dt, J = 7.6, 1.2 Hz, 2H), 6.87 (dd, J = 8.2, 1.2 Hz, 2H), 3.91 (s, 6H) ppm; ^{13}C NMR (151 MHz, $CDCl_3$): δ = 156.6, 127.8, 127.5, 124.5, 121.3, 110.5, 55.9 ppm; IR (FT-IR): ν = 3058 (w, C–H$_{aromatic}$), 2999, 2966, 2941, 2837 (w, C–H$_{alkane}$), 1578 (s, C=C$_{aromatic}$) cm^{-1}; HRMS (ESI neg.) calculated for $C_{14}H_{13}O_2S_2$ ([M - H]$^-$): 277.0362, found 277.0358.

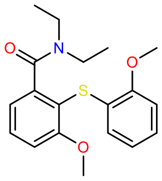

N,N-Diethyl-3-methoxy-2-((2-methoxyphenyl)thio)benzamide (**7c**)

This procedure is a modified version based on our previous method.[1] Under nitrogen atmosphere, in a 100 mL three-neck round-bottom flask, anhydrous tetrahydrofurane (20 mL) was placed at -78 °C before to add *N,N,N',N'*-tetramethylethylenediamine (0.33 mL, 2.2 mmol, 1.1 eq.). A solution of *sec*-butyllithium (1.4 M in *n*-Hex, 1.6 mL, 2.2 mmol, 1.1 eq.) was slowly added to the solution before to be stirred at -78 °C during 1 h. The solution of amide **7a** (2.0 M in anhydrous THF, 0.419 g, 2.00 mmol, 1.0 eq.) was added dropwise to the reaction mixture and stirred at -78 °C during 1 h. Then, disulfane **7b** (0.584 g, 2.03 mmol, 1.05 eq.) was added to the reaction mixture as a solid, before to slowly warm at room temperature and stirred overnight. The reaction was quenched with Et_2O (40 mL) and stirred during 10 min. The resulting solution was washed with an aqueous solution of NaOH (1 M, 40 mL) and brine (40 mL), dried with $MgSO_4$ and concentrated under vacuum to obtain a brown oil as a crude. The compound was purified by flash chromatography (SiO_2, *n*-Pent:EtOAc 4:1 V:V) to obtain the pure product as an off-white solid (0.507 g, 1.45 mmol, 73%). ^{1}H NMR (600 MHz, $CDCl_3$): δ = 7.43 (dd, J = 8.3, 7.5 Hz, 1H), 7.04 (ddd, J = 8.0, 7.3, 1.7 Hz, 1H), 6.95–6.92 (m, 2H), 6.79 (dd, J = 8.2, 1.2 Hz, 1H), 6.73 (dt, J = 7.5, 1.2 Hz, 1H), 6.68 (dd, J = 7.8, 1.7 Hz, 1H), 3.87 (s, 3H), 3.75 (s, 3H), 3.68 (dq, J = 14.1, 7.1 Hz, 1H), 3.35 (dq, J = 14.0, 7.1 Hz, 1H), 3.11 (dq, J = 14.4, 7.2 Hz, 1H), 3.00 (dq, J = 14.2, 7.0 Hz, 1H), 1.19 (t, J = 7.1 Hz, 3H), 0.98 (t, J = 7.1 Hz, 3H) ppm; ^{13}C NMR (151 MHz, $CDCl_3$): δ = 168.8, 160.7, 155.7, 145.3, 131.3, 127.1, 125.9, 125.0, 121.1, 118.8, 116.2, 111.4, 110.3, 56.2, 55.9, 42.8, 38.7, 14.0, 12.6 ppm; IR (FT-IR): ν = 3069, 3019 (w, C–H$_{aromatic}$), 3002, 2978, 2969, 2938, 2875, 2837 (m, C–H$_{alkane}$), 1627 (s, C=O$_{amide}$), 1582, 1569 (s, C=C$_{aromatic}$) cm^{-1}; HRMS (ESI pos.) calculated for $C_{19}H_{23}NO_3SNa$ ([M + Na]$^+$): 368.1291, found 368.1291.

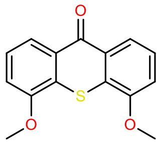

4,5-Dimethoxy-9*H*-thioxanthen-9-one (**7d**)

This procedure is a modified version based on our previous method.[1] Under nitrogen atmosphere, in a 500 mL three-neck round-bottom flask, diisopropylamine (4.4 mL, 31 mmol, 5.0 eq.) was dissolved in anhydrous THF (120 mL) before to be placed at -78 °C. Then, a solution of *n*-butyllithium (1.6 M in *n*-Hex, 19.6 mL, 31.4 mmol, 5.0 eq.) was added slowly to the solution before to warm it at room temperature and stirred for 30 min. The mixture was then placed again at -78 °C and the solution of amide **7c** (0.1 M in anhydrous THF, 2.100 g, 5.836 mmol, 1.0 eq.) was added dropwise before to be warmed at room temperature and stirred during 1 h. The reaction was carefully quenched with a saturated aqueous solution of NH_4Cl (100 mL) and the product was extracted with DCM (2 x 100 mL). Thecombined organic layer was washed with brine (100 mL), dried with $MgSO_4$, and concentrated under vacuum to obtain a brownish cotton-like solid as a crude. The compound was subjected to trituration in a mixture of *n*-Hept:EtOAc (3:2 V:V) and the suspension was filtered and washed with cold n-Hept to obtain a yellow cotton-like solid. Then, the compound was recrystallized in EtOAc to obtain the pure product as yellow needle-like crystals (1.288 g, 4.682 mmol, 80%). ^{1}H NMR (600 MHz, $CDCl_3$): δ = 8.24 (dd, J = 8.1, 1.1 Hz, 2H), 7.43 (t, J = 8.0 Hz, 2H), 7.12 (dd, J = 7.9, 1.2 Hz, 2H), 4.04 (s, 6H) ppm; ^{13}C NMR (151 MHz, $CDCl_3$): δ = 180.4, 155.0, 130.1, 127.8, 126.1, 121.6, 112.2, 56.6 ppm; IR (FT-IR): ν = 3078, 3015 (w, C–H$_{aromatic}$), 2971, 2941, 2839 (m, C–H$_{alkane}$), 1627 (m, C=O$_{ketone}$), 1591, 1569 (m, C=C$_{aromatic}$) cm^{-1}; HRMS (ESI pos.) calculated for $C_{15}H_{12}O_3SNa$ ([M + Na]$^+$): 295.0399, found 295.0394.

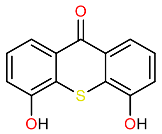

4,5-Dihydroxy-9*H*-thioxanthen-9-one (**7e**)

This procedure is a modified version based on our previous method.[1] Under nitrogen atmosphere, in a 500 mL three-neck round-bottom flask, thioxanthone **7d** (1.600 g, 5.817 mmol, 1.0 eq.) was dissolved in anhydrous DCM (180 mL, 0.035 M) before to be placed at 0 °C. Then, a solution of boron bromide (1.0 M in DCM, 27 mL, 27 mmol, 4.5 eq.) was added dropwise to the solution, and the resulting mixture was slowly warm to room temperature and stirred overnight. The reaction was carefully quenched with a saturated solution of $NaHCO_3$ (60 mL) and the product was extracted with DCM (2 x 30 mL). The combined organic layer was washed with brine (120 mL), dried with Na_2SO_4 and concentrated under vacuum in order to obtain the pure product as a green powder (1.348 g, 5.464 mmol, 94%). ^{1}H NMR (600 MHz, DMSO-d_6): δ = 11.02 (br s, 2H), 7.95 (dd, J = 8.0, 1.3 Hz, 2H), 7.39 (t, J = 7.9 Hz, 2H), 7.21 (dd, J = 7.8, 1.2 Hz, 2H) ppm; ^{13}C NMR (151 MHz, DMSO-d_6): δ = 179.4, 153.1, 129.3, 126.4, 125.3, 119.3, 116.5 ppm; IR (FT-IR): ν = 3042 (br m, O–H$_{phenol}$), 1616 (m, C=O$_{ketone}$), 1591, 1558 (s, C=C$_{aromatic}$) cm^{-1}; HRMS (APCI pos.) calculated for $C_{13}H_9O_3S$ ([M + H]$^+$): 245.0267, found 245.0267.

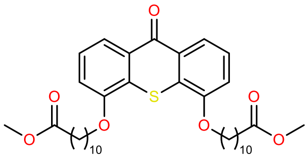

Dimethyl 11,11'-((9-oxo-9*H*-thioxanthene-4,5-diyl)bis(oxy))diundecanoate (**7**)

This procedure is a modified version based on our previous method.[2] Under nitrogen atmosphere, in a 250 mL three-neck round-bottom flask, thioxanthone **7e** (1.500 g, 6.080 mmol, 1.0 eq.) and anhydrous potassium carbonate (4.201 g, 30.40 mmol, 5.0 eq.) were placed in anhydrous DMF (40 mL, 0.15 M). Then, methyl 11-bromoundecanoate (3.9 mL, 15 mmol, 2.5 eq.) was added to the solution and the resulting mixture was heated at 85 °C and stirred overnight. The solution was cooled down to room temperature, before to be quenched with water (200 mL). The product was extracted with EtOAc (3 x 100 mL), and the combined organic layer was washed with an aqueous solution of LiCl (10 wt.%, 5 x

100 mL), dried with $MgSO_4$ and concentrated under vacuum to obtain a brown oil as a crude. The crude has been dissolved in a hot mixture of *n*-Hept:EtOAc (1:1 V:V) and placed at -20 °C overnight to precipitate the pure product as a yellow solid (3.145 g, 4.662 mmol, 77%). ^{1}H NMR (600 MHz, $CDCl_3$): δ = 8.23 (dd, J = 8.1, 1.1 Hz, 2H), 7.41 (t, J = 8.0 Hz, 2H), 7.12 (dd, J = 7.9, 1.1 Hz, 2H), 4.18 (t, J = 6.5 Hz, 2H), 3.65 (s, 6H), 2.28 (t, J = 7.5 Hz, 4H), 1.97–1.89 (m, 4H), 1.67–1.57 (m, 8H), 1.43–1.37 (m, 4H), 1.37–1.25 (m, 10H) ppm; ^{13}C NMR (151 MHz, $CDCl_3$): δ = 180.6, 174.4, 154.5, 130.2, 128.5, 126.0, 121.4, 113.2, 69.6, 51.6, 34.2, 29.7, 29.6, 29.4, 29.4, 29.3, 29.2, 26.2, 25.1 ppm; IR (FT-IR): ν = 3063 (w, C–H$_{aromatic}$), 2927, 2915, 2850 (s, C–H$_{alkane}$), 1730 (s, C=O$_{ester}$), 1640 (s, C=O$_{ketone}$), 1595, 1575 (m, C=C$_{aromatic}$) cm^{-1}; HRMS (ESI pos.) calculated for $C_{37}H_{53}O_7S$ ([M + H]$^+$): 641.3507, found 641.3501.

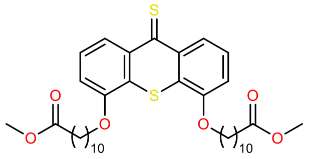

Dimethyl 11,11'-((9-thioxo-9*H*-thioxanthene-4,5-diyl)bis(oxy))diundecanoate (**8**)

Under a nitrogen atmosphere, in a 100 mL three-neck round-bottom flask, thioxanthone **7** (0.600 g, 0.899 mmol, 1.0 eq.) and Lawesson's reagent (0.900 g, 2.16 mmol, 2.5 eq.) were dissolved in dry toluene (25 mL, 0.035 M) and the mixture was heated at 90 °C and stirred for 1 h. After cooling down to room temperature, the solvent was removed under vacuum. The product was purified by column chromatography (SiO_2, *n*-Pent:EtOAc 17:3 V:V) to obtain the pure product as a dark green solid (0.851 g, 0.842 mmol, 95%). ^{1}H NMR (600 MHz, $CDCl_3$): δ = 8.66 (dd, J = 8.5, 1.1 Hz, 2H), 7.36 (t, J = 8.2 Hz, 2H), 7.11 (dd, J = 7.9, 1.1 Hz, 2H), 4.19 (t, J = 6.5 Hz, 4H), 3.65 (s, 6H), 2.29 (t, J = 7.6 Hz, 4H), 1.99–1.88 (m, 4H), 1.67–1.57 (m, 8H), 1.45–1.22 (m, 20H) ppm; ^{13}C NMR (151 MHz, $CDCl_3$): δ = 213.0, 176.4, 156.6, 140.5, 128.5, 127.3, 126.1, 114.3, 71.8, 53.6, 36.2, 31.7, 31.6, 31.5, 31.4, 31.3, 31.2, 28.2, 27.1 ppm; IR (FT-IR): ν = 3078 (w, C–H$_{aromatic}$), 2915, 2851 (s, C–H$_{alkane}$), 1734 (s, C=O$_{ester}$), 1595, 1568 (m, C=C$_{aromatic}$) cm^{-1}; HRMS (ESI pos.): calculated for $C_{37}H_{53}O_6S_2$ ([M + H]$^+$): 657.3278, found 657.3272.

Note: This thioketone can be stored under nitrogen atmosphere at -20 °C for at least 2 weeks.

1.1.3 Synthesis of overcrowded alkene **SA**

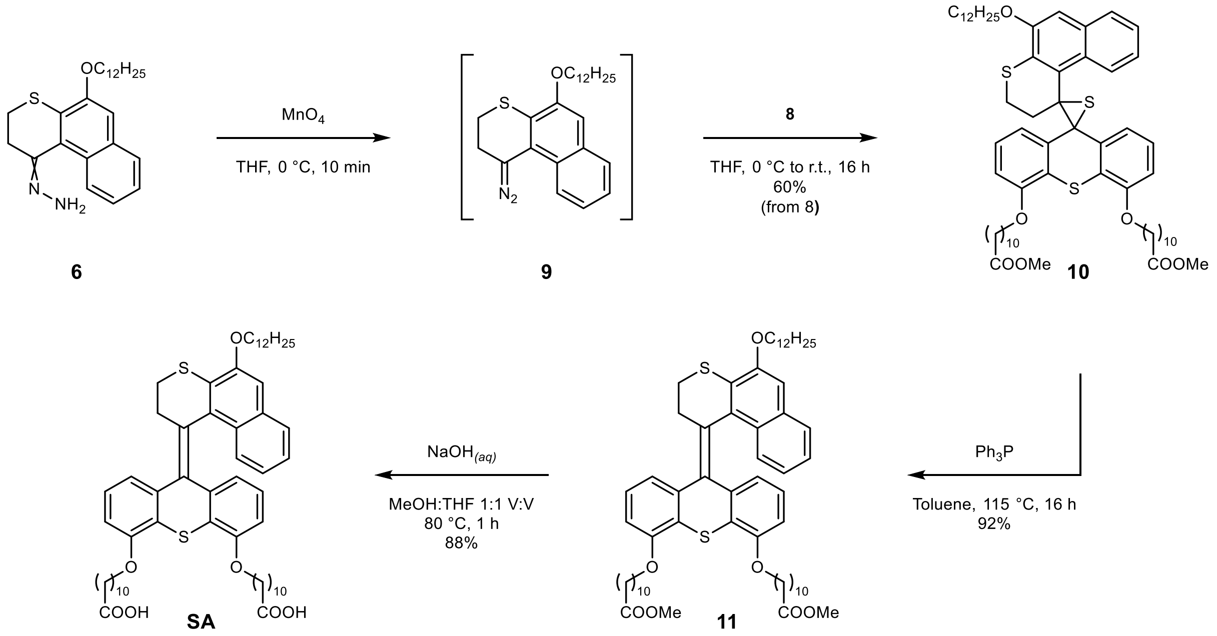

Supplementary Fig. 3 | Synthetic scheme of overcrowded alkene SA. Schematic illustration of the Barton-Kellogg coupling reaction and hydrolysis of ester groups.

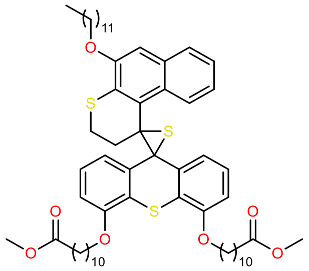

Dimethyl 11,11'-((5-(dodecyloxy)-2,3-dihydrodispiro[benzo[*f*]thiochromene-1,2'-thiirane-3',9''-thioxanthene]-4'',5''-diyl)bis(oxy))diundecanoate (**10**)

Under a nitrogen atmosphere, manganese(IV) dioxide (Fluka, 1.031 g, 11.85 mmol, 25 eq.) was placed in a dried Schlenk flask with a stirring egg. The Schlenk flask was connected by a cannula to a second dried Schlenk flask containing a stirring egg. The two Schlenk flasks were covered with aluminum foil and placed at 0 °C. Subsequently, hydrazone **6** (0.336 g, 0.733 mmol, 1.5 eq.) and thioketone **8** (0.350 g, 0.474 mmol, 1.0 eq.) were separately dissolved in cold dry THF (5 mL for each, 0.1 M) under nitrogen atmosphere. The cold solution of hydrazone **6** was added to the Schlenk flask containing the manganese(IV) dioxide and the mixture was stirred at 0 °C for 10 min, before to turn off the stirring and let the solid settle down during 5 min. Then, the dark pink resulting solution of diazo **9** was transfered into the empty Schlenk flask (using a cannula and nitrogen pressure, filtering solid with filter paper) and the cold solution of thioketone **8** was dropwise added to the solution. The resulting mixture was slowly warmed to room temperature and stirred overnight, still covered with aluminum foil. The solvent was evaporated in vacuum and the product was purified by flash chromatography (SiO_2, *n*-Pent:EtOAc 19:1 V:V) to obtain the purified product as a yellow oil (0.247 g, 0.202 mmol, 60% from thioketone **8**). ^{1}H NMR (600 MHz, $CDCl_3$): δ = 8.78 (d, J = 8.5 Hz, 1H), 7.72 (dd, J = 8.1, 1.1 Hz, 1H), 7.50 (dd, J = 8.0, 1.5 Hz, 1H), 7.34 (ddd, J = 8.5, 6.7, 1.5 Hz, 1H), 7.30 (ddd, J = 7.9, 6.7, 1.3 Hz, 1H), 7.21 (t, J = 8.0 Hz, 1H), 6.87 (dd, J = 8.0, 1.1 Hz, 1H), 6.72 (s, 1H), 6.43 (dd, J = 8.1, 1.1 Hz, 1H), 6.34 (dd, J = 8.1, 1.1 Hz, 1H), 6.16 (t, J = 8.0 Hz, 1H), 4.19–4.13 (m, 1H), 4.05–4.00 (m, 1H), 3.93–3.80 (m, 4H), 3.66 (s, 6H), 2.63–2.54 (m, 2H), 2.54–2.48 (m, 1H), 2.30 (t, J = 7.6 Hz, 4H), 2.28–2.22 (m, 1H), 1.97–1.85 (m, 2H), 1.85–1.69 (m, 4H), 1.68–1.47 (m, 8H), 1.47–1.22 (m, 38H), 0.89 (t, J = 7.0 Hz, 3H) ppm; ^{13}C NMR (151 MHz, $CDCl_3$): δ = 174.4, 174.4, 154.4, 153.6, 153.0, 133.6, 132.7, 131.2, 131.2, 129.2, 127.1, 126.1, 125.0, 124.6, 124.5, 124.2, 123.8, 123.4, 122.7, 121.9, 110.2, 110.1, 106.2, 69.7, 69.5, 69.4, 60.0, 58.8, 51.5, 36.5, 34.2, 32.1, 29.9, 29.8, 29.7, 29.7, 29.7, 29.6, 29.6, 29.6, 29.5, 29.5, 29.5, 29.4, 29.4, 29.3, 29.3, 29.1, 27.6, 26.2, 26.2, 26.1, 25.1, 25.1, 22.8, 14.2 ppm; IR (FT-IR): ν = 3068 (w, C–H$_{aromatic}$), 2923, 2852 (s, C–H$_{alkane}$), 1738 (s, C=O$_{ester}$), 1575 (m, C=C$_{aromatic}$) cm^{-1}; HRMS (ESI pos.) calculated for $C_{62}H_{87}O_7S_3$ ([M + H]$^+$): 1039.5608, found 1039.5607.
Notes: MnO_4 from Fluka gives higher yields. The addition of anhydrous Na_2SO_4 does not seem to have significant effect on the yield.

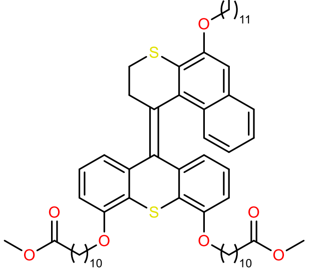

Dimethyl 11,11'-((9-(5-(dodecyloxy)-2,3-dihydro-1*H*-benzo[*f*]thiochromen-1-ylidene)-9*H*-thioxanthene-4,5-diyl)bis(oxy))diundecanoate (**11**)

Under a nitrogen atmosphere, in a 50 mL two-neck round-bottom flask, episulfide **10** (70 mg, 61 µmol, 1.0 eq.) and triphenylphosphine (38 mg, 0.14 mmol, 2.5 eq.) were dissolved in dry toluene (1.8 mL,

0.035 M). Then, the resulting solution was heated at reflux and stirred overnight. The flask was cooled down to room temperature and the solvent was removed under vacuum. The product was isolated by flash chromatography (SiO_2, *n*-Pent:EtOAc 9:1 V:V) to obtain the product as a pale yellow oil (59 mg, 56 µmol, 92%). ^{1}H NMR (600 MHz, CD_2Cl_2): δ = 7.51 (d, *J* = 8.5 Hz, 1H), 7.48 (dd, *J* = 8.1, 1.2 Hz, 1H), 7.33 (t, *J* = 7.9 Hz, 1H), 7.19 (dd, *J* = 7.7, 1.1 Hz, 1H), 7.08 (ddd, *J* = 8.0, 6.7, 1.2 Hz, 1H), 6.99 (s, 1H), 6.91–6.86 (m, 2H), 6.43 (t, *J* = 7.9 Hz, 1H), 6.38 (dd, *J* = 8.2, 1.2 Hz, 1H), 6.19 (dd, *J* = 7.7, 1.2 Hz, 1H), 4.21–4.15 (m, 2H), 4.14–4.09 (m, 2H), 4.04–3.97 (m, 1H), 3.96–3.89 (m, 1H), 3.64 (s, 6H), 3.63–3.53 (m, 2H), 3.52–3.46 (m, 1H), 2.30 (t, *J* = 7.6 Hz, 4H), 2.14 (dt, *J* = 12.0, 6.9 Hz, 1H), 1.96–1.90 (m, 4H), 1.85–1.79 (m, 2H), 1.66–1.49 (m, 10H), 1.48–1.26 (m, 36H), 0.91 (t, *J* = 6.9 Hz, 3H) ppm; ^{13}C NMR (151 MHz, CD_2Cl_2): δ = 174.4, 155.8, 155.1, 153.8, 139.2, 136.5, 136.0, 133.7, 132.4, 132.2, 128.2, 126.7, 126.6, 125.9, 125.0, 124.9, 124.8, 124.6, 124.0, 123.2, 122.0, 119.8, 110.1, 109.7, 105.5, 69.5, 69.1, 54.2, 54.0, 53.8, 53.7, 53.5, 51.6, 34.4, 32.4, 30.3, 30.2, 30.1, 30.1, 30.1, 30.0, 30.0, 29.9, 29.9, 29.9, 29.9, 29.8, 29.7, 29.7, 29.6, 29.6, 28.9, 26.6, 26.5, 26.4, 25.4, 23.1, 14.3 ppm; IR (FT-IR): ν = 3065 (w, C–$H_{aromatic}$), 2925, 2854 (s, C–H_{alkane}), 1740 (s, C=O_{ester}), 1571 (m, C=$C_{aromatic}$) cm^{-1}; HRMS (ESI pos.): calculated for $C_{62}H_{86}O_7S_2Na$ ($[M + Na]^+$): 1029.5707, found 1029.5710.

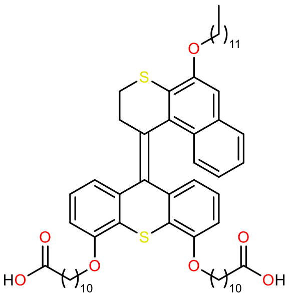

11,11'-((9-(5-(Dodecyloxy)-2,3-dihydro-1*H*-benzo[*f*]thiochromen-1-ylidene)-9*H*-thioxanthene-4,5-diyl)bis(oxy))diundecanoic acid (**SA**)

Under a nitrogen atmosphere, in a 50 mL two-neck round-bottom flask, overcrowded alkene **11** (139 mg, 138 µmol, 1.0 eq.) was dissolved in a mixture of MeOH:THF (1:1 V:V, 9.0 mL, 0.015 M). Then, an aqueous solution of sodium hydroxide (4.0 M, 1.0 mL, 4.0 mmol, 30 eq.) was added to the mixture, and the mixture was heated at 80 °C and stirred for 1 h. After cooling down to room temperature, the volatile solvents were removed under vacuum. The resulting residue was diluted with an aqueous solution of HCl (1 M, 10 mL) and the product was extracted with EtOAc (2 x 10 mL). The organic phase was washed with brine (10 mL), dried with Na_2SO_4, and concentrated under vacuum to obtain a pale yellow oil as a crude. The crude compound was dissolved in a minimum amount of Et_2O, then *n*-Pent was added to the solution until precipitation of the product. The solid was filtered and washed with *n*-Pent to obtain the product as an off-white solid (110 mg, 112 µmol, 88%). ^{1}H NMR (600 MHz, CD_2Cl_2): δ = 7.49 (d, *J* = 8.5 Hz, 1H), 7.46 (d, *J* = 8.1 Hz, 1H), 7.32 (t, *J* = 7.9 Hz, 1H), 7.18 (dd, *J* = 7.7, 0.7 Hz, 1H), 7.07 (ddd, *J* = 7.8, 6.7, 1.0 Hz, 1H), 6.97 (s, 1H), 6.89 (d, *J* = 8.1 Hz, 1H), 6.86 (ddd, *J* = 8.3, 6.7, 1.3 Hz, 1H), 6.42 (t, *J* = 7.9 Hz, 1H), 6.36 (dd, *J* = 8.0, 1.0 Hz, 1H), 6.17 (dd, *J* = 7.7, 1.0 Hz, 1H), 4.20–4.14 (m, 2H), 4.13–4.05 (m, 2H), 4.02–3.95 (m, 1H), 3.93–3.86 (m, 1H), 3.63–3.52 (m, 2H), 3.51–3.45 (m, 1H), 2.35 (t, *J* = 7.3 Hz, 4H), 2.13 (dt, *J* = 11.6, 7.5 Hz, 1H), 1.96–1.87 (m, 4H), 1.85–1.77 (m, 2H), 1.68–1.59 (m, 4H), 1.62–1.47 (m, 4H), 1.47–1.22 (m, 38H), 0.91–0.85 (m, 3H) ppm; ^{13}C NMR (151 MHz, CD_2Cl_2): δ = 180.7, 156.2, 155.6, 154.1, 139.6, 136.9, 136.4, 134.1, 132.8, 132.6, 128.6, 127.1, 127.0, 126.2, 125.4, 125.3, 125.2, 124.9, 124.4, 123.6, 122.4, 120.2, 110.5, 110.2, 105.9, 70.0, 70.0, 69.5, 34.8, 32.8, 30.7, 30.5, 30.5, 30.5, 30.5, 30.3, 30.3, 30.3, 30.3, 30.3, 30.2, 30.2, 30.1, 30.0, 29.8, 29.3, 27.07, 26.9, 26.8, 25.5, 23.5, 14.7 ppm; IR (FT-IR): ν = 3500–2500 (br w, O–$H_{carboxylic\ acid}$), 3068 (w, C–$H_{aromatic}$), 2921, 2852 (s, C–H_{alkane}), 1704 (s, C=$O_{carboxylic\ acid}$), 1575 (m, C=$C_{aromatic}$) cm^{-1}; HRMS (ESI pos.): calculated for $C_{60}H_{83}O_7S_2$ ($[M + H]^+$): 979.5575, found 979.5574.

1.2 Synthetic route of overcrowded alkene BMS

1.2.1 Synthesis strategy

After optimizing the synthesis of the overcrowded alkene **SA**, we prepared the non-symmetric overcrowded alkene **BMS** using a similar procedure (see Fig. 3a and Supplementary Fig. 2). Thiochromanone **3** was converted into the corresponding hydrazone **12**, using standard conditions.[2] The dissymmetric lower-half **14** was obtained in two steps starting from thiosalicylic acid. The starting material was converted with 2-iodoanisole in the presence of potassium carbonate and a catalytic amount of copper powder in refluxing DMF, to afford the benzoic acid derivative **13** by a copper-catalyzed Ullman coupling reaction.[3] Benzoic acid **13** was converted to thioxanthone **14** by acidic cyclization in the presence of methanesulfonic acid. Thioxanthone **14** was transformed into unstable thioketone **15** with Lawesson's reagent. *In situ* oxidation of hydrazone **12** into diazo compound **16** was performed with MnO_2, before the addition of a freshly prepared THF solution of thioketone **15** to provide the coupling to form a mixture of diastereomers of episulfide **17**. Desulfurization of episulfide **17** was performed with triphenylphosphine to afford the bis-methoxy switch **BMS**.

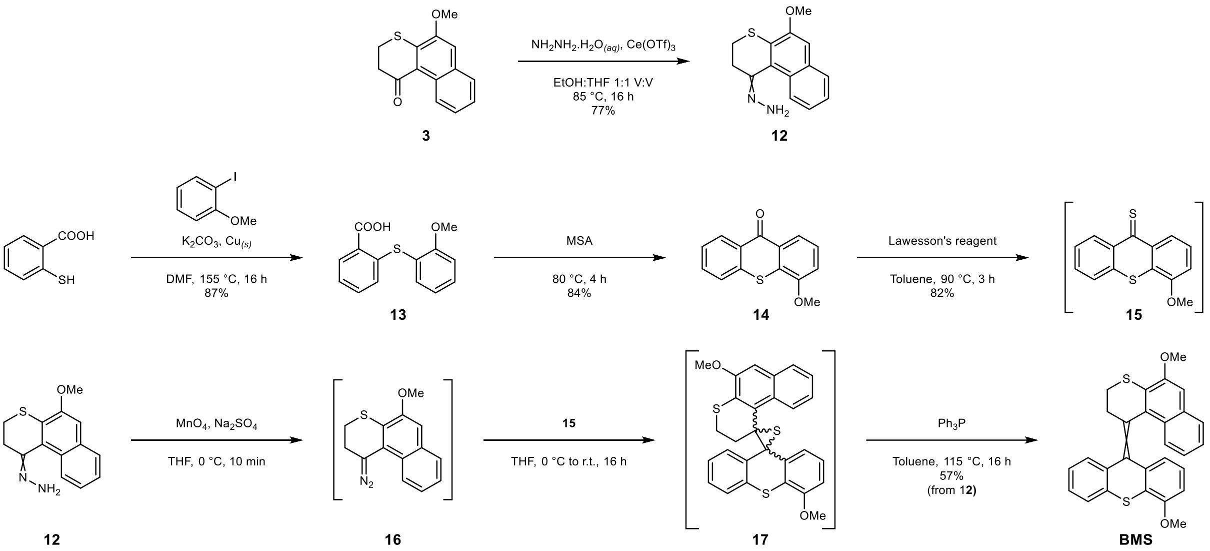

Supplementary Fig. 4 | Synthetic scheme of overcrowded alkene BMS. Schematic illustration of synthetic route of the control compound **BMS**.

1.2.2 Experimental procedures

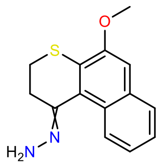

<u>(5-Methoxy-2,3-dihydro-1*H*-benzo[*f*]thiochromen-1-ylidene)hydrazine (**12**)</u>

Under a nitrogen atmosphere, in a 250 mL three-neck round-bottom flask, thiochomanone **3** (1.115 g, 4.518 mmol, 1.0 eq.) and cerium(III) trifluoromethanesulfonate (0.271 g, 0.415 mmol, 0.08 eq.) were dissolved in a mixture of EtOH:THF (1:1 V:V, 60 mL, 0.075 M). Then, an aqueous solution of hydrazine monohydrate (80 wt.%, 16.0 mL, 264 mmol, 60 eq.) was added to the solution, and the resulting heterogeneous mixture was heated at 85 °C and stirred overnight. After cooling to room temperature, the volatile solvents were evaporated under reduced pressure. To the resulting residue was added a saturated aqueous solution of $NaHCO_3$ (100 mL) and the product was extracted with EtOAc (3 x 100 mL). The combined organic phase was washed with brine (100 mL), dried with Na_2SO_4, and concentrated under vacuum to obtain a brown solid. The crude product was purified by flash

chromatography (SiO_2, *n*-Pent:EtOAc 1:1 V:V) to obtain the product as a yellow solid (0.907 g, 3.51 mmol, 77%, ratio *E*:*Z* 19:1). ^{1}H NMR (600 MHz, $CDCl_3$): δ = 8.59–8.55 (m, 1H), 7.69–7.65 (m, 1H), 7.40–7.32 (m, 2H), 7.04 (s, 1H), 5.53 (br s, 2H), 3.99 (s, 3H), 3.04 (ddd, J = 7.1, 5.8, 1.4 Hz, 2H), 2.99 (ddd, J = 7.0, 5.9, 1.4 Hz, 2H) ppm; ^{13}C NMR (151 MHz, $CDCl_3$): δ = 153.6, 145.8, 133.5, 131.2, 129.3, 127.4, 127.1, 126.9, 125.8, 124.5, 105.3, 56.1, 30.4, 26.9 ppm; IR (FT-IR): ν = 3416, 3310, 3168 (m, N–$H_{hydrazone}$), 3080 (w, C=$C_{arom.}$), 2966, 2926, 2872 (m, C–H_{alkane}), 1654 (m, C=$N_{hydrazone}$), 1586, 1578 (m, C=$C_{arom.}$) cm^{-1}; HRMS (ESI pos.): calculated for $C_{14}H_{14}N_2OS$ ($[M + H]^+$): 259.0900, found 259.0898.
Note: Due to the low ratio of *(Z)*-**12**, NMR characterizations has been performed only for *(E)*-**12**.

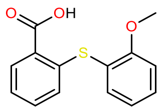

2-((2-Methoxyphenyl)thio)benzoic acid (**13**)

Under a nitrogen atmosphere, in a 250 mL three-neck round-bottom flask, thiosalicylic acid (3.780 g, 23.78 mmol, 1.0 eq.), 2-iodoanisole (3.3 mL, 25 mmol, 1.05 eq.), anhydrous potassium carbonate (5.990 g, 43.34 mmol, 1.75 eq.) and copper(0) powder (ca. 100 mesh, 89 mg, 1.401 mmol, 0.06 eq.) were placed in dry DMF (31 mL, 0.8 M) and the mixture was heated at reflux and stirred overnight. After cooling to room temperature, the mixture was diluted with water (150 mL). The resulting heterogeneous mixture was filtrated on a plug of celite and the filtrate was acidified with an aqueous solution of HCl (3 M, 100 mL) to precipitate the expected product. The solid was filtered and washed with cold water to provide a grey solid as a crude. The crude product was dissolved in a minimum amount of DCM, before adding *n*-Pent until precipitation of the product. The solid was filtrated and washed with *n*-Pent to obtain the purified product as a white powder (5.577 g, 21.42 mmol, 87%). ^{1}H NMR (600 MHz, DMSO-d_6): δ = 7.91 (dd, J = 7.8, 1.6 Hz, 1H), 7.51 (ddd, J = 8.4, 7.4, 1.8 Hz, 1H), 7.47 (dd, J = 7.5, 1.7 Hz, 1H), 7.31 (ddd, J = 8.4, 7.3, 1.6 Hz, 1H), 7.19–7.15 (m, 2H), 7.05 (dt, J = 7.5, 1.1 Hz, 1H), 6.61 (dd, J = 8.2, 1.1 Hz, 1H), 3.73 (s, 3H) ppm; ^{13}C NMR (151 MHz, DMSO-d_6): δ = 167.5, 159.9, 141.3, 137.0, 132.2, 131.8, 130.9, 127.1, 126.0, 124.2, 121.6, 119.1, 112.2, 55.8 ppm; IR (FT-IR): ν = 3250–2250 (br m, O–$H_{benzoic\ acid}$), 1673 (s, C=$O_{benzoic\ acid}$), 1584, 1558 (m, C=$C_{aromatic}$) cm^{-1}; HRMS (ESI pos.): calculated for $C_{14}H_{12}O_3SNa$ ($[M + Na]^+$): 283.0399, found 283.0395.

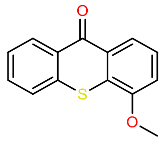

4-Methoxy-9*H*-thioxanthen-9-one (**14**)

Under a nitrogen atmosphere, in a 100 mL round-bottom flask, carboxylic acid **13** (1.016 g, 3.903 mmol, 1.0 eq.) was dissolved in methanesulfonic acid (20 mL, 0.2 M) and the mixture was heated at 80 °C and stirred for 4 h. The flask was cooled down at room temperature before pouring the solution on a mixture of water (100 mL) and ice (100 g). The product was extracted with DCM (3 x 100 mL) and the combined organic layer was washed with brine (150 mL), dried with $MgSO_4$, and filtered on a plug of silica. The filtrate was concentrated under vacuum to obtain the purified product as a grey solid (0.797 g, 3.29 mmol, 84%). ^{1}H NMR (600 MHz, $CDCl_3$): δ = 8.61 (dd, J = 8.1, 1.4 Hz, 1H), 8.26 (dd, J = 8.1, 1.1 Hz, 1H), 7.66–7.60 (m, 2H), 7.48 (ddd, J = 8.2, 6.8, 1.5 Hz, 1H), 7.45 (t, J = 8.0 Hz, 1H), 7.13 (dd, J = 8.0, 1.1 Hz, 1H), 4.04 (s, 3H) ppm; ^{13}C NMR (151 MHz, $CDCl_3$): δ = 180.2, 154.5, 137.5, 132.3, 130.4, 129.8, 129.1, 127.7, 126.9, 126.5, 126.1, 121.8, 112.4, 56.6 ppm; IR (FT-IR): ν = 3068, 3051 (w, C–$H_{aromatic}$), 3003, 2941, 2838 (w, C–H_{alkane}), 1629 (m, C=O_{ketone}), 1594, 1569 (m, C=$C_{aromatic}$) cm^{-1}; HRMS (ESI pos.): calculated for $C_{14}H_{11}O_2S$ ($[M + H]^+$): 243.0474, found 243.0474.

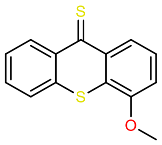

4-Methoxy-9*H*-thioxanthene-9-thione (**15**)

Under a nitrogen atmosphere, in a 100 mL three-neck round-bottom flask, thioxanthone **14** (0.507 g, 2.09 mmol, 1.0 eq.) and Lawesson's reagent (3.175 g, 7.850 mmol, 4.0 eq.) were placed in dry toluene (100 mL, 0.002 M) and the mixture was heated at 90 °C and stirred for 3 h. After cooling to room temperature, the toluene was removed by evaporation under vacuum to obtain a yellowish-green powder as a crude. The product was purified by column chromatography (SiO_2, *n*-Pent:EtOAc 19:1 V:V) to obtain a green powder as a pure compound (0.442 g, 1.71 mmol, 82%). ^{1}H NMR (600 MHz, $CDCl_3$): δ = 9.01 (dd, J = 8.5, 1.4 Hz, 1H), 8.67 (dd, J = 8.5, 1.1 Hz, 1H), 7.65 (dd, J = 8.1, 1.4 Hz, 1H), 7.61 (ddd, J = 8.1, 6.8, 1.4 Hz, 1H), 7.44 (ddd, J = 8.4, 6.8, 1.4 Hz, 1H), 7.39 (t, J = 8.1 Hz, 1H), 7.13 (dd, J = 7.9, 1.0 Hz, 1H), 4.05 (s, 3H) ppm; ^{13}C NMR (151 MHz, $CDCl_3$): δ = 211.0, 154.5, 138.6, 137.5, 133.4, 132.2, 131.7, 127.1, 126.8, 126.5, 125.5, 123.0, 111.4, 77.4, 77.2, 76.9, 56.7 ppm.
Note: This thioketone is stable under a nitrogen atmosphere for 12 h only. Therefore, it is advised to use it immediately after isolation. Because of low stability upon air exposure, only ^{1}H and ^{13}C NMR spectra have been performed for characterizations.

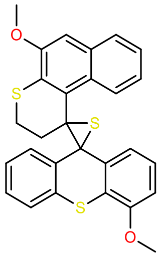

4'',5-Dimethoxy-2,3-dihydrodispiro[benzo[*f*]thiochromene-1,2'-thiirane-3',9''-thioxanthene] (**17**)

Under a nitrogen atmosphere, manganese(IV) dioxide (Fluka, 3.854 g, 44.33 mmol, 17 eq.) and anhydrous sodium sulfate (2.604 g, 18.33 mmol, 7.0 eq.) were placed in a dried Schlenk flask containing a stirring egg. The Schlenk flask was connected by a cannula to another dried Schlenk flask containing a stirring egg. The two Schlenk flasks were covered with aluminum foil and placed at 0 °C. Subsequently, hydrazone **12** (0.336 g, 1.301 mmol, 1.0 eq.) and thioketone **15** (0.350 g, 1.355 mmol, 1.05 eq.) were separately dissolved in cold dry THF (10 mL for each, 0.05 M). The cold solution of hydrazone **12** was added to the tube containing the manganese(IV) dioxide and the mixture was stirred at 0 °C for 10 min. Then, the dark pink resulting solution of diazo **16** was transfered in the other Schlenk flask (using a cannula and nitrogen flow, filtering solid with filter paper) and the cold solution of thioketone **15** was dropwise added to the solution. The resulting mixture was slowly warmed to room temperature and stirred overnight, still covered with aluminum foil. The crude episulfide **17** (0.998 g) was obtained after concentrating under vacuum and used in the next step without further purification.

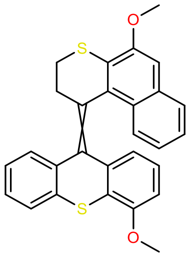

5-Methoxy-1-(4-methoxy-9*H*-thioxanthen-9-ylidene)-2,3-dihydro-1*H*-benzo[*f*]thiochromene (**BMS**)

Under a nitrogen atmosphere, in a 250 mL three-neck round-bottom flask, crude episulfide **17** (0.998 g) and triphenylphosphine (1.281 g, 4.883 mmol, 3.75 eq.) were dissolved in dry toluene (50 mL, 0.05 M), and the mixture was heated at reflux and stirred overnight. After cooling at room temperature, the toluene was removed by evaporation under vacuum. The mixture was isolated by flash chromatography (SiO_2, *n*-Pent:EtOAc 19:1 V:V) and the pure product was obtained as a white solid (0.336 g, 0.739 mmol, 57% from hydrazone **12**, ratio *E*:*Z* 3:1). ^{1}H NMR (600 MHz, CD_2Cl_2): [*(E)*-**BMS**] δ = 7.53 (d, *J* = 8.5 Hz, 1H), 7.50 (d, *J* = 8.0 Hz, 1H), 7.37 (t, *J* = 7.7 Hz, 1H), 7.33 (d, *J* = 7.9 Hz, 1H), 7.22 (d, *J* = 7.6 Hz, 1H), 7.09 (t, *J* = 7.2 Hz, 1H), 7.02 (s, 1H), 6.92 (d, *J* = 8.2 Hz, 1H), 6.90–6.86 (m, 1H), 6.80 (t, *J* = 7.7 Hz, 1H), 6.55 (d, *J* = 7.7 Hz, 1H), 6.48 (d, *J* = 7.1 Hz, 1H), 4.01 (s, 3H), 3.98 (s, 3H), 3.65–3.48 (m, 3H), 2.22–2.11 (m, 1H) ppm, [*(Z)*-**BMS**] δ = 7.65 (d, *J* = 7.7 Hz, 1H), 7.58 (d, *J* = 7.7 Hz, 1H), 7.53 (d, *J* = 8.5 Hz, 1H), 7.50 (d, *J* = 8.0 Hz, 1H), 7.39 (t, *J* = 7.4 Hz, 1 H), 7.31 (t, *J* = 7.5 Hz, 1H), 7.10 (t, *J* = 7.2 Hz, 1H), 7.02 (s, 1H), 6.90–6.86 (m, 1H), 6.47 (t, *J* = 7.1 Hz, 1H), 6.40 (d, *J* = 8.1 Hz, 1H), 6.20 (d, *J* = 7.7 Hz, 1H), 4.01 (s, 3H), 3.80 (s, 3H), 3.65–3.48 (m, 3H), 2.22–2.11 (m, 1H) ppm; ^{13}C NMR (151 MHz, CD_2Cl_2): [*(E)*-**BMS**] δ = 156.2, 154.3, 139.6, 138.6, 137.0, 136.0, 134.4, 133.8, 132.7, 132.3, 129.4, 127.1, 126.9, 126.7, 126.6, 125.7, 125.1, 124.6, 123.6, 123.4, 120.1, 109.2, 104.9, 56.5, 56.2, 30.2, 29.0 ppm, [*(Z)*-**BMS**] δ = 155.6, 152.8, 136.2, 136.1, 135.8, 132.8, 132.3, 128.0, 127.9, 127.8, 127.6, 127.3, 126.7, 126.6, 126.2, 125.2, 124.7, 124.5, 124.4, 123.4, 122.1, 108.4, 104.9, 56.4, 56.2, 30.2, 29.0 ppm; IR (FT-IR): ν = 3060 (w, C–$H_{aromatic}$), 3010, 2924, 2842 (w, C–H_{alkane}), 1592, 1560 (m, C=$C_{aromatic}$) cm^{-1}; HRMS (ESI pos.): calculated for $C_{28}H_{23}O_2S_2$ ($[M + H]^+$): 455.1134, found 455.1130.
Note: On the ^{1}H NMR spectrum (see Supplementary Fig. 58), both isomers are present. The integrations have been done for *(E)*-**BMS** only.

2. Supplementary Figures:

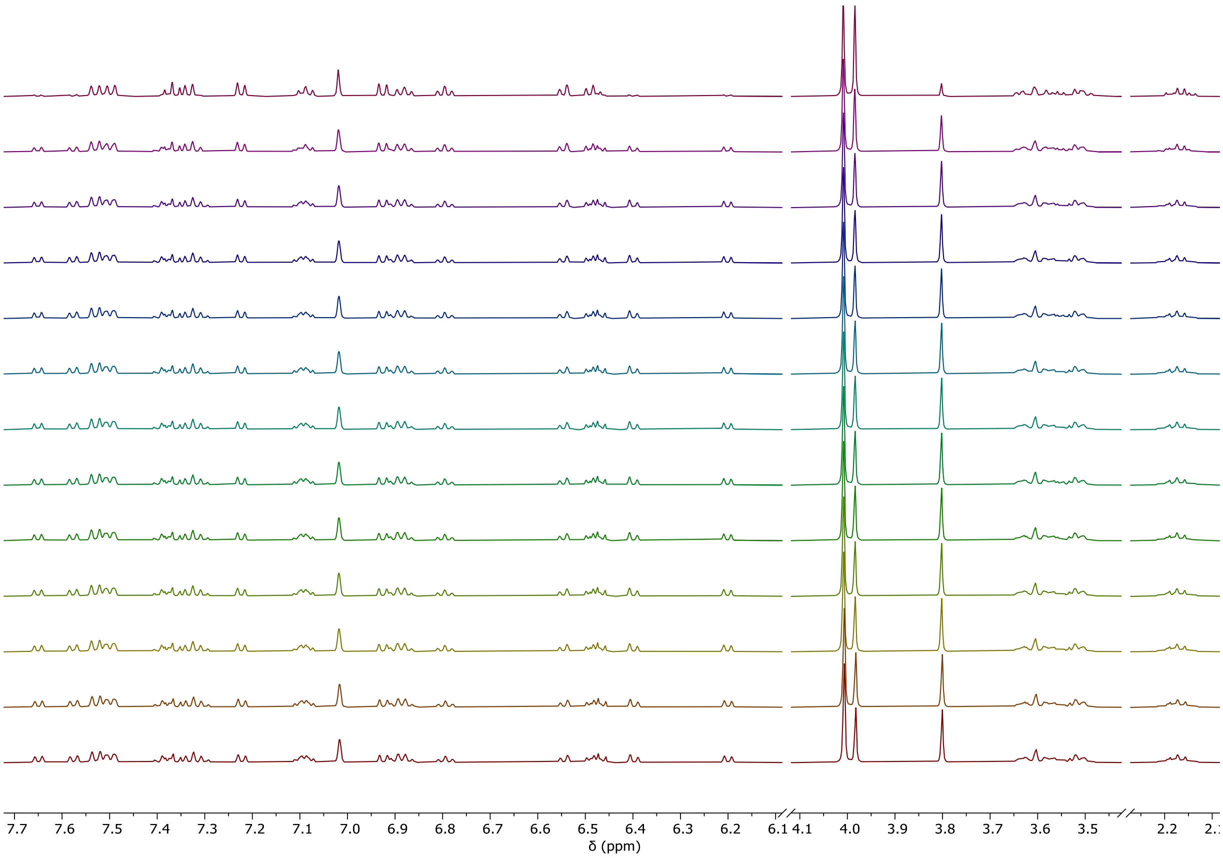

Supplementary Fig. 5 | Stacked ^{1}H NMR spectra of the photoisomerization process of BMS. Photoisomerization observations of *(E)*-enriched mixture of *(E/Z)*-**BMS** during 1 h of photoirradiation (from top to bottom, from 0 to 60 min every 5 min of irradiation recorded) (500 MHz, λ_{irr} = 365 nm, ratio *E*:*Z* 89:11, 4.0 mM in CD_2Cl_2, 25 °C).

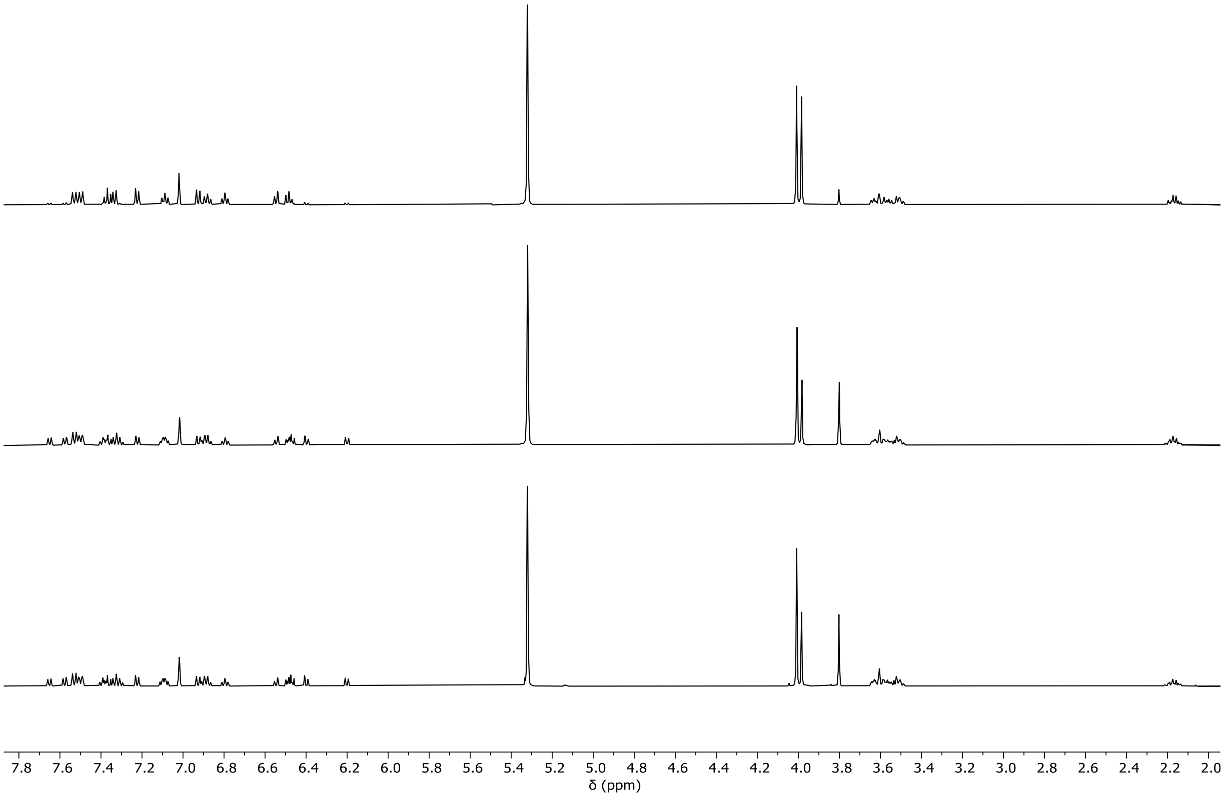

Supplementary Fig. 6 | Thermal stability of BMS after photoirradiation. ^{1}H NMR spectra of an *(E)*-enriched mixture of *(E/Z)*-**BMS** before photoirradiation (top), photostationnary state at 365 nm (middle), and after aging the irradiated solution during 3 d at room temperature (bottom) (500 MHz, λ_{irr} = 365 nm, ratio *E*:*Z* 89:11, 4.0 mM in CD_2Cl_2, 25 °C).

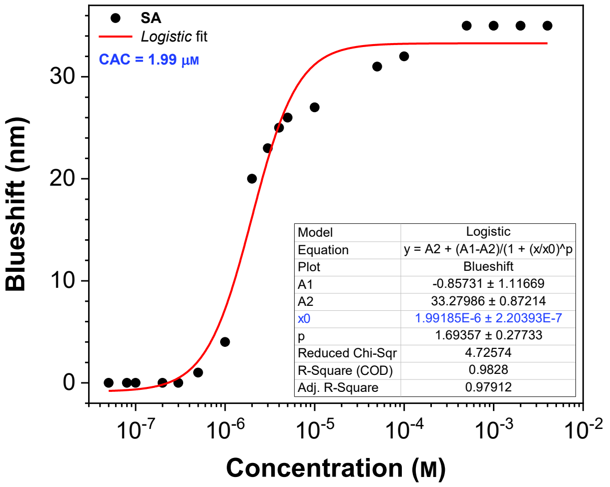

Supplementary Fig. 7 | Nile Red Fluorescent Assay (NRFA) of SA. Determination of the CAC of **SA** in double-deionized water, with a concentration range from 50 nM to 4.0 mM.

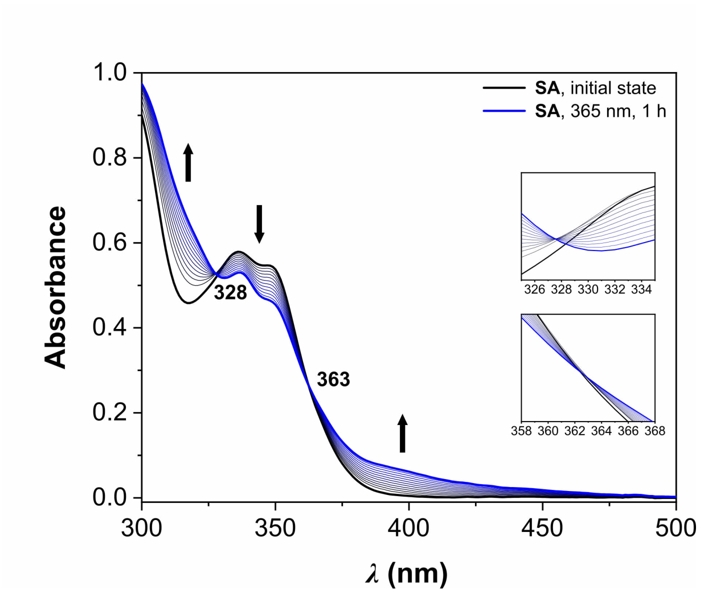

Supplementary Fig. 8 | Photoirradiation of SA sodium salt in aqueous media. UV-Vis spectra of irradiated sample of **SA** sodium salt in double-deionized water before and after irradiation with 365 nm light for 1 h (70 μM, 1.0 A, 25 °C).

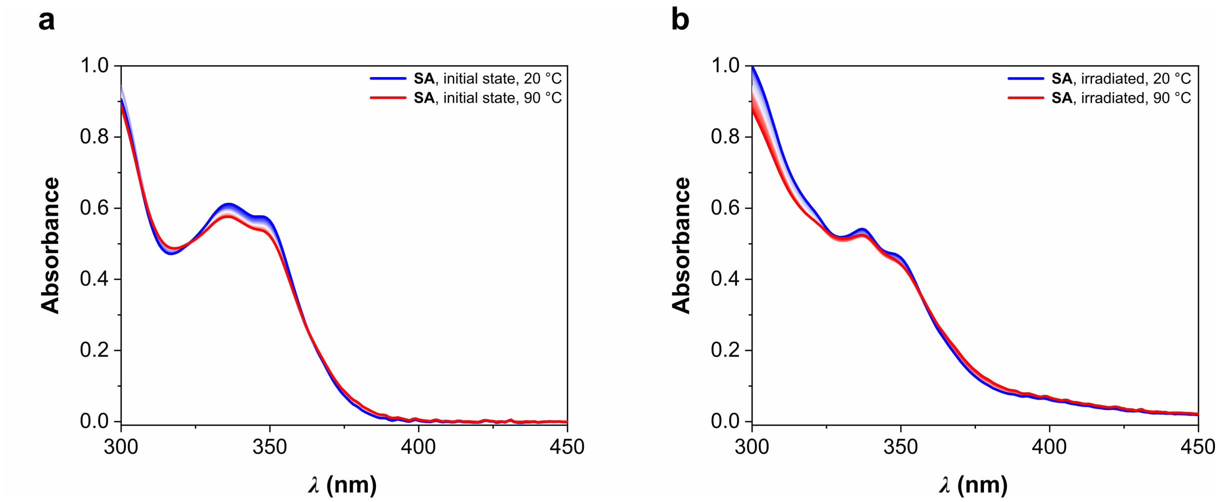

Supplementary Fig. 9 | UV-Vis spectra of SA sodium salt solution (70 μM) with *in-situ* heating from 20 °C to 90 °C (0.5 °C.min^{-1}). a SA sodium salt solution without irradiation was heated from 20 °C to 90 °C. **b SA** sodium salt solution upon 365 nm light irradiation for 1 h was heated from 20 °C to 90 °C.

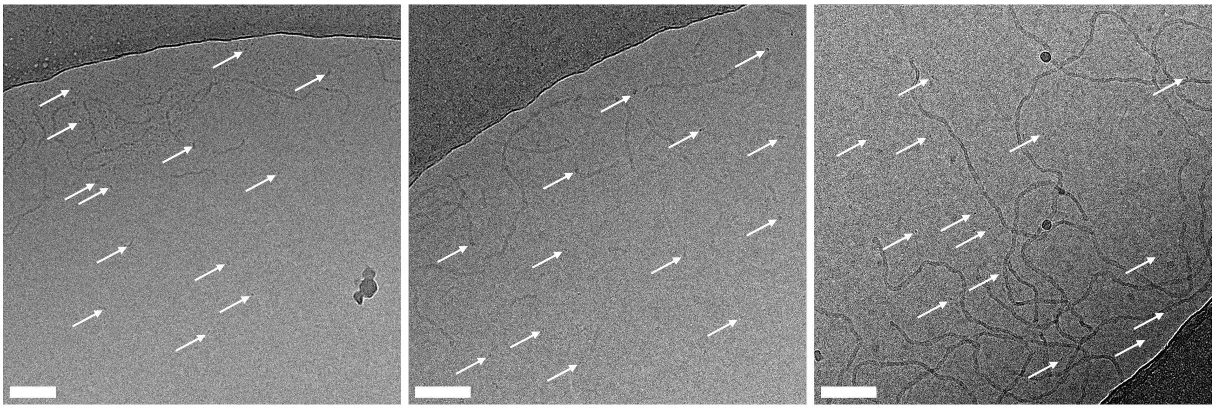

Supplementary Fig. 10 | Cryo-TEM images of a sodium salt of SA (5.5 mM) in double-deionized water before irradiation. A mixture of worm-like micelles and small micelles is observed. The micelles are indicated by white arrows. Not all micelles are indicated. Scale bar set for 100 nm.

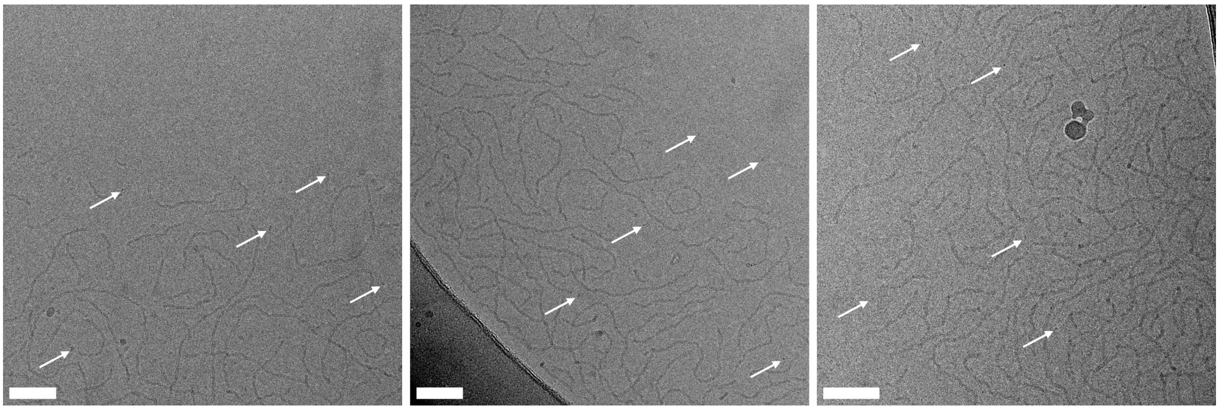

Supplementary Fig. 11 | Cryo-TEM images of a sodium salt of SA (5.5 mM) in double-deionized water after irradiation with 365 nm light for 1 h. Upon 365 nm light irradiation, a higher proportion of worm-line micelles is observed, indicating an assembly transformation from small micelles to worm-like micelles triggered by UV light. Micelles are indicated by white arrows. Not all micelles are indicated. Scale bar set for 100 nm.

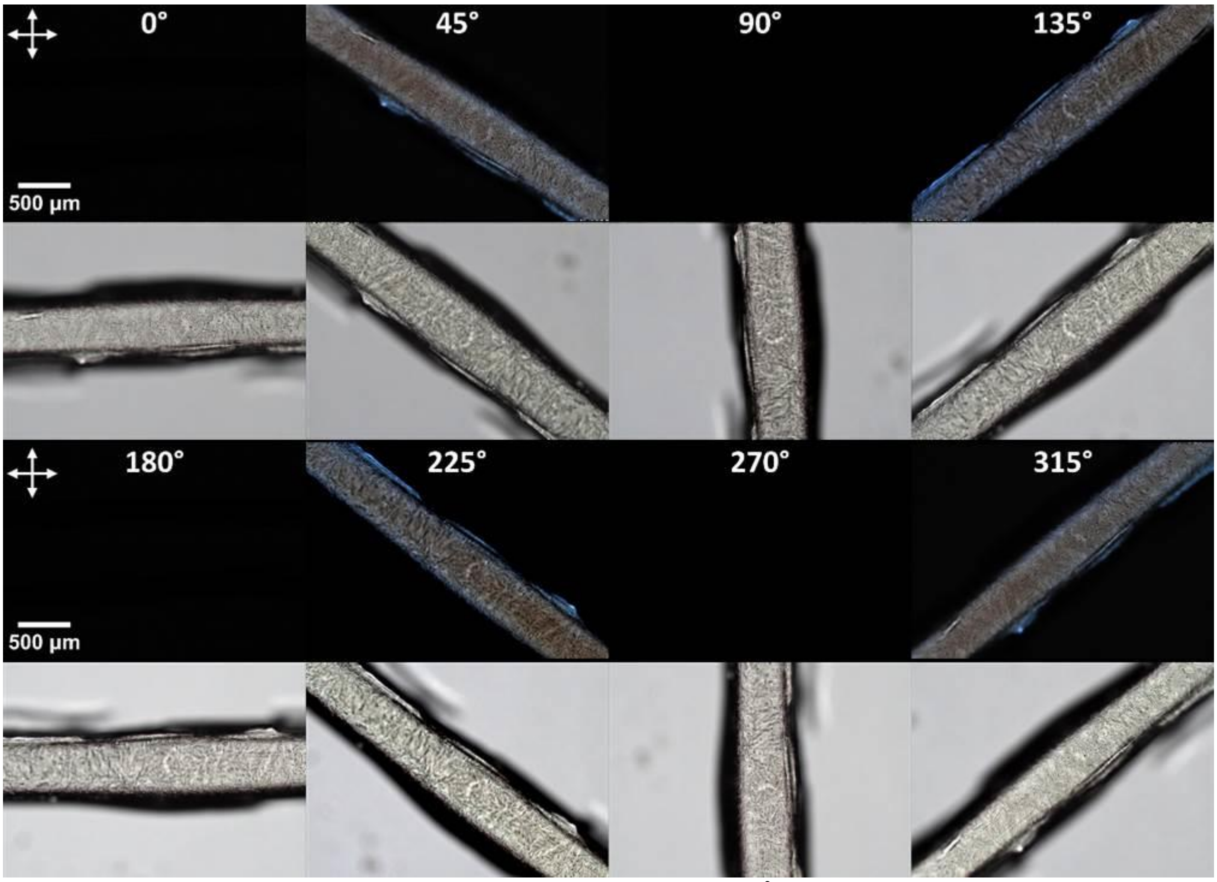

Supplementary Fig. 12 | Optical microscopic images of an SA-Mg^{2+} artificial muscle composed of SA (60 mM) prepared from an aqueous solution of $MgCl_2$ (150 mM) under crossed polarizers. The POM and OM images of the artificial muscle were tilted at 0°, 45°, 90°, 135°, 180°, 225°, 270° and 315° relative to the transmission axis of the analyzer. Scale bar for all panels.

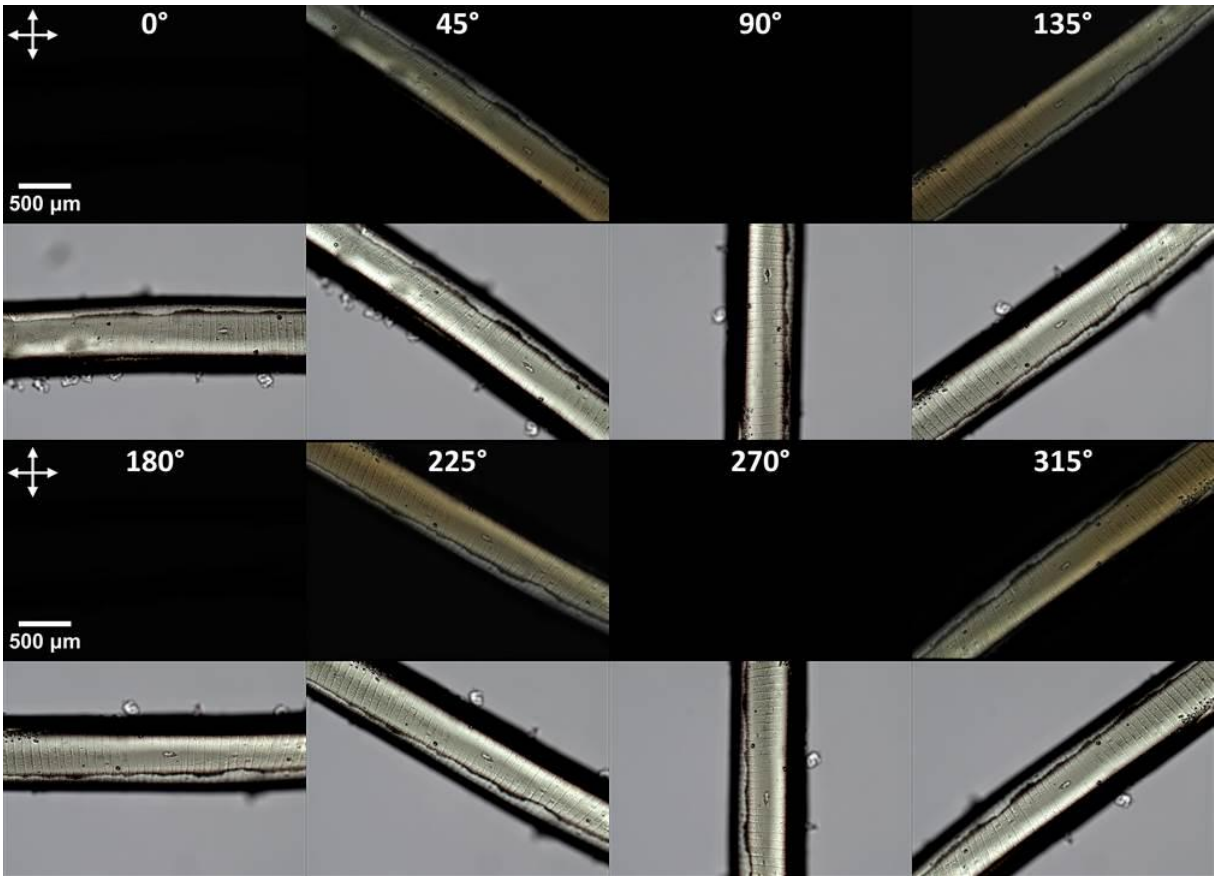

Supplementary Fig. 13 | Optical microscopic images of an SA-Ca^{2+} artificial muscle composed of SA (60 mM) prepared from an aqueous solution of $CaCl_2$ (150 mM) under crossed polarizers. The POM and OM images of the string were tilted at 0°, 45°, 90°, 135°, 180°, 225°, 270° and 315° relative to the transmission axis of the analyzer. Scale bar for all panels.

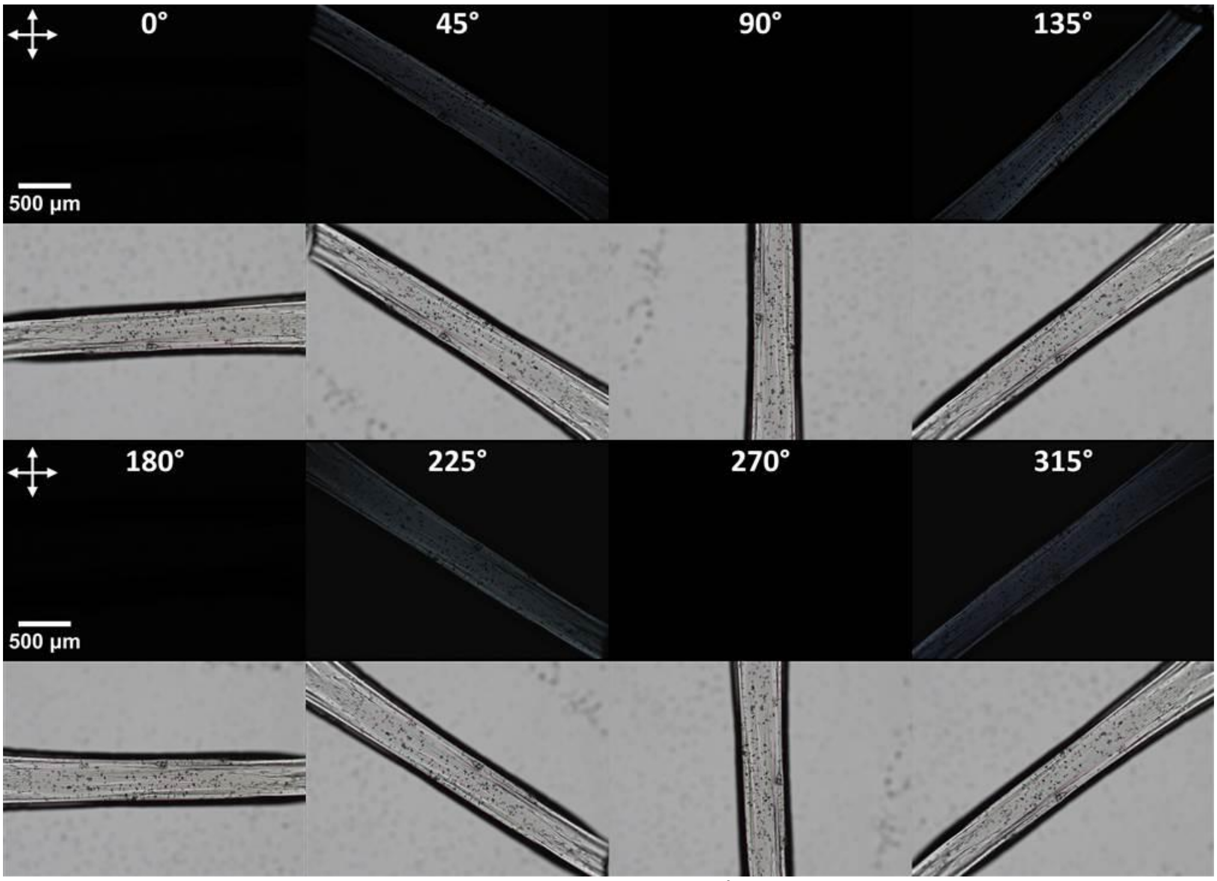

Supplementary Fig. 14 | Optical microscopic images of an SA-Sr^{2+} artificial muscle composed of SA (60 mM) prepared from an aqueous solution of $SrCl_2$ (150 mM) under crossed polarizers. The POM and OM images of the string were tilted at 0°, 45°, 90°, 135°, 180°, 225°, 270° and 315° relative to the transmission axis of the analyzer. Scale bar for all panels.

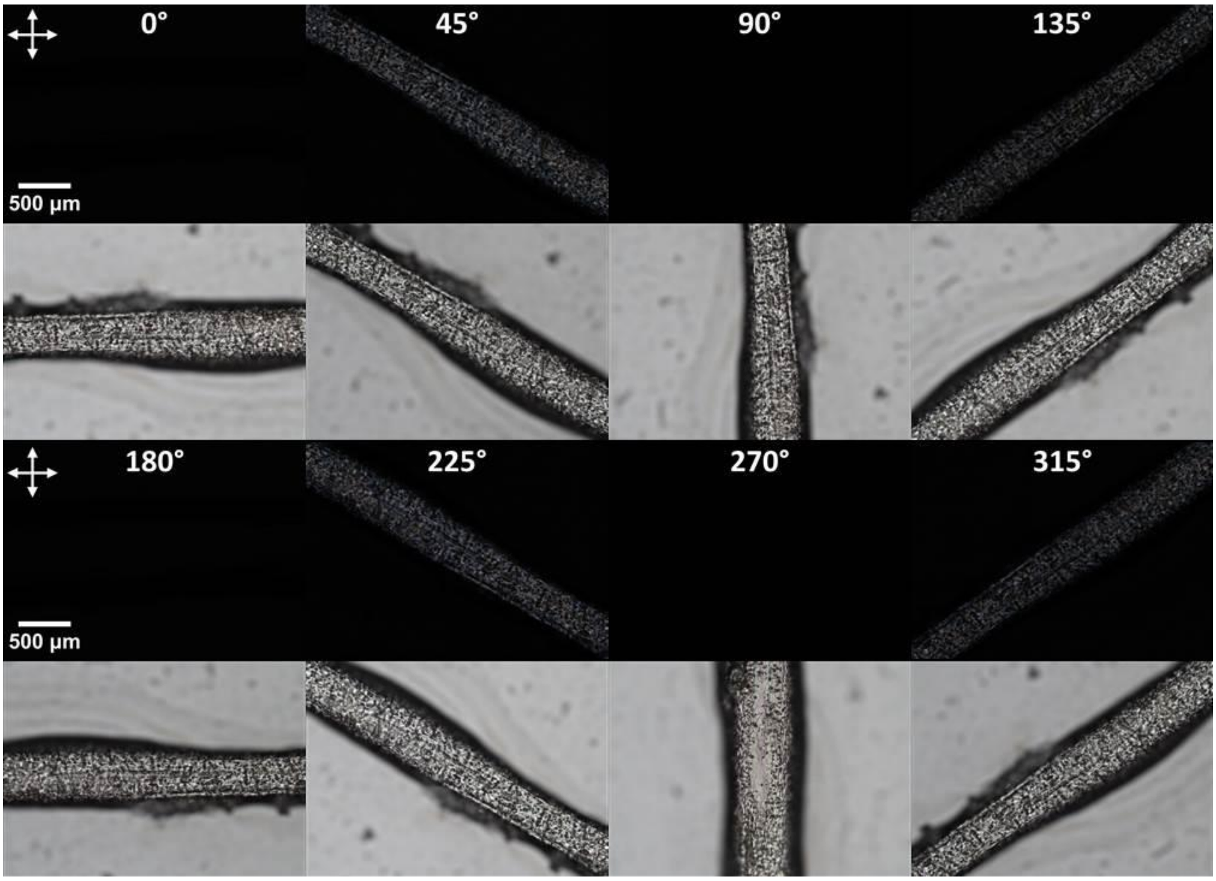

Supplementary Fig. 15 | Optical microscopic images of an SA-Ba^{2+} artificial muscle composed of SA (60 mM) prepared from an aqueous solution of $BaCl_2$ (150 mM) under crossed polarizers. The POM and OM images of the string were tilted at 0°, 45°, 90°, 135°, 180°, 225°, 270° and 315° relative to the transmission axis of the analyzer. Scale bar for all panels.

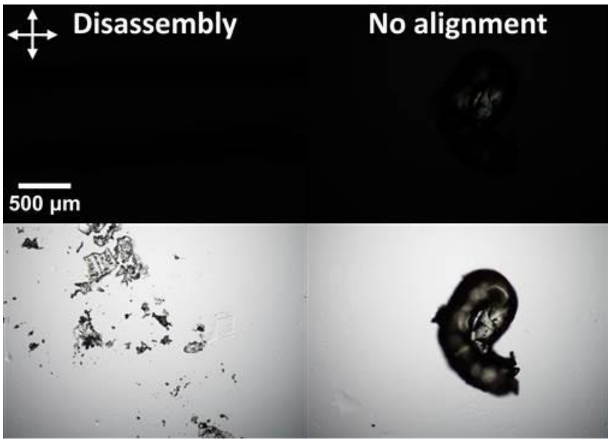

Supplementary Fig. 16 | Optical microscopic images of an extruded solution composed of SA (60 mM) into an aqueous solution of $BeCl_2$ (150 mM) under crossed polarizers. The POM and OM images of a disassembled string (on the left) and a curled macroscopic string (on the right) composed of **SA**. Scale bar for all panels.

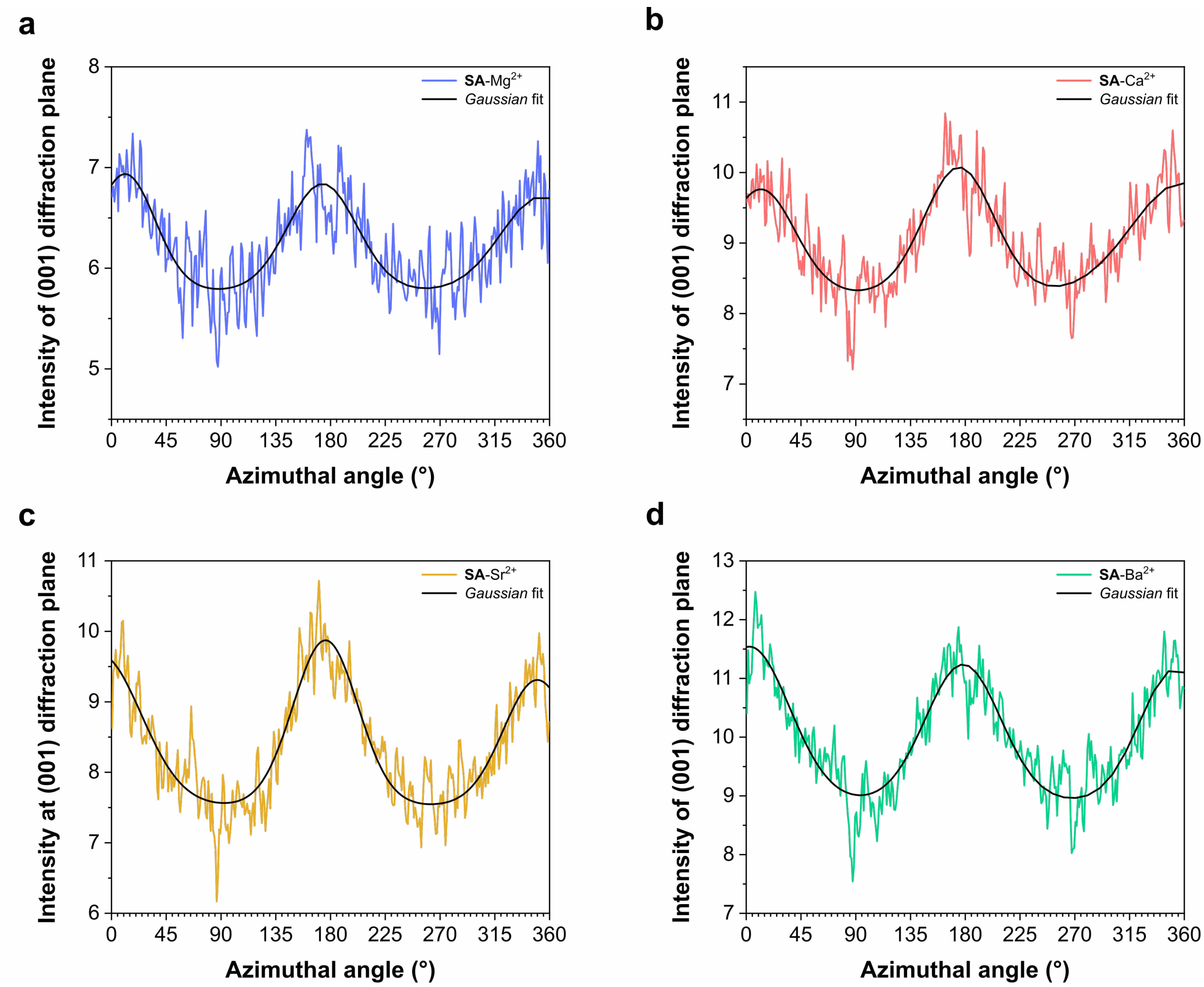

Supplementary Fig. 17 | Azimuthal intensity profiles for the (001) diffraction signal from the 2D SAXS patterns of SA artificial muscles. The artificial muscles were composed of **SA** (60 mM) prepared from **a** $MgCl_2$, **b** $CaCl_2$, **c** $SrCl_2$, and **d** $BaCl_2$ (150 mM) using the shear-flow method. The fitted curves provide the full-width half maximum (FWHM) related to the local alignment in the **SA** artificial muscles.

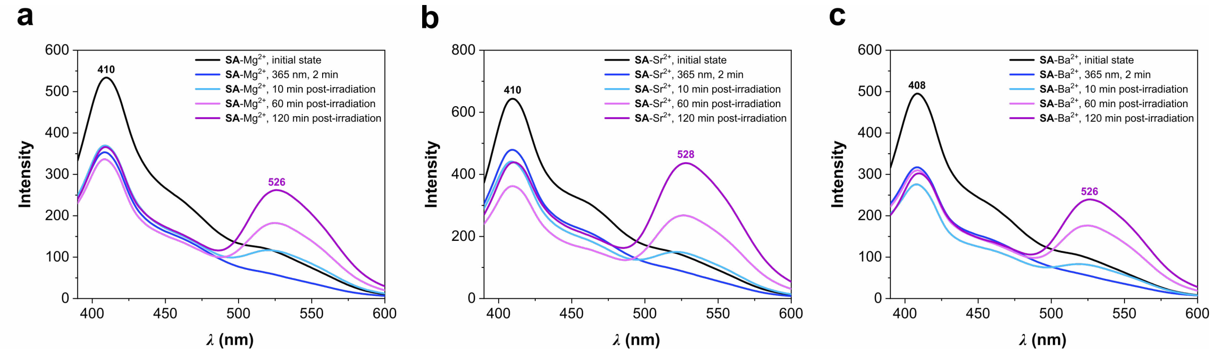

Supplementary Fig. 18 | Fluorescence spectra of SA-M^{2+} Artificial Muscles. Fluorescence spectral changes of an artificial muscle during the 365 nm light irradiation and subsequent aging process: **a SA**-Mg^{2+}, **b SA**-Sr^{2+} and **c SA**-Ba^{2+} (λ^{ex} = 365 nm, 25 °C). The artificial muscles were composed of **SA** (60 mM) immersed in the corresponding metallic chloride aqueous solutions (150 mM).

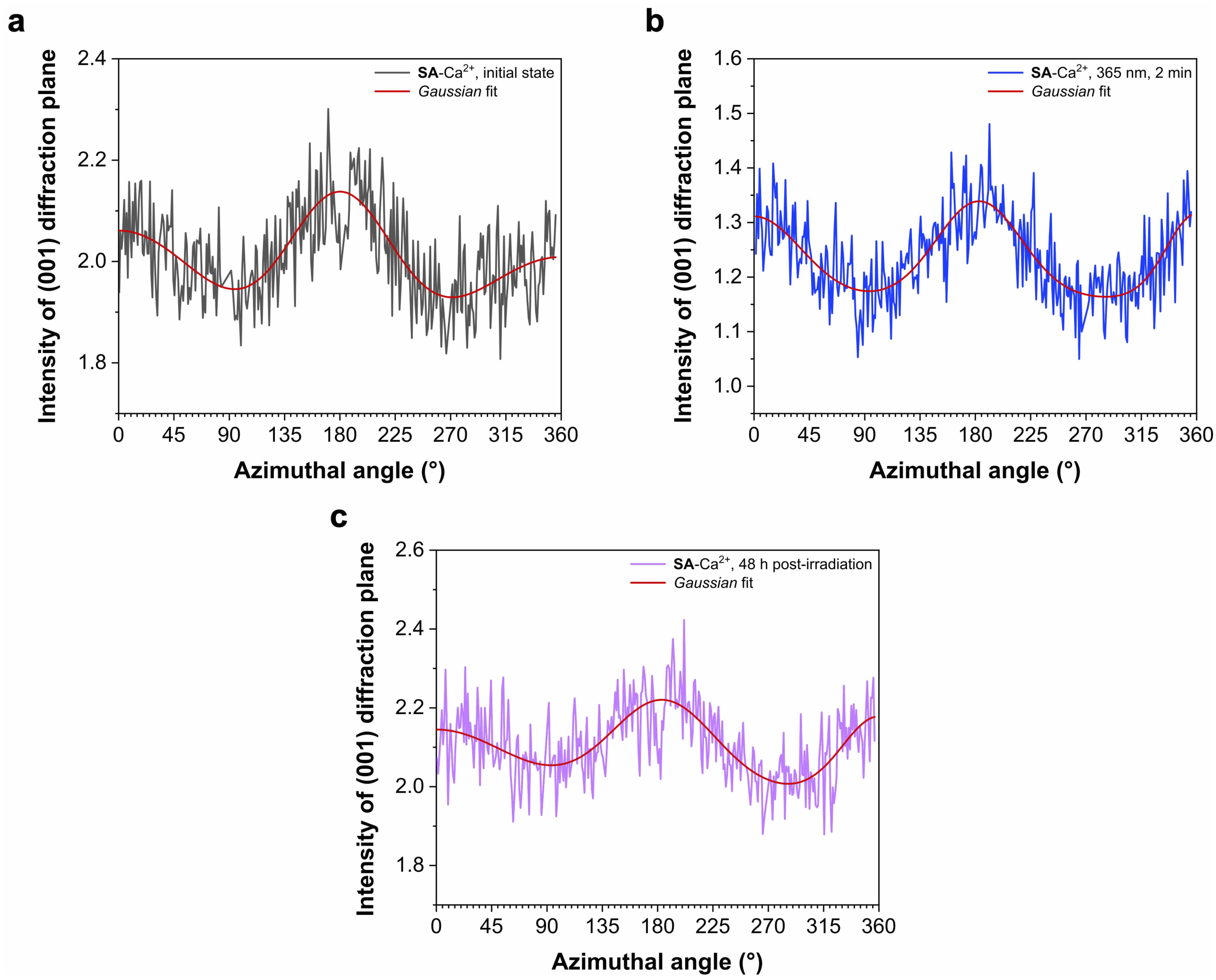

Supplementary Fig. 19 | Azimuthal intensity profiles for the (001) diffraction signal from the 2D SAXS patterns of an SA-Ca^{2+} artificial muscle. a The **SA**-Ca^{2+} artificial muscle was **b** exposed to 365 nm light for 2 min and then **c** aging for 48 h in the dark at room temperature. The fitted curves provide the full-width half maximum (FWHM) related to the local alignment of the **SA**-Ca^{2+} artificial muscle. The **SA**-Ca^{2+} artificial muscle (60 mM) was immersed in a calcium chloride solution (150 mM) during all measurements.

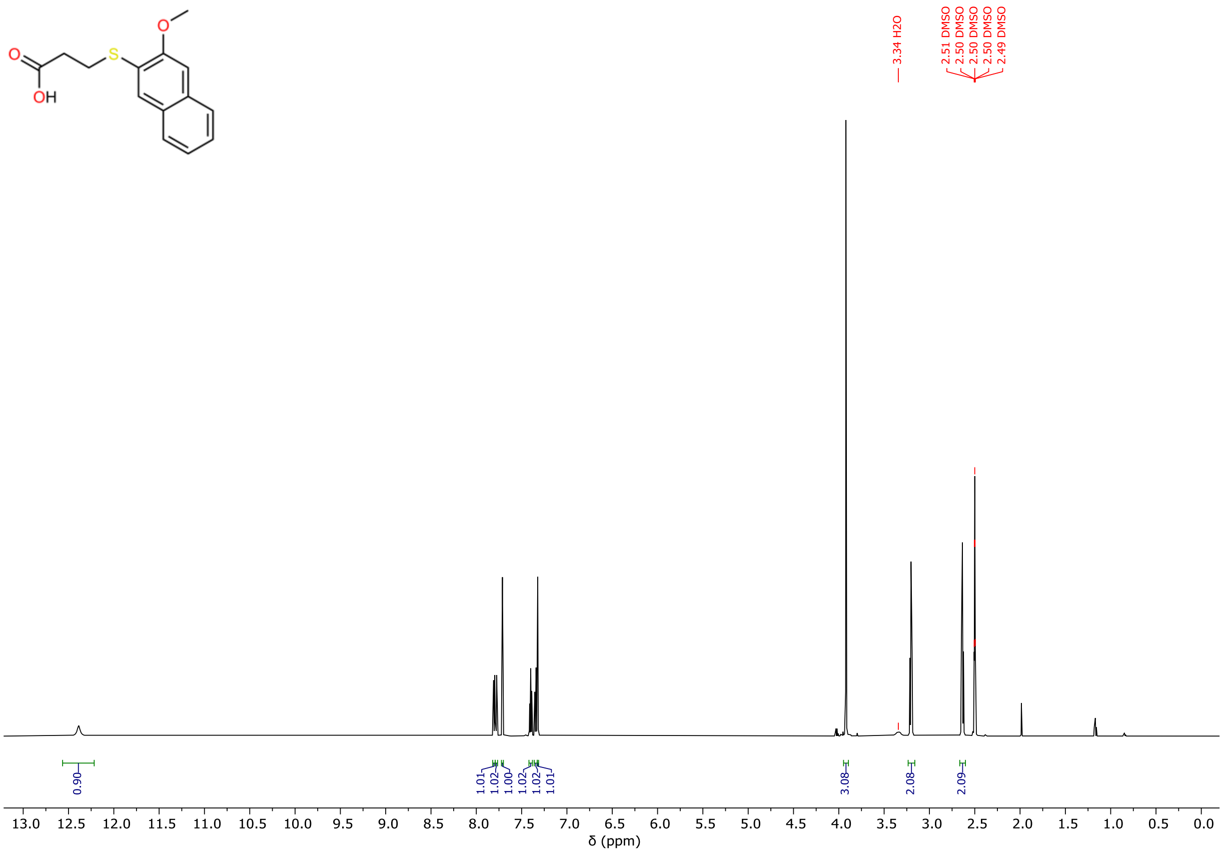

Supplementary Fig. 20 | ^{1}H NMR spectrum of carboxylic acid **2** (600 MHz, DMSO-d_6, 25 °C).

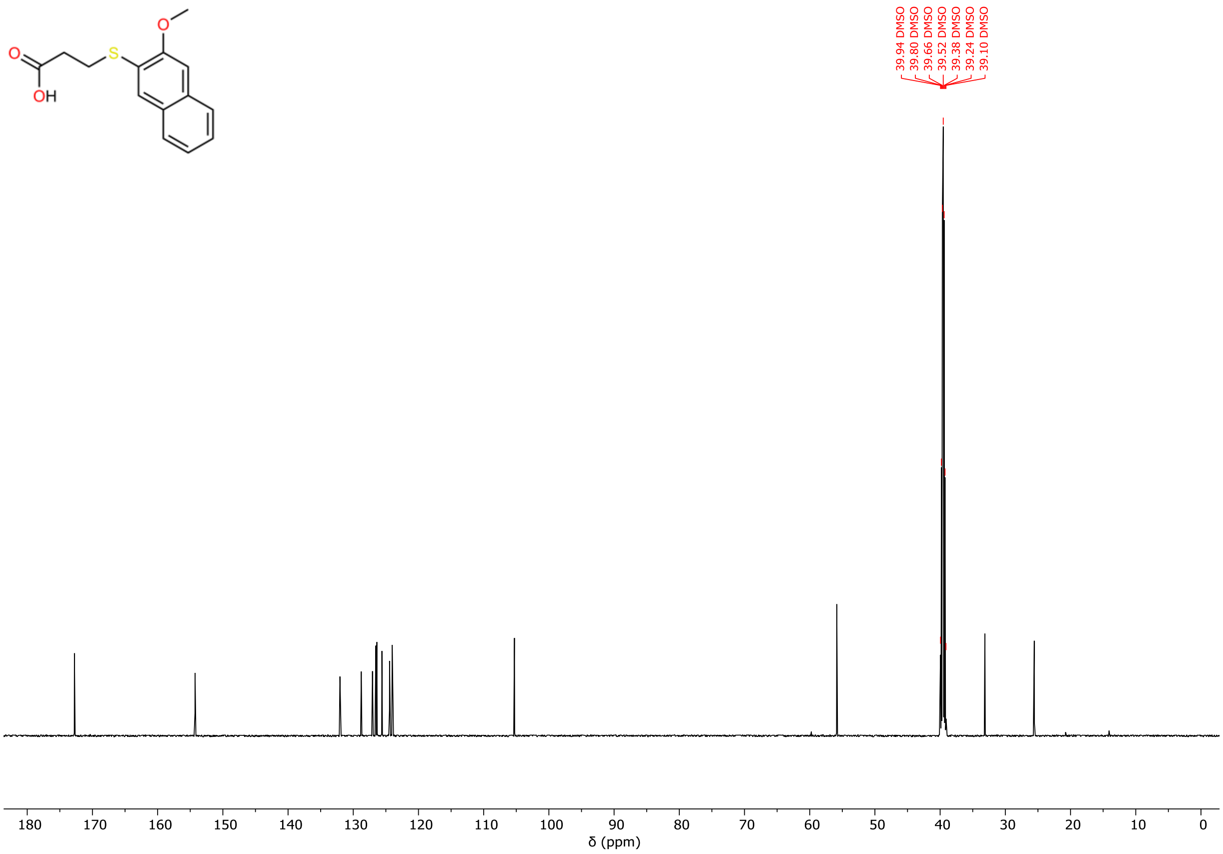

Supplementary Fig. 21 | ^{13}C NMR spectrum of carboxylic acid **2** (151 MHz, DMSO-d_6, 25 °C).

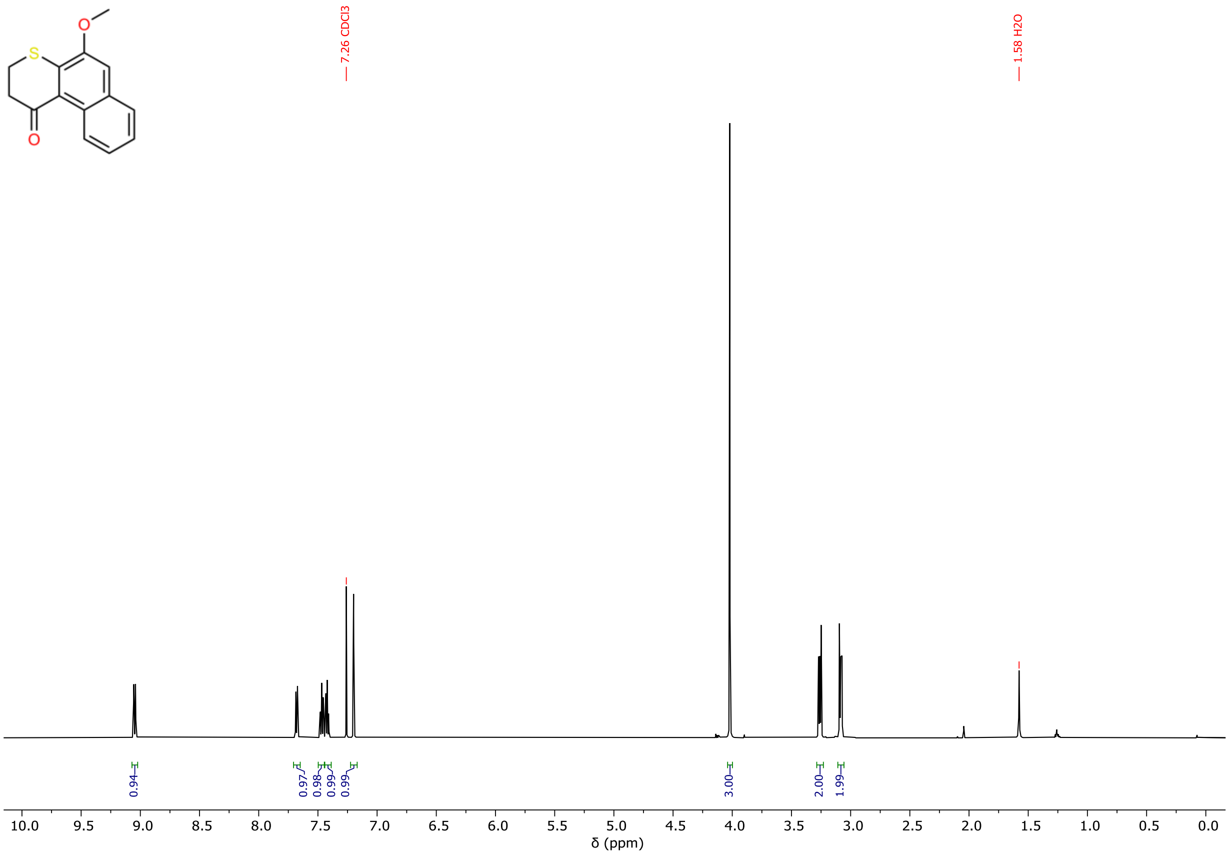

Supplementary Fig. 22 | ^{1}H NMR spectrum of thiochromanone **3** (600 MHz, $CDCl_3$, 25 °C).

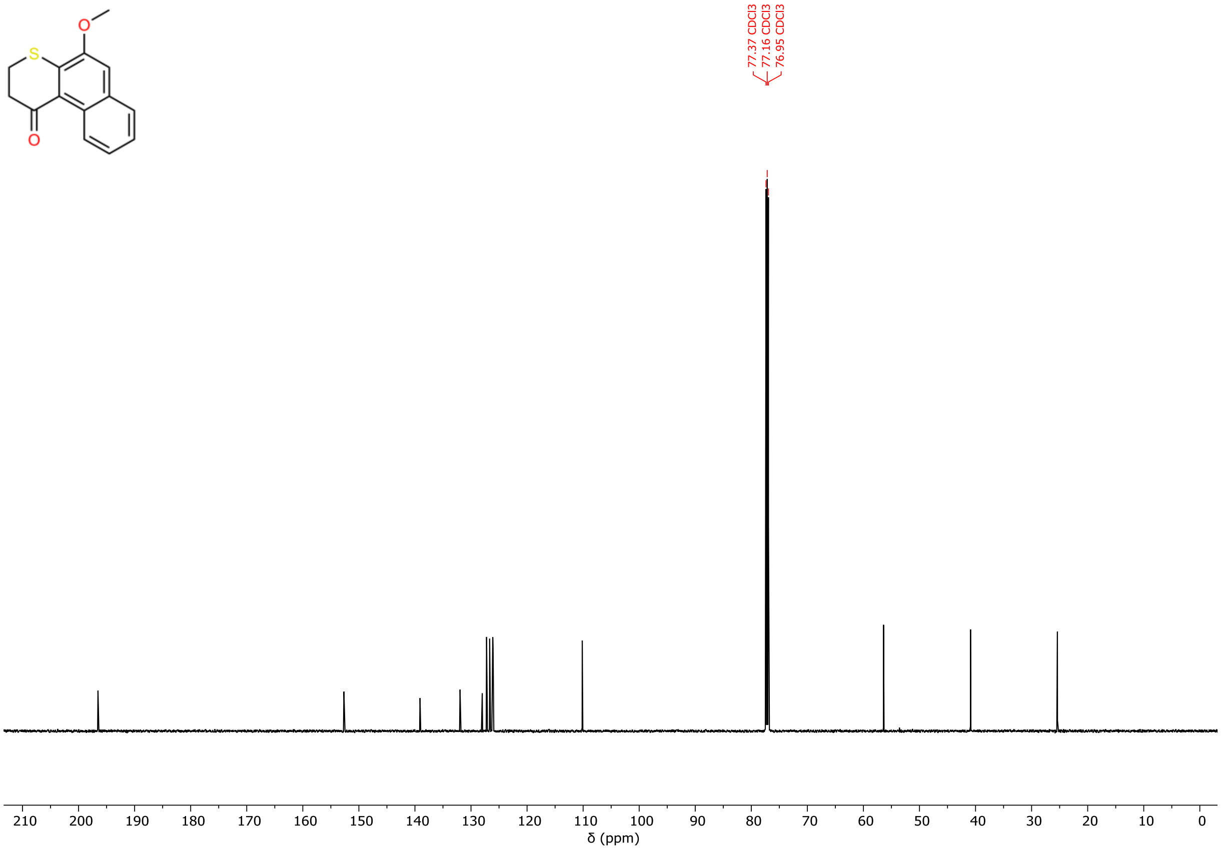

Supplementary Fig. 23 | ^{13}C NMR spectrum of thiochromanone **3** (151 MHz, $CDCl_3$, 25 °C).

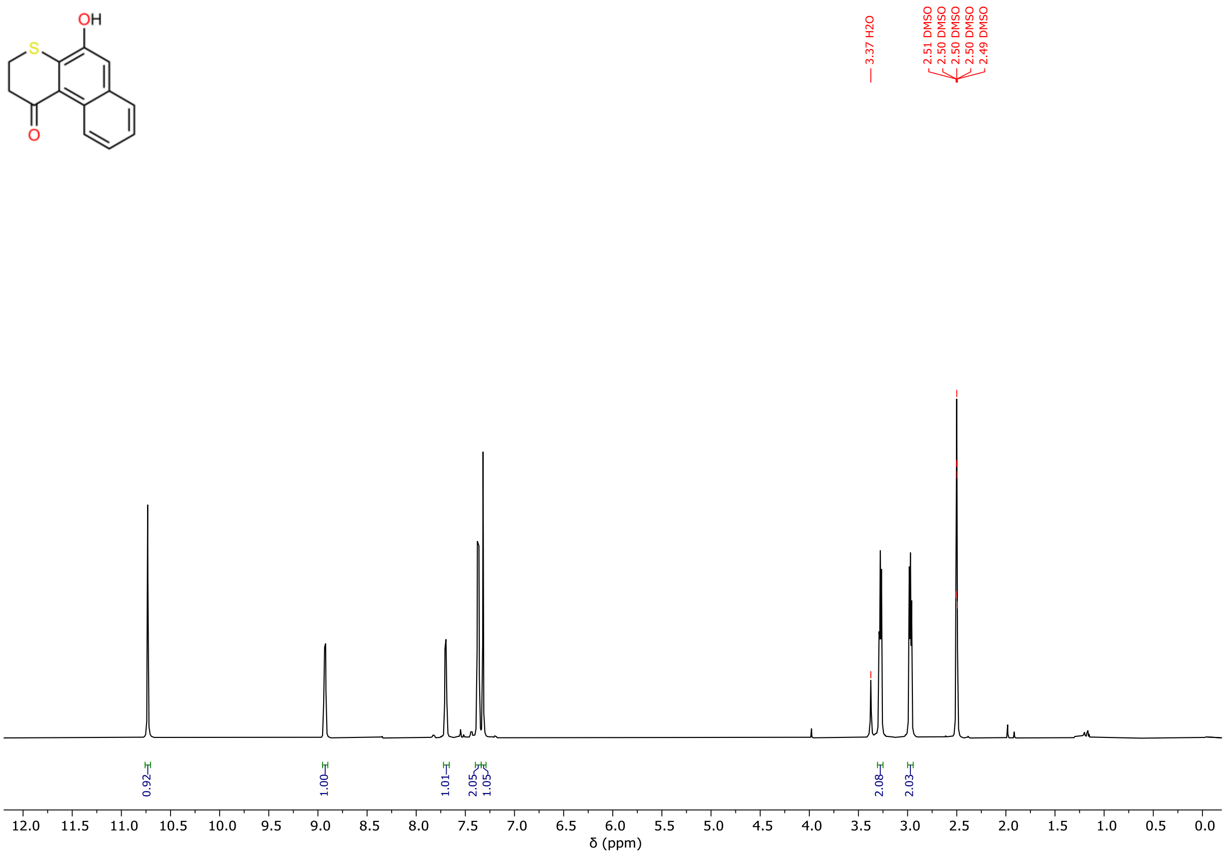

Supplementary Fig. 24 | ^{1}H NMR spectrum of phenol **4** (600 MHz, DMSO-d_6, 25 °C).

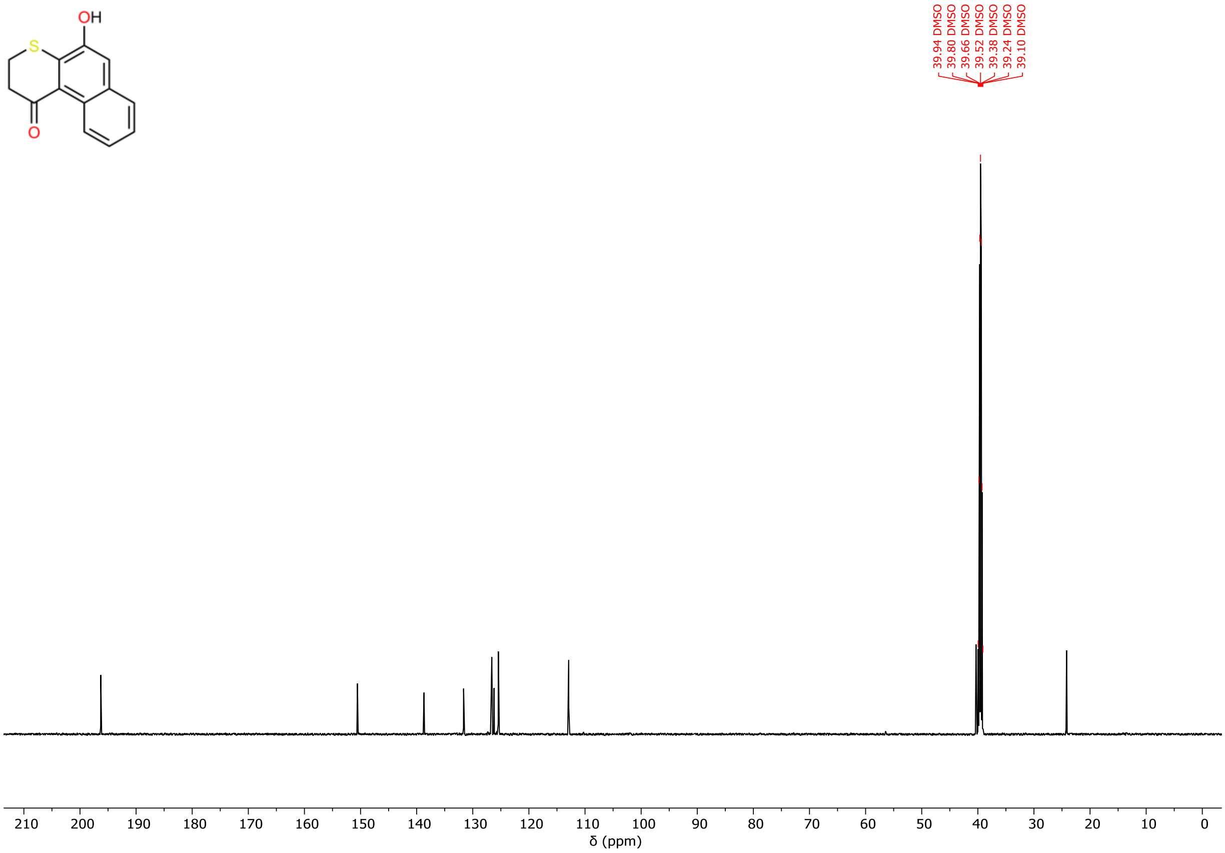

Supplementary Fig. 25 | ^{13}C NMR spectrum of phenol **4** (151 MHz, DMSO-d_6, 25 °C).

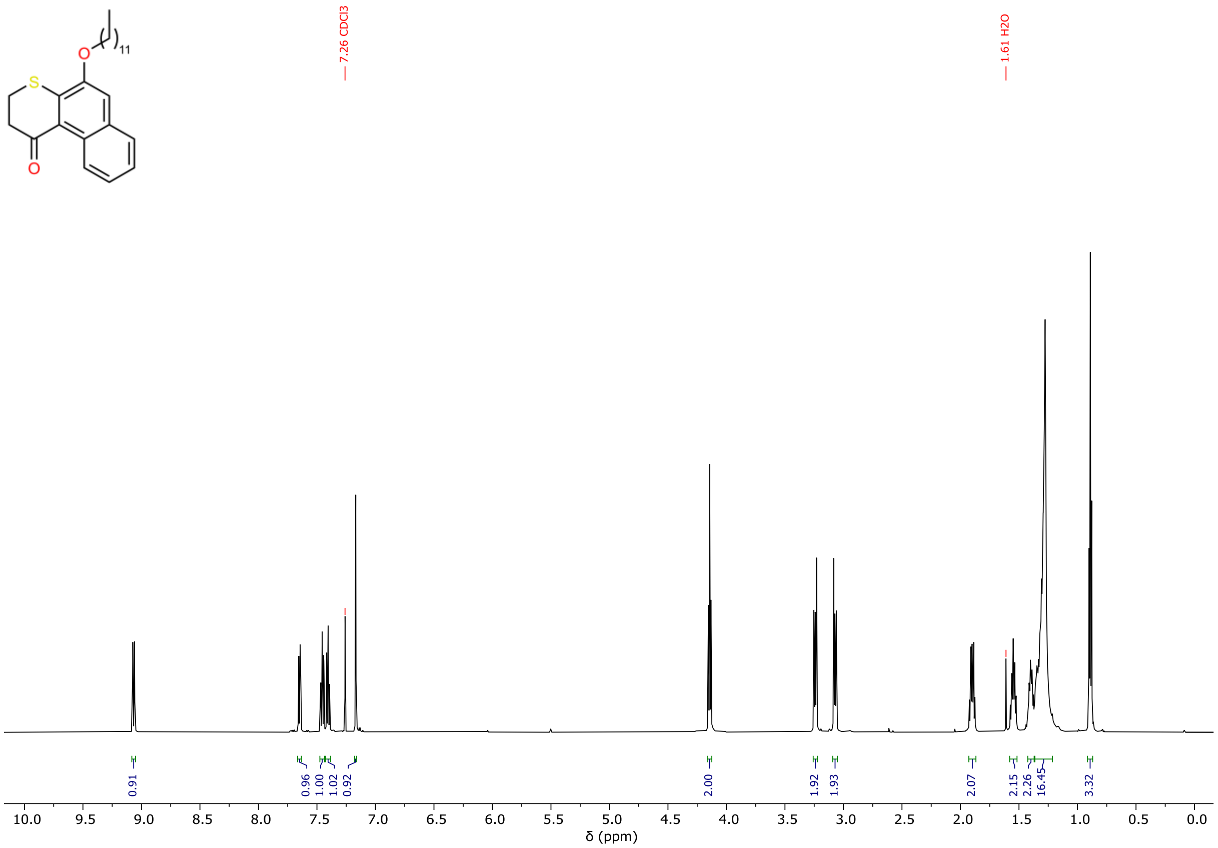

Supplementary Fig. 26 | ^{1}H NMR spectrum of thiochromanone **5** (600 MHz, $CDCl_3$, 25 °C).

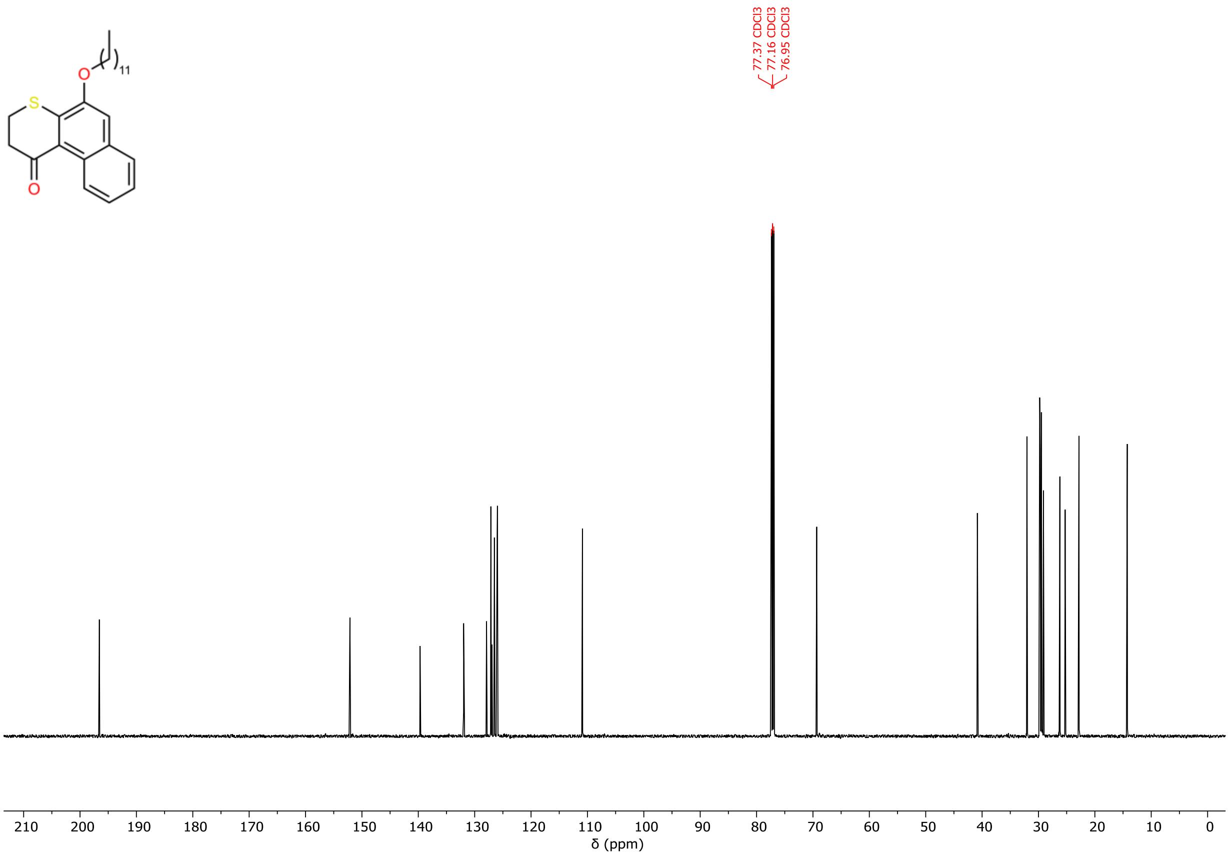

Supplementary Fig. 27 | ^{13}C NMR spectrum of thiochromanone **5** (151 MHz, $CDCl_3$, 25 °C).

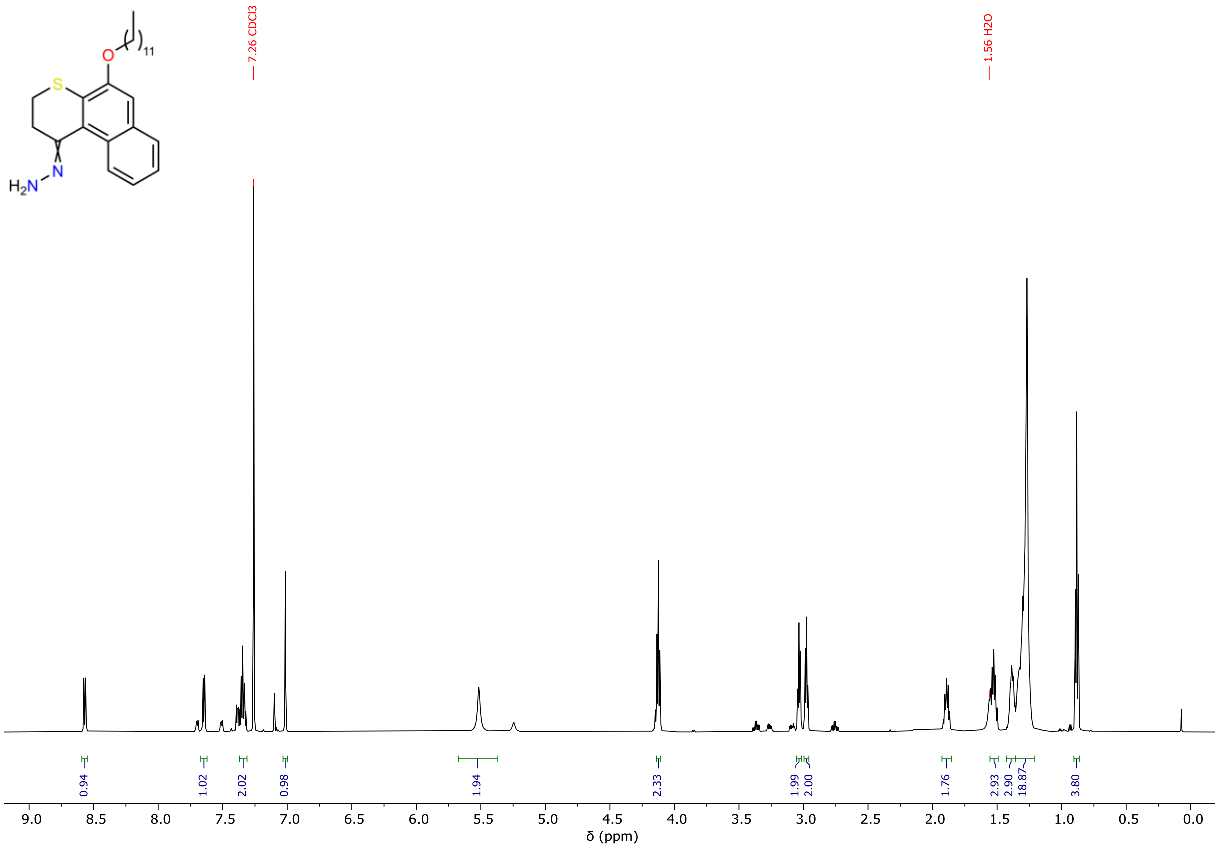

Supplementary Fig. 28 | ^{1}H NMR spectrum of hydrazone **6** (600 MHz, $CDCl_3$, 25 °C).

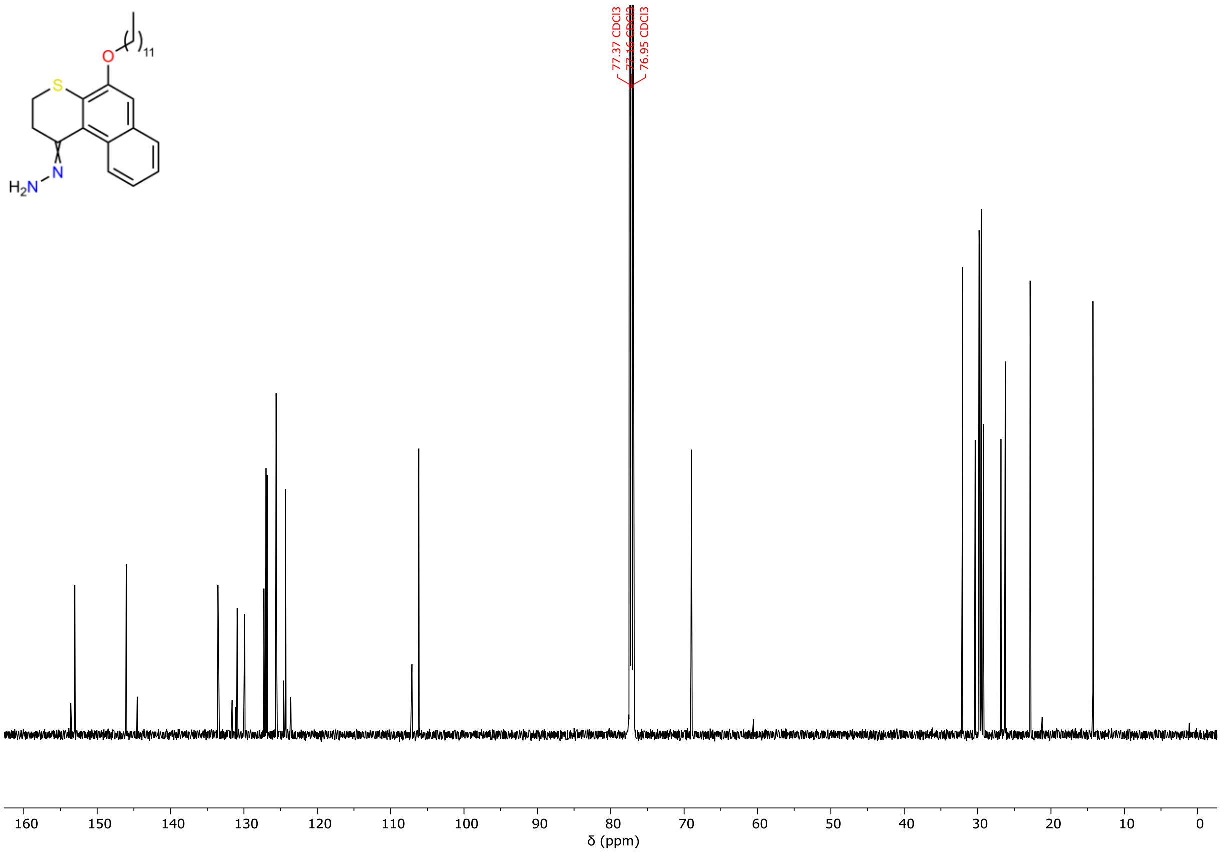

Supplementary Fig. 29 | ^{13}C NMR spectrum of hydrazone **6** (151 MHz, $CDCl_3$, 25 °C).

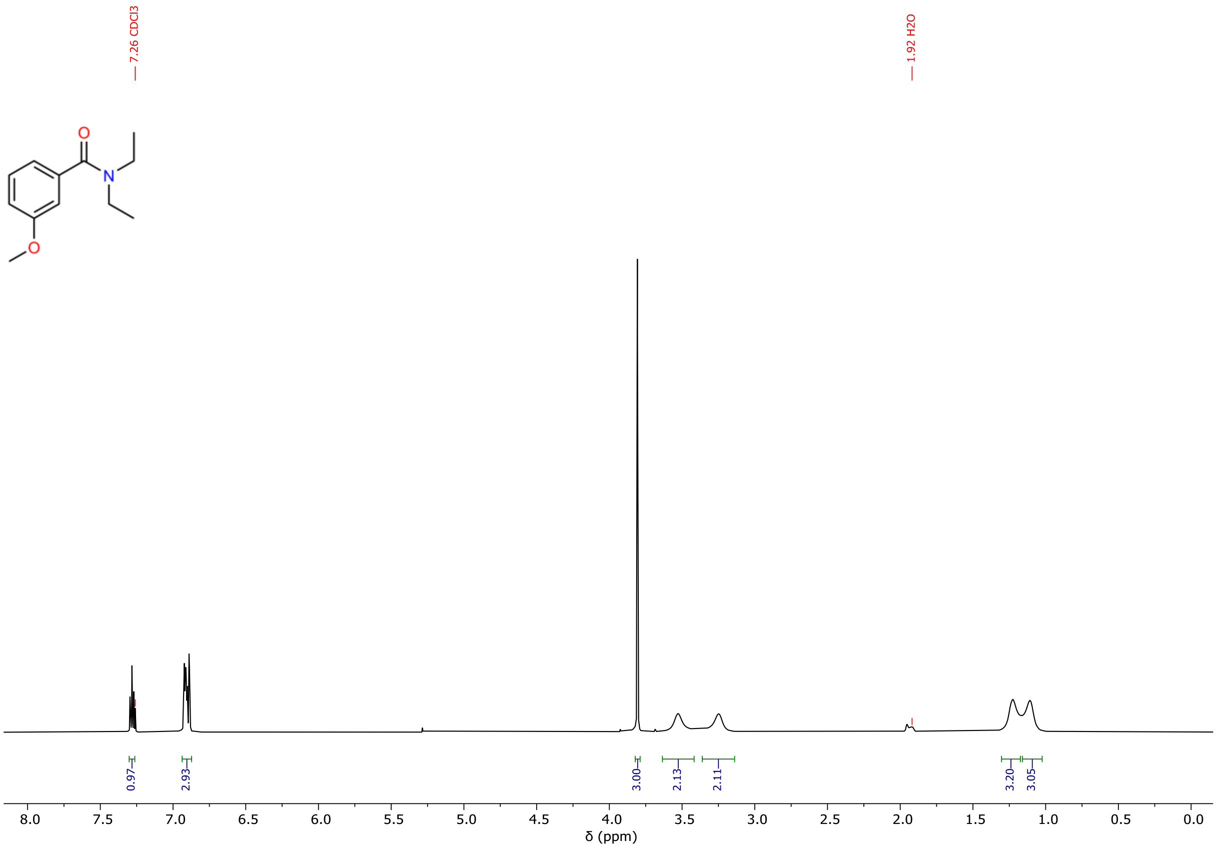

Supplementary Fig. 30 | ^{1}H NMR spectrum of amide **7a** (600 MHz, $CDCl_3$, 25 °C).

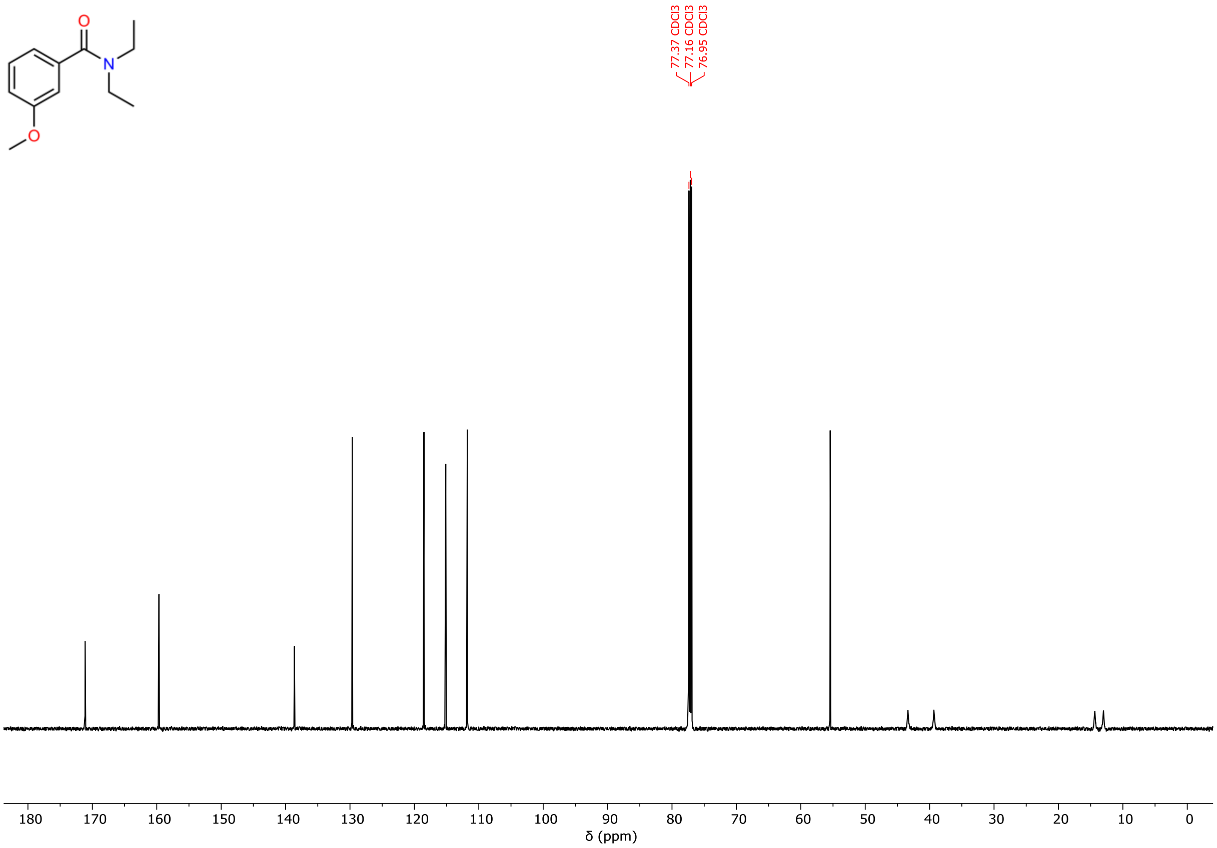

Supplementary Fig. 31 | ^{13}C NMR spectrum of amide **7a** (151 MHz, $CDCl_3$, 25 °C).

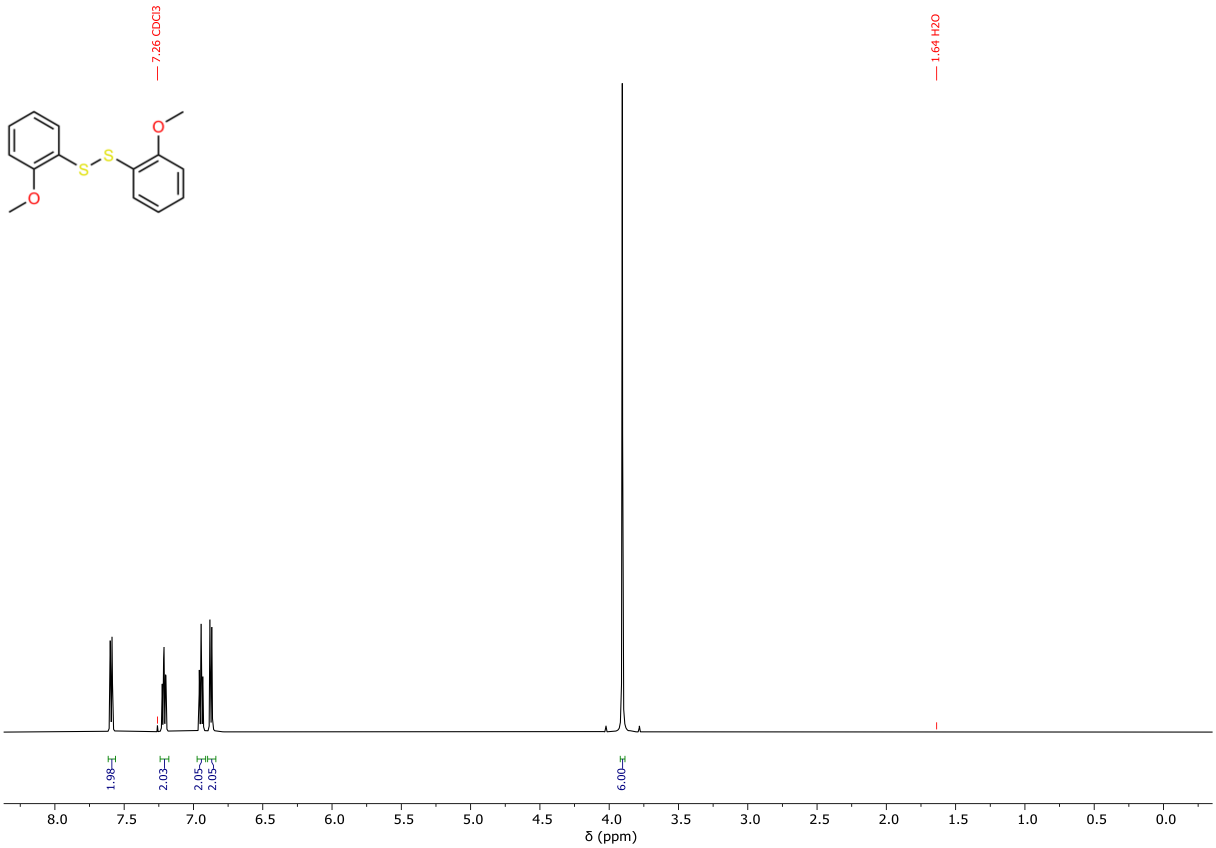

Supplementary Fig. 32 | ^{1}H NMR spectrum of disulfane **7b** (600 MHz, $CDCl_3$, 25 °C).

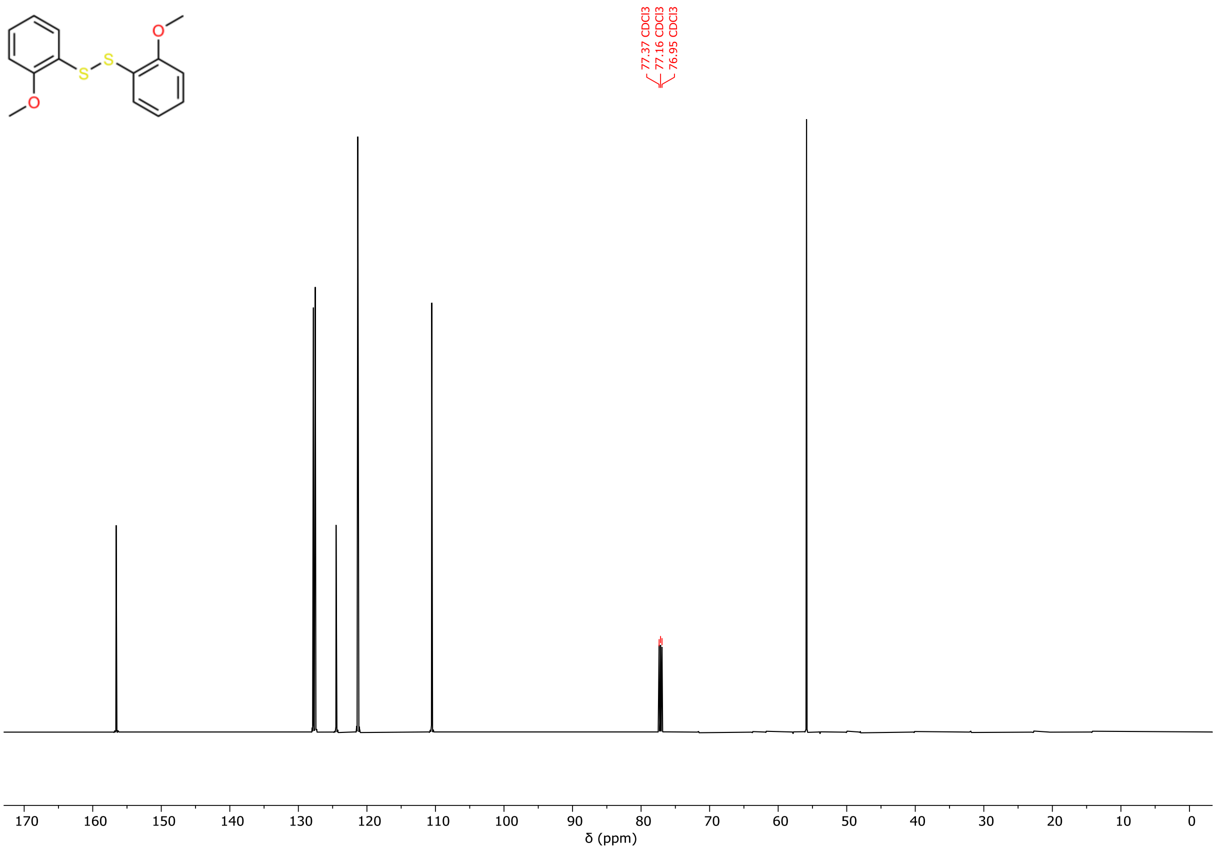

Supplementary Fig. 33 | ^{13}C NMR spectrum of disulfane **7b** (151 MHz, $CDCl_3$, 25 °C).

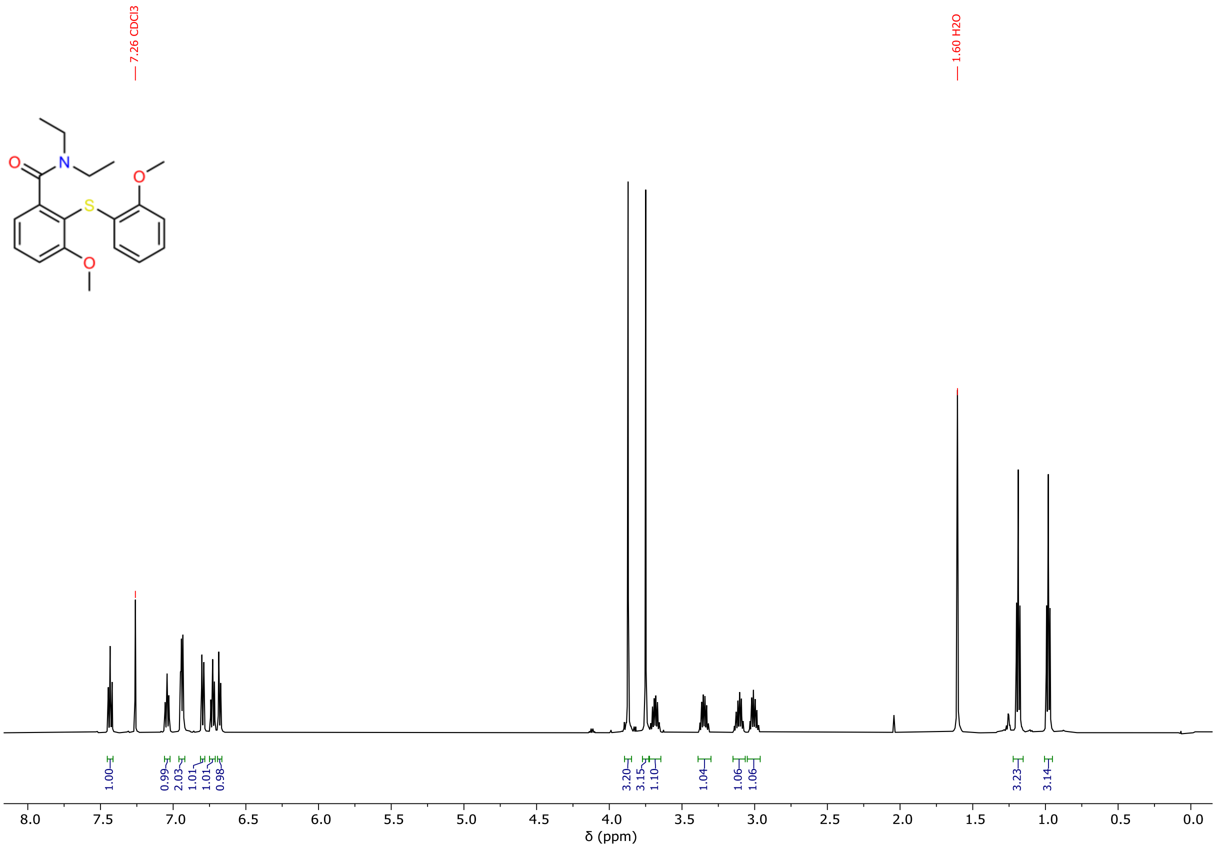

Supplementary Fig. 34 | ^{1}H NMR spectrum of amide **7c** (600 MHz, $CDCl_3$, 25 °C).

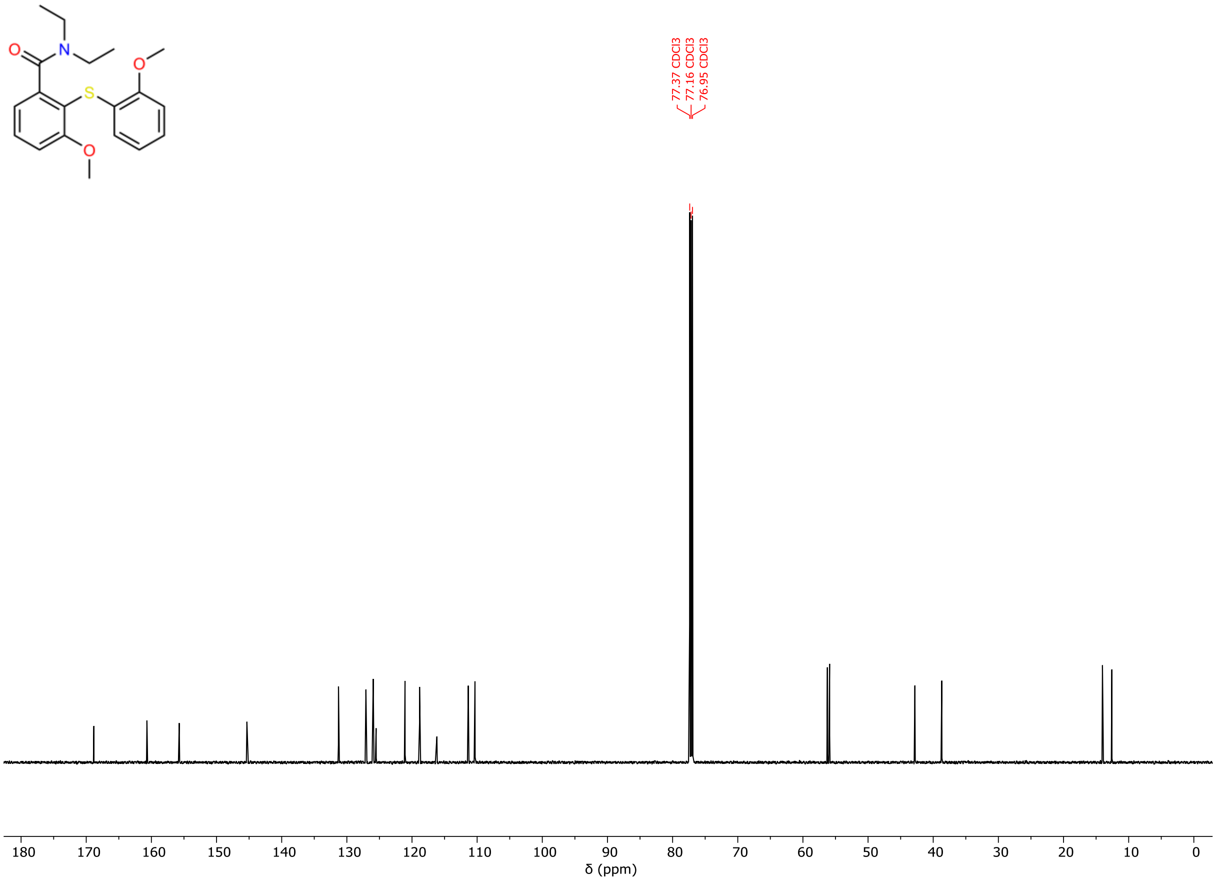

Supplementary Fig. 35 | ^{13}C NMR spectrum of amide **7c** (151 MHz, $CDCl_3$, 25 °C).

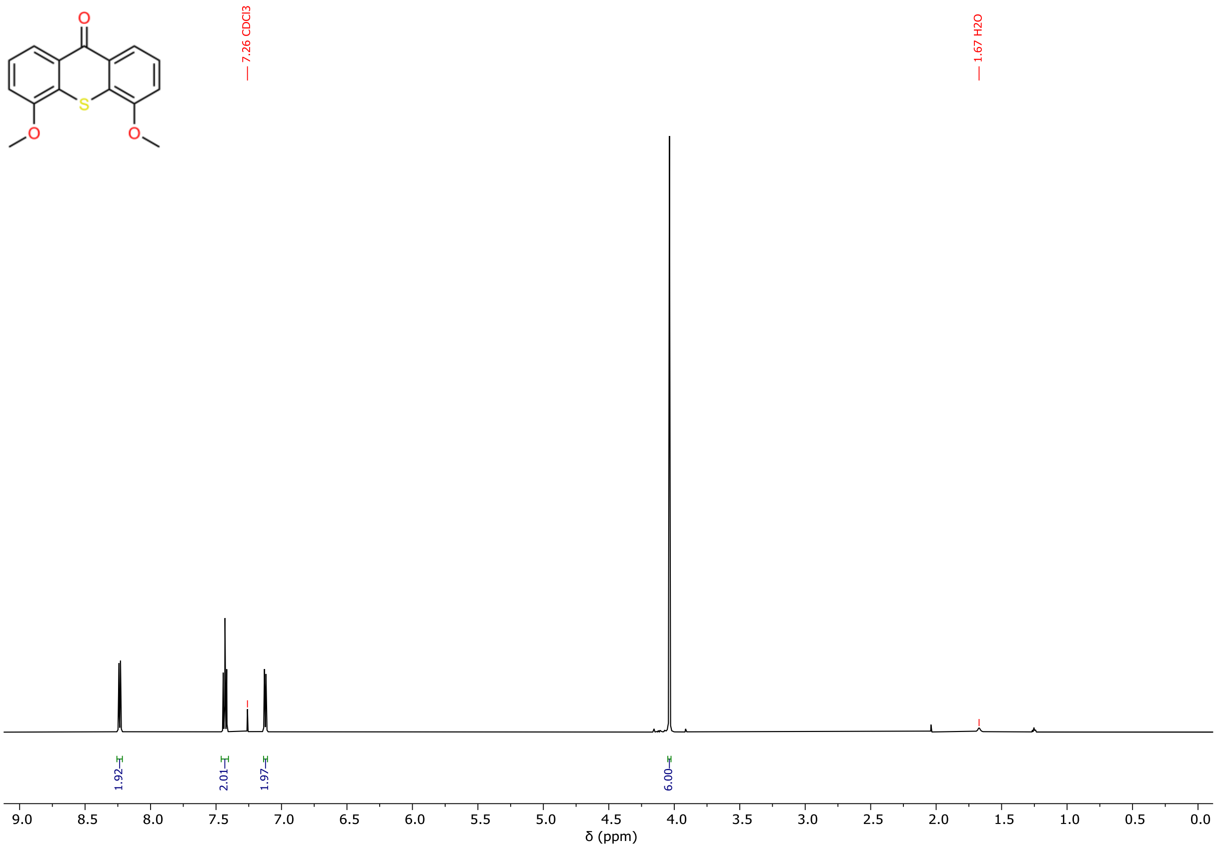

Supplementary Fig. 36 | ^{1}H NMR spectrum of thioxanthone **7d** (600 MHz, $CDCl_3$, 25 °C).

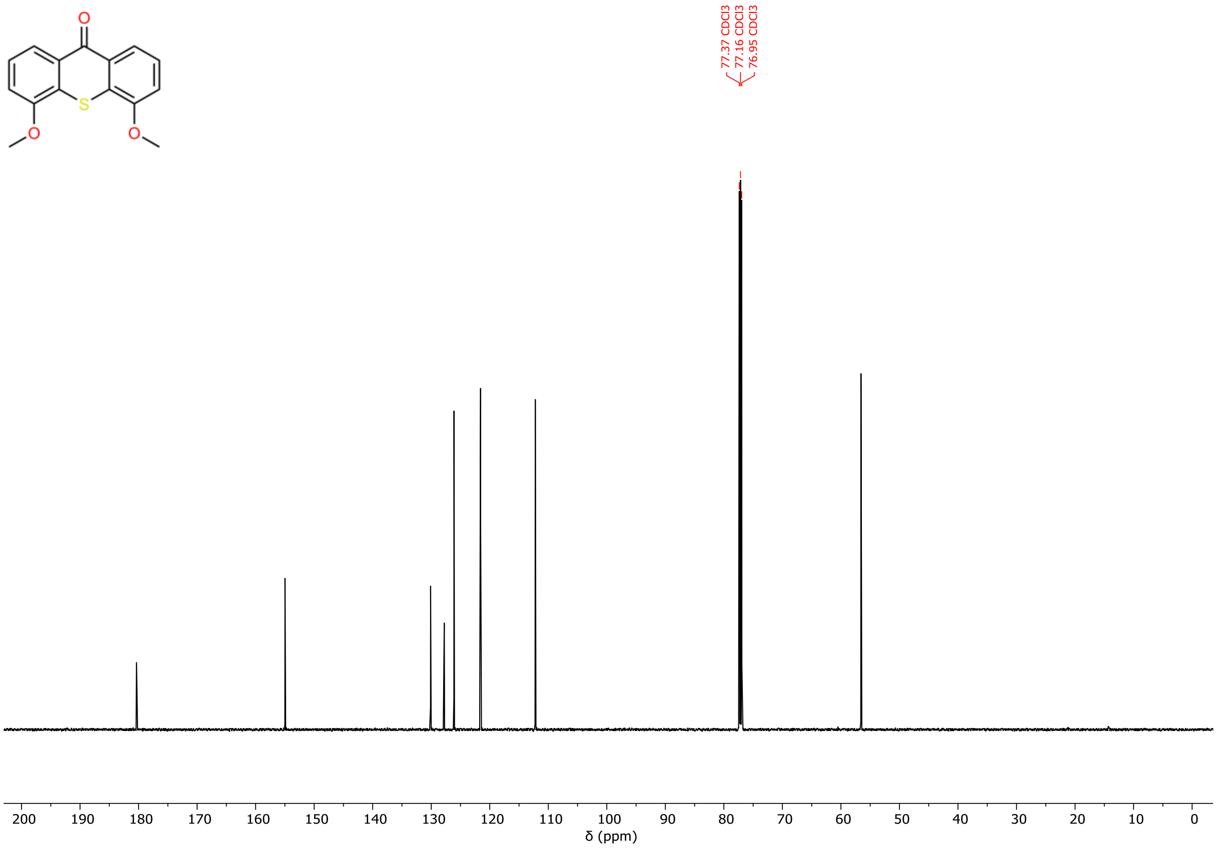

Supplementary Fig. 37 | ^{13}C NMR spectrum of thioxanthone **7d** (151 MHz, $CDCl_3$, 25 °C).

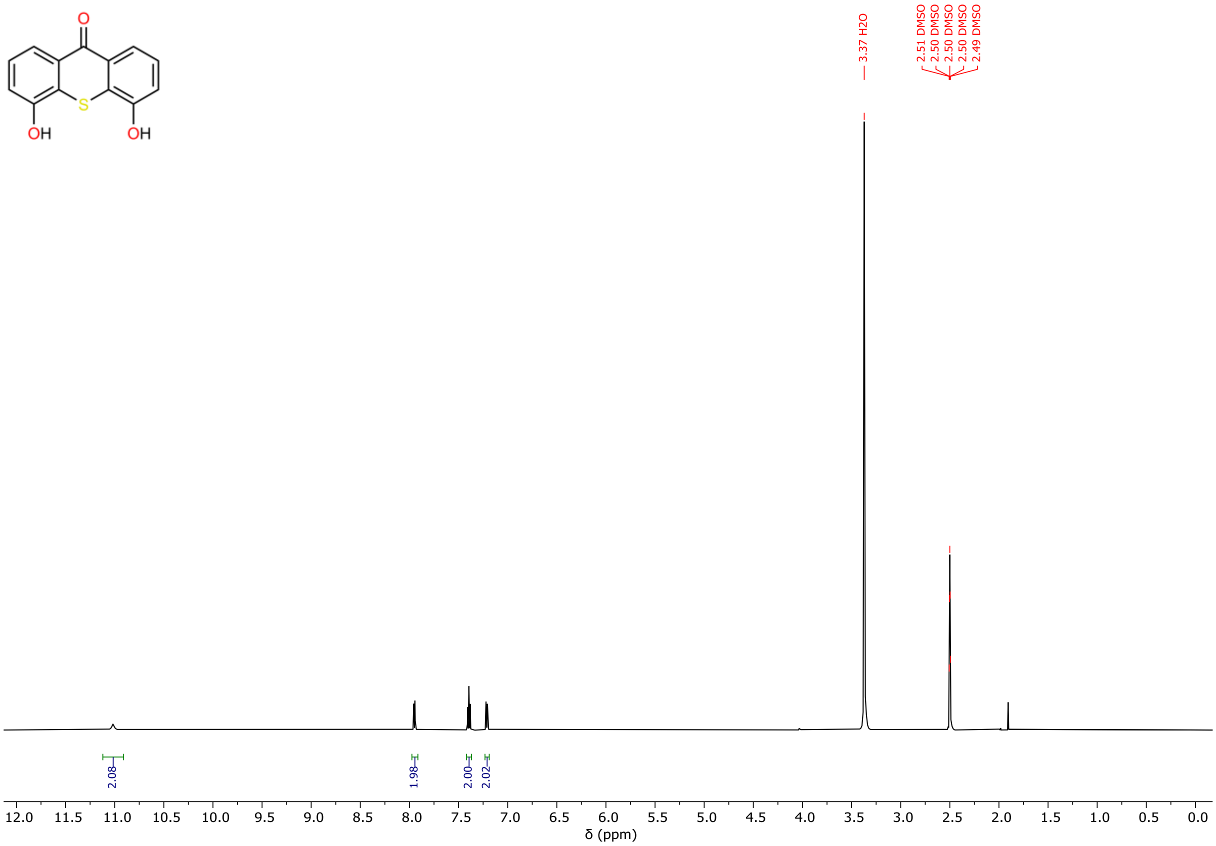

Supplementary Fig. 38 | ^{1}H NMR spectrum of phenol **7e** (600 MHz, DMSO-d_6, 25 °C).

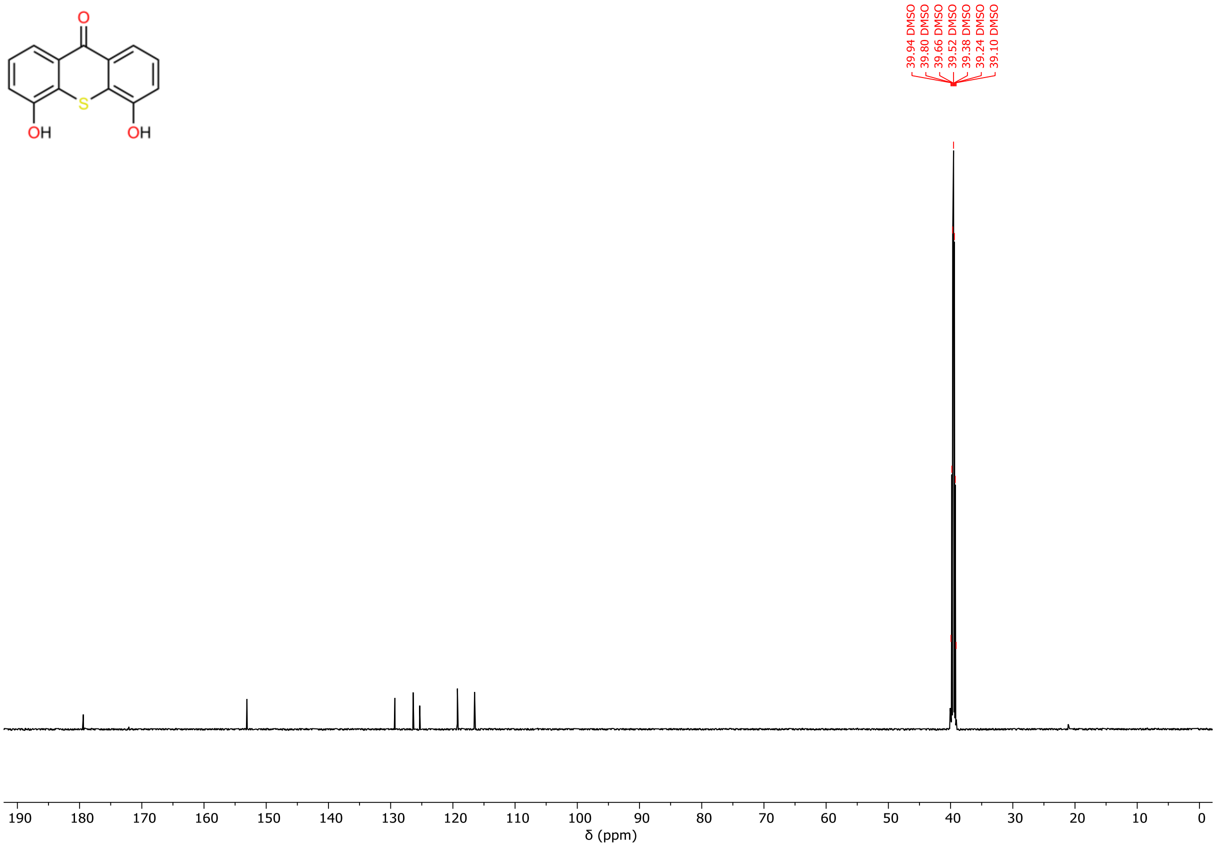

Supplementary Fig. 39 | ^{13}C NMR spectrum of phenol **7e** (151 MHz, DMSO-d_6, 25 °C).

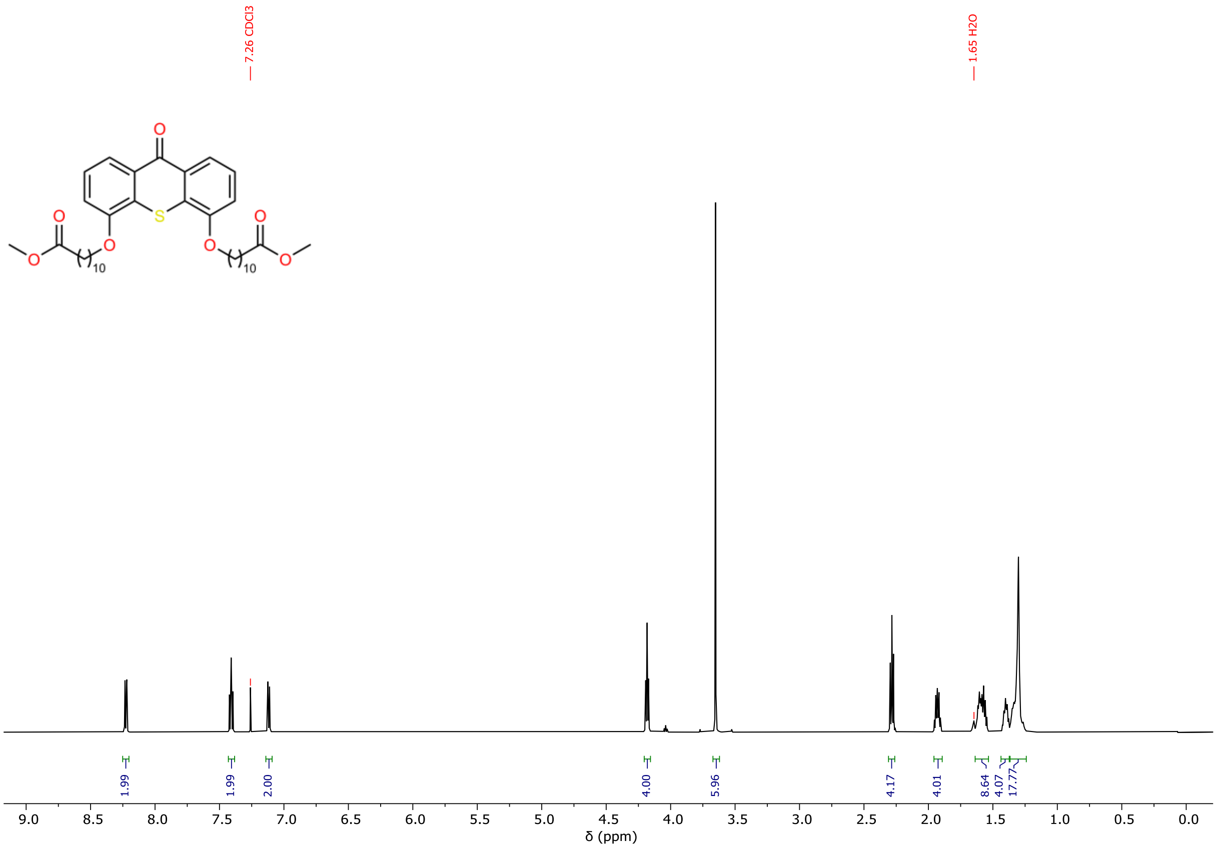

Supplementary Fig. 40 | ^{1}H NMR spectrum of thioxanthone **7** (600 MHz, $CDCl_3$, 25 °C).

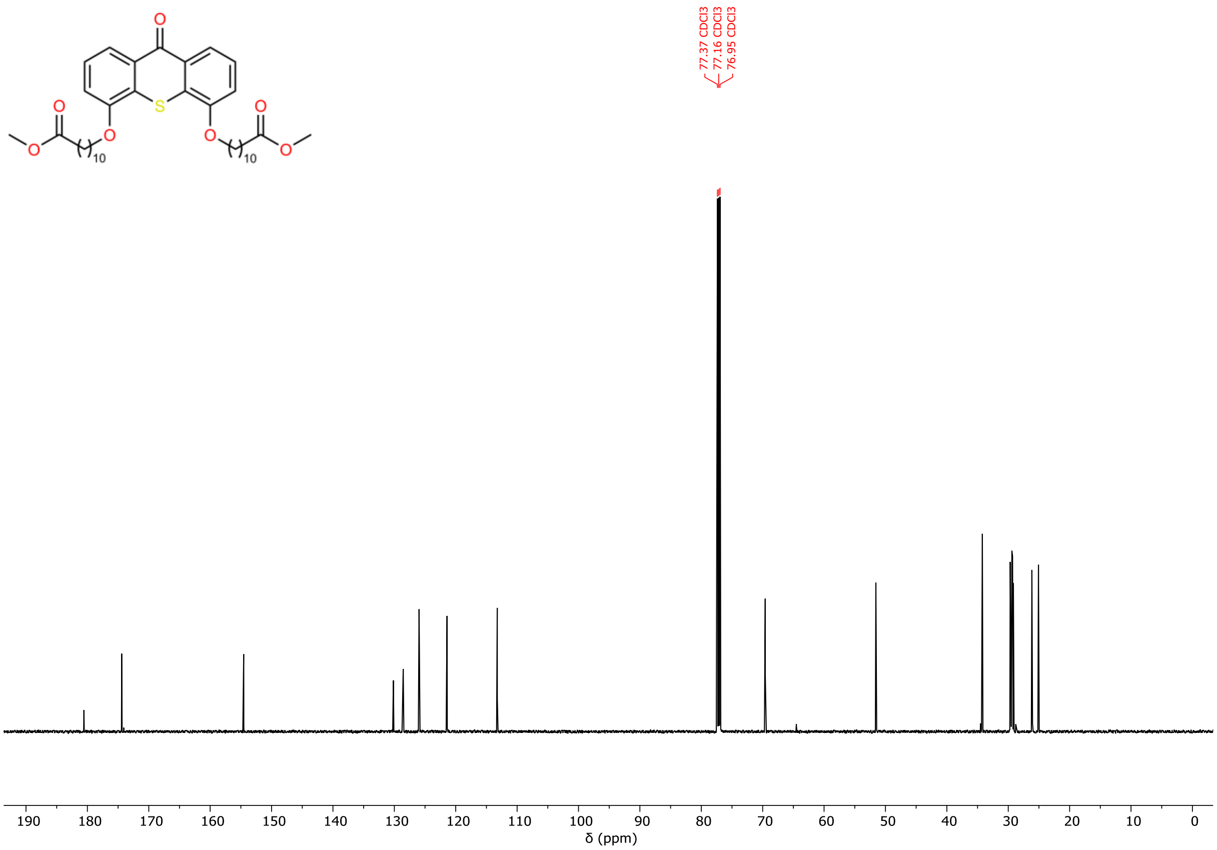

Supplementary Fig. 41 | ^{13}C NMR spectrum of thioxanthone **7** (151 MHz, $CDCl_3$, 25 °C).

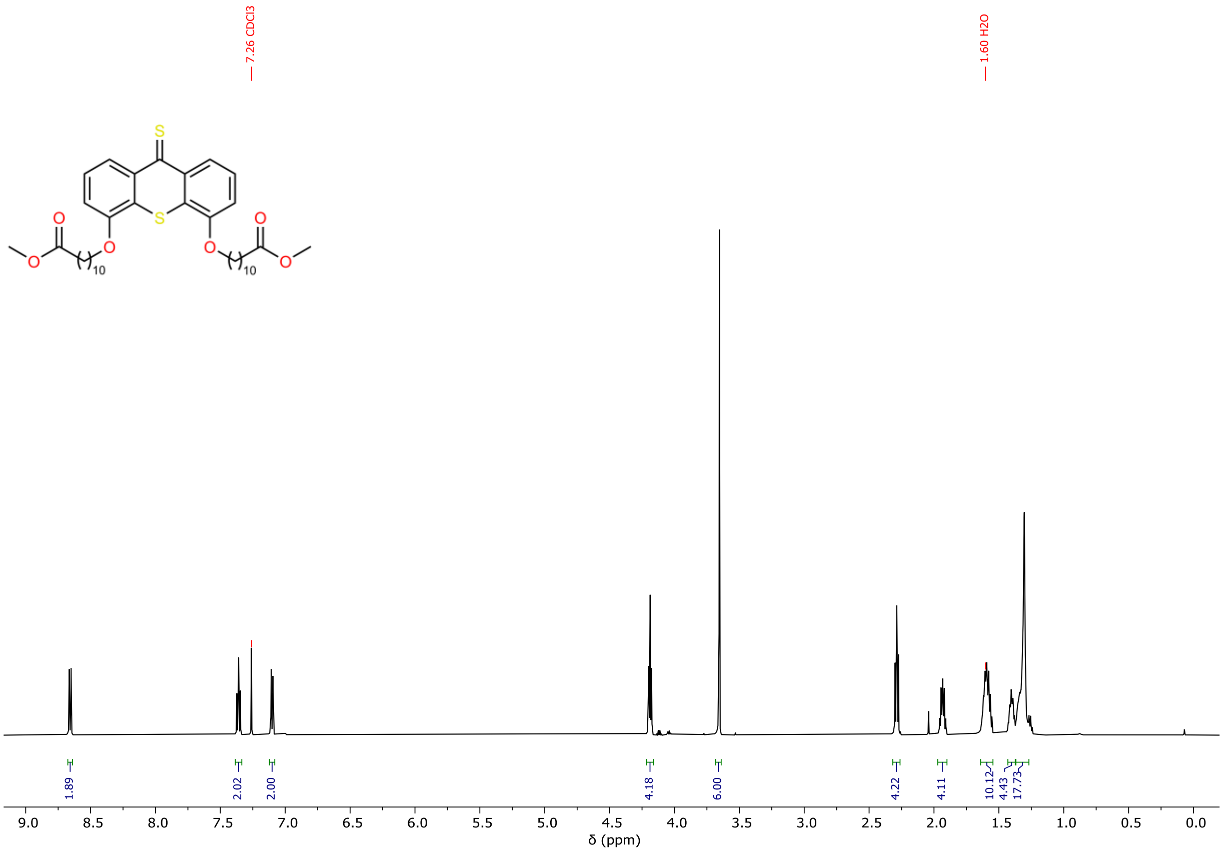

Supplementary Fig. 42 | ^{1}H NMR spectrum of thioketone **8** (600 MHz, $CDCl_3$, 25 °C).

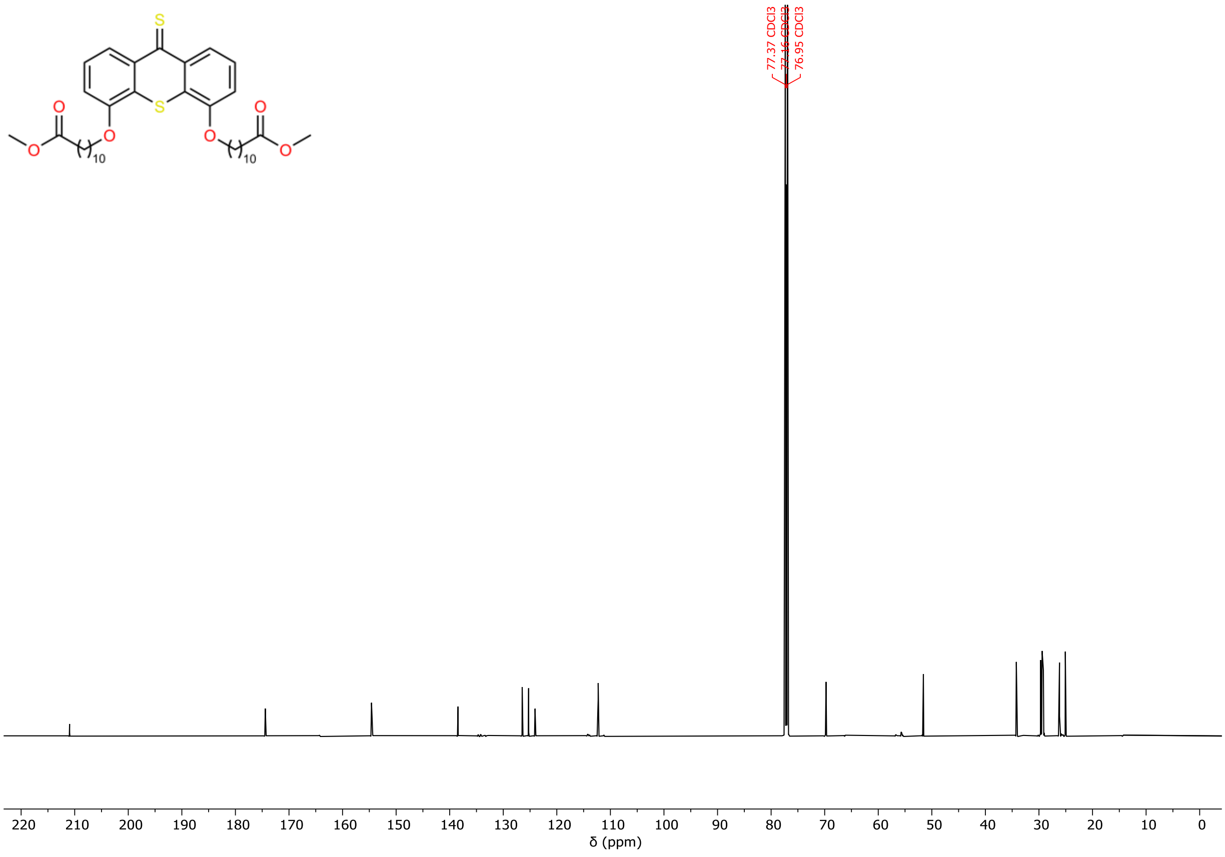

Supplementary Fig. 43 | ^{13}C NMR spectrum of thioketone **8** (151 MHz, $CDCl_3$, 25 °C).

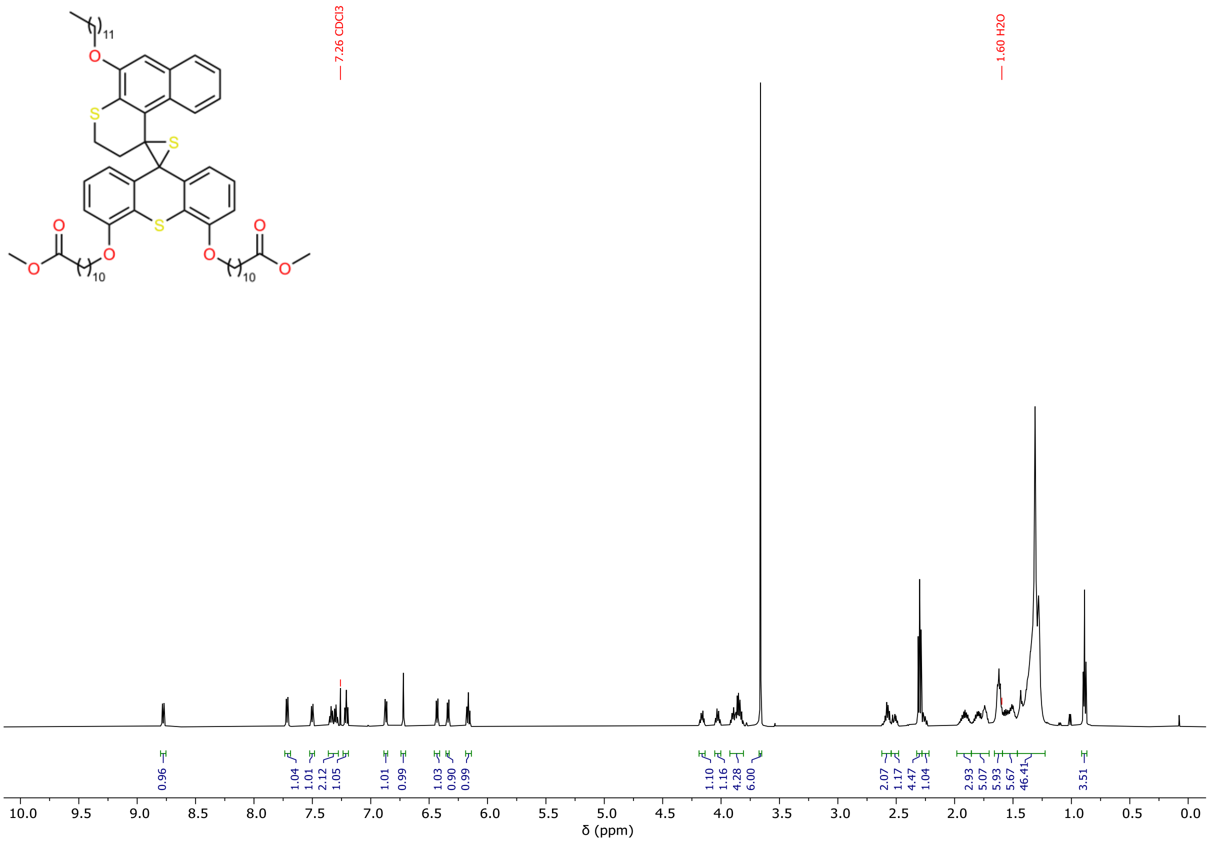

Supplementary Fig. 44 | ^{1}H NMR spectrum of episulfide **10** (600 MHz, $CDCl_3$, 25 °C).

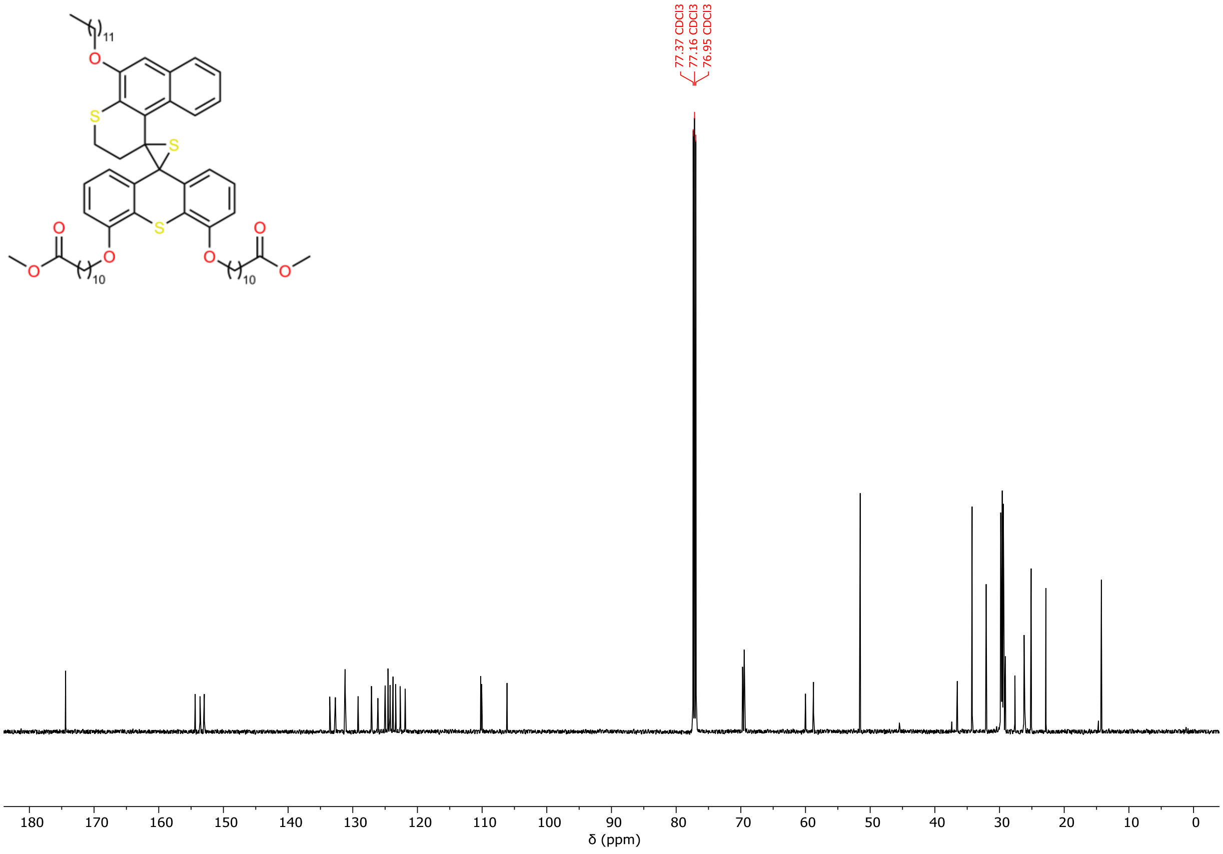

Supplementary Fig. 45 | ^{13}C NMR spectrum of episulfide **10** (151 MHz, $CDCl_3$, 25 °C).

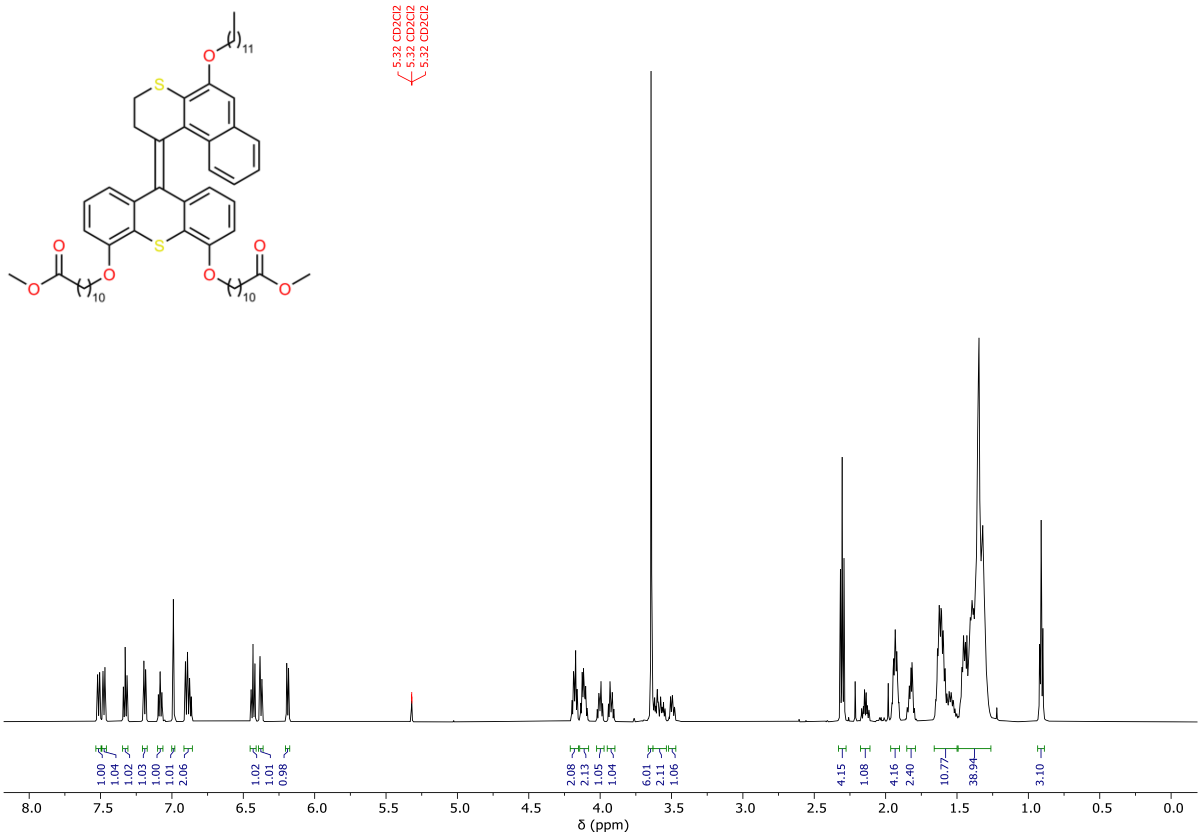

Supplementary Fig. 46 | ^{1}H NMR spectrum of overcrowded alkene **11** (600 MHz, CD_2Cl_2, 25 °C).

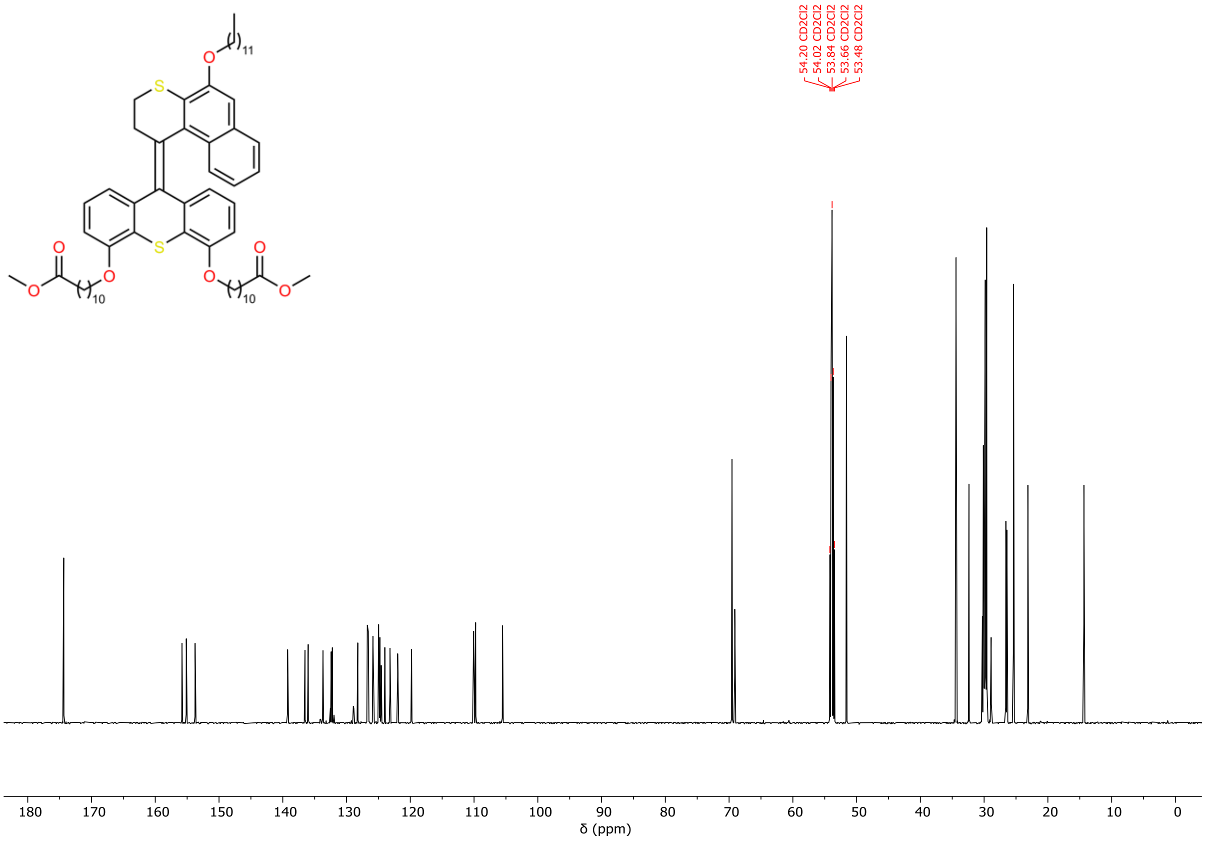

Supplementary Fig. 47 | ^{13}C NMR spectrum of overcrowded alkene **11** (151 MHz, CD_2Cl_2, 25 °C).

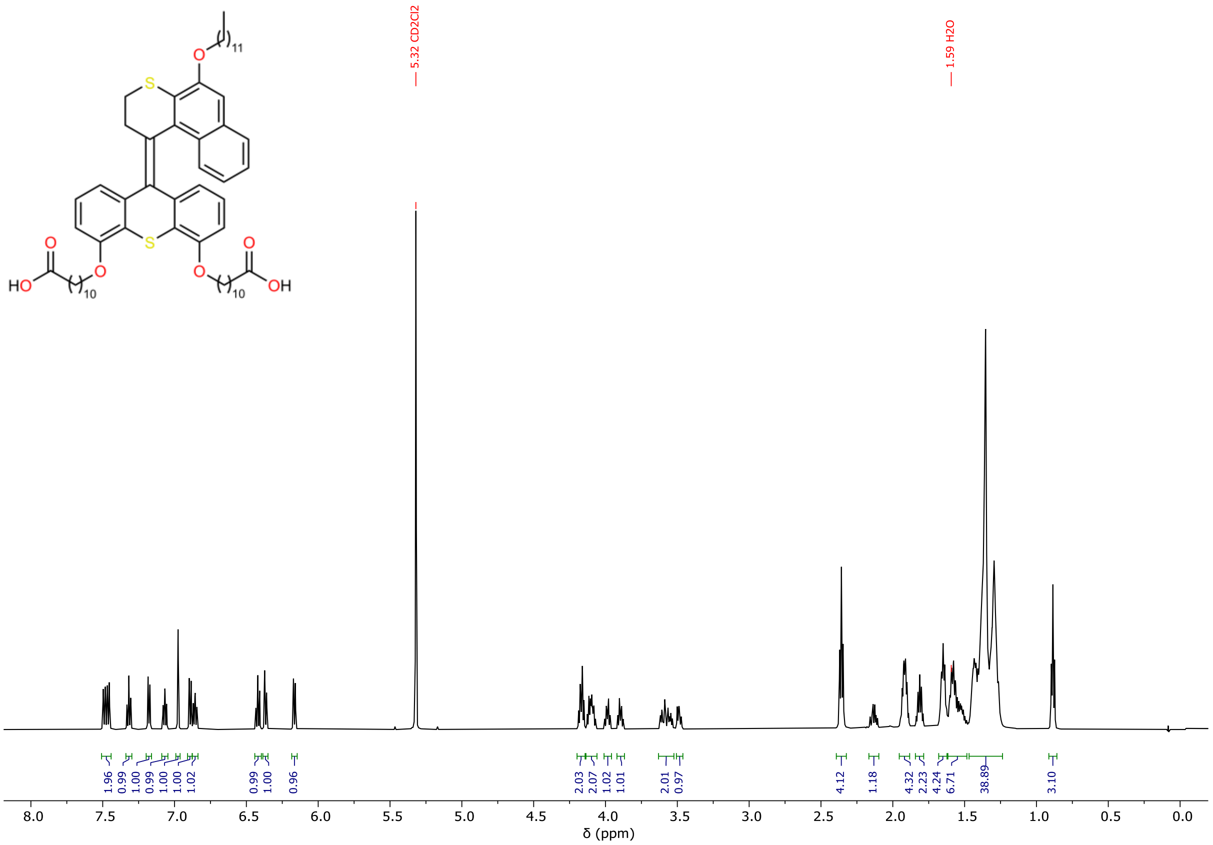

Supplementary Fig. 48 | ^{1}H NMR spectrum of overcrowded alkene **SA** (600 MHz, CD_2Cl_2, 25 °C).

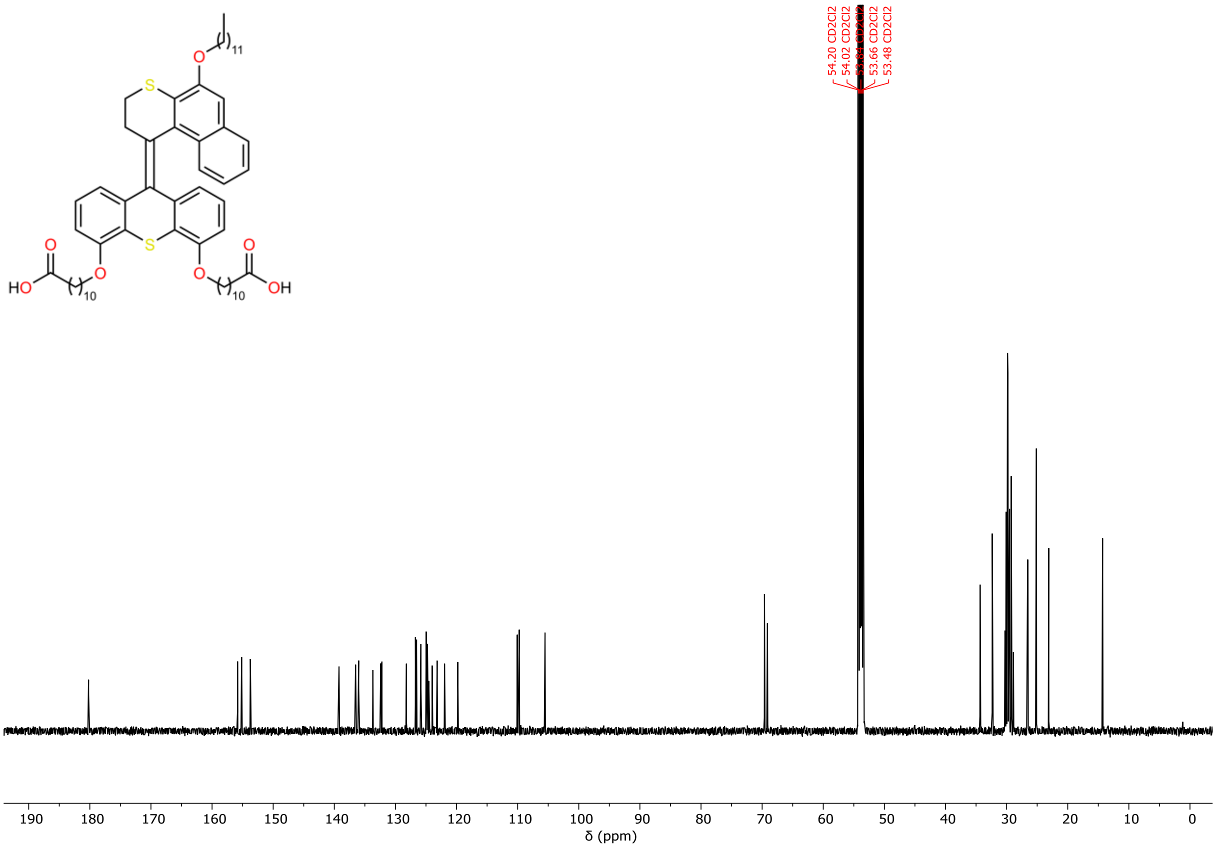

Supplementary Fig. 49 | ^{13}C NMR spectrum of overcrowded alkene **SA** (151 MHz, CD_2Cl_2, 25 °C).

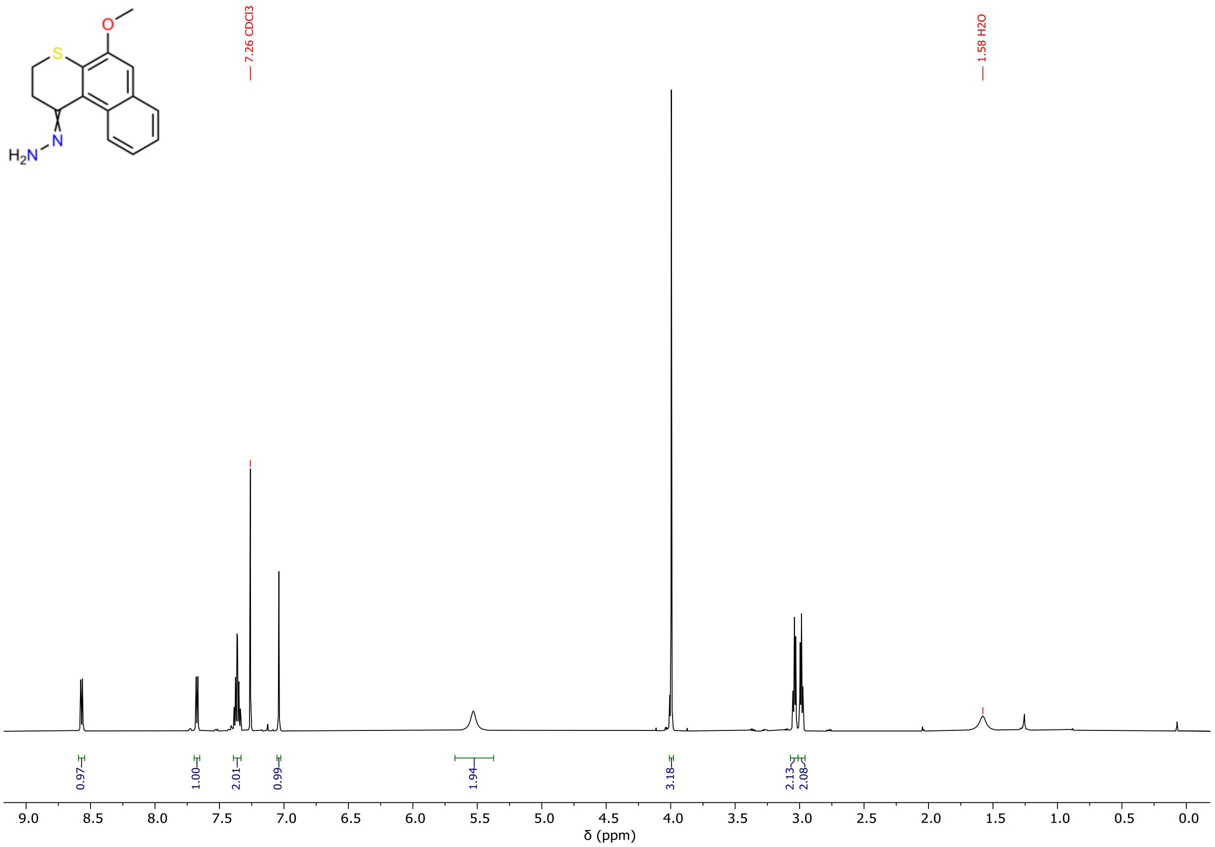

Supplementary Fig. 50 | ^{1}H NMR spectrum of hydrazone **12** (600 MHz, $CDCl_3$, 25 °C).

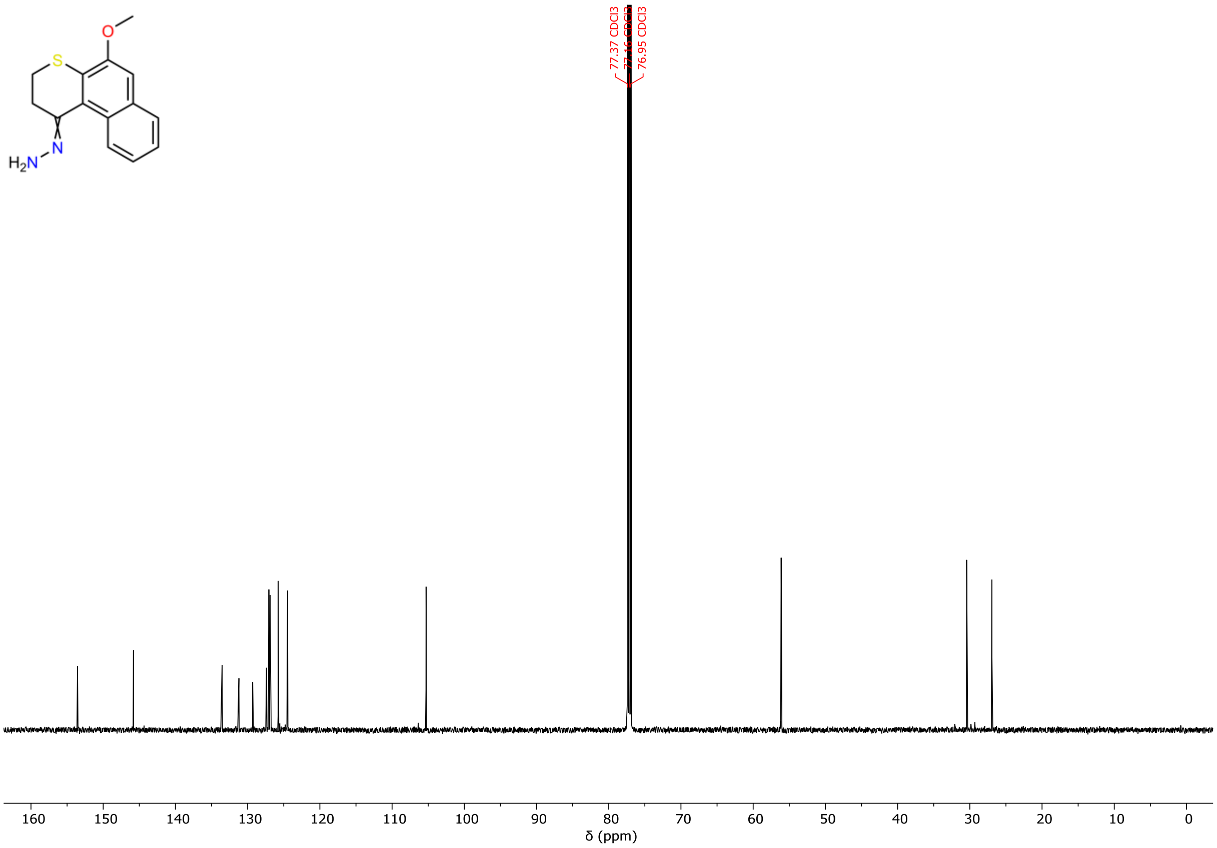

Supplementary Fig. 51 | ^{13}C NMR spectrum of hydrazone **12** (151 MHz, $CDCl_3$, 25 °C).

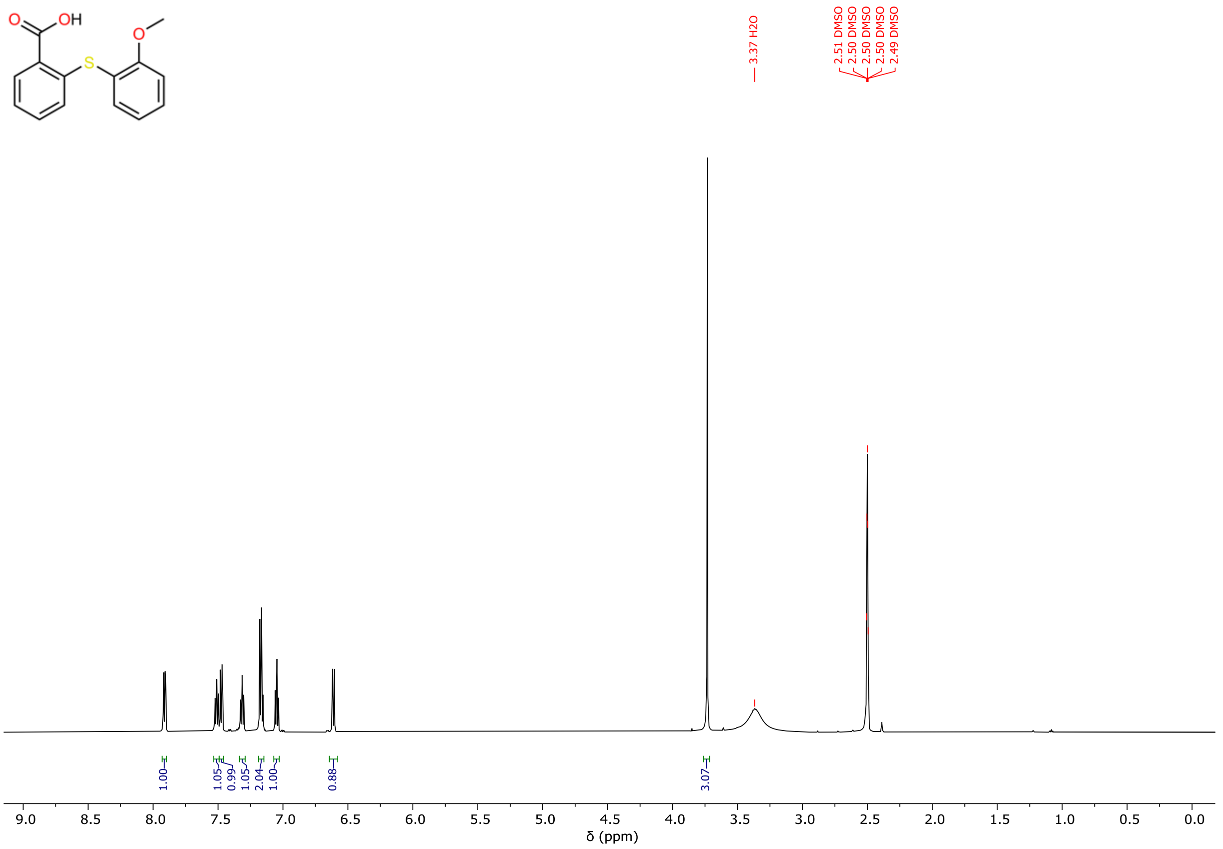

Supplementary Fig. 52 | ^{1}H NMR spectrum of benzoic acid **13** (600 MHz, DMSO-d_6, 25 °C).

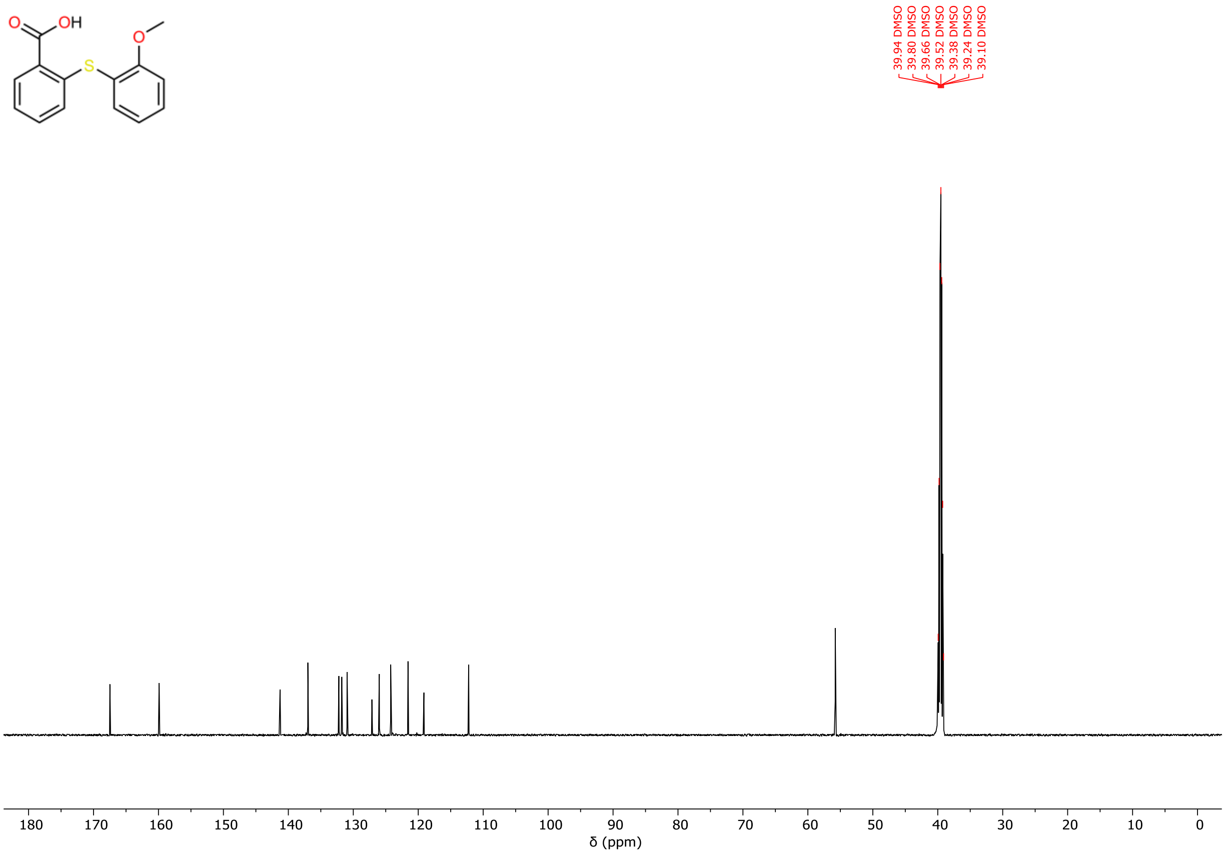

Supplementary Fig. 53 | ^{13}C NMR spectrum of benzoic acid **13** (151 MHz, DMSO-d_6, 25 °C).

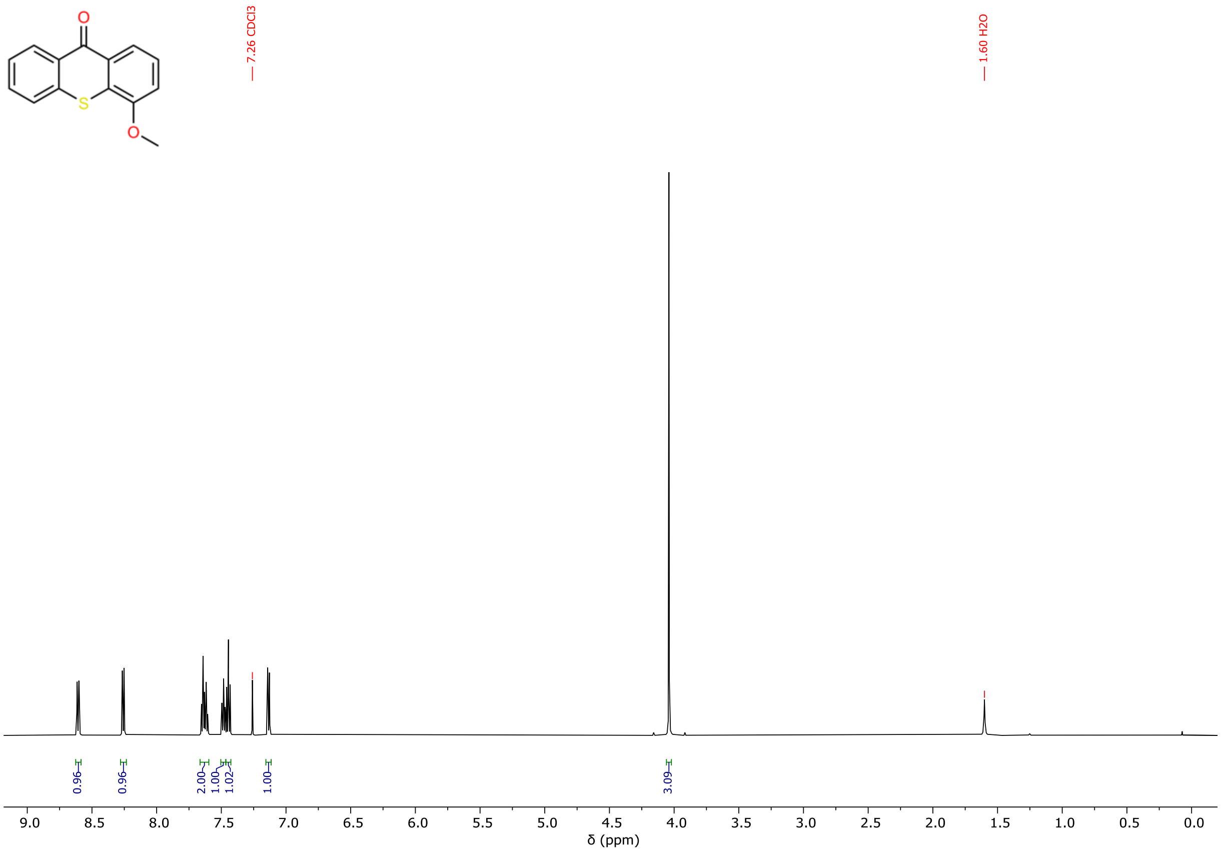

Supplementary Fig. 54 | ^{1}H NMR spectrum of thioxanthone **14** (600 MHz, $CDCl_3$, 25 °C).

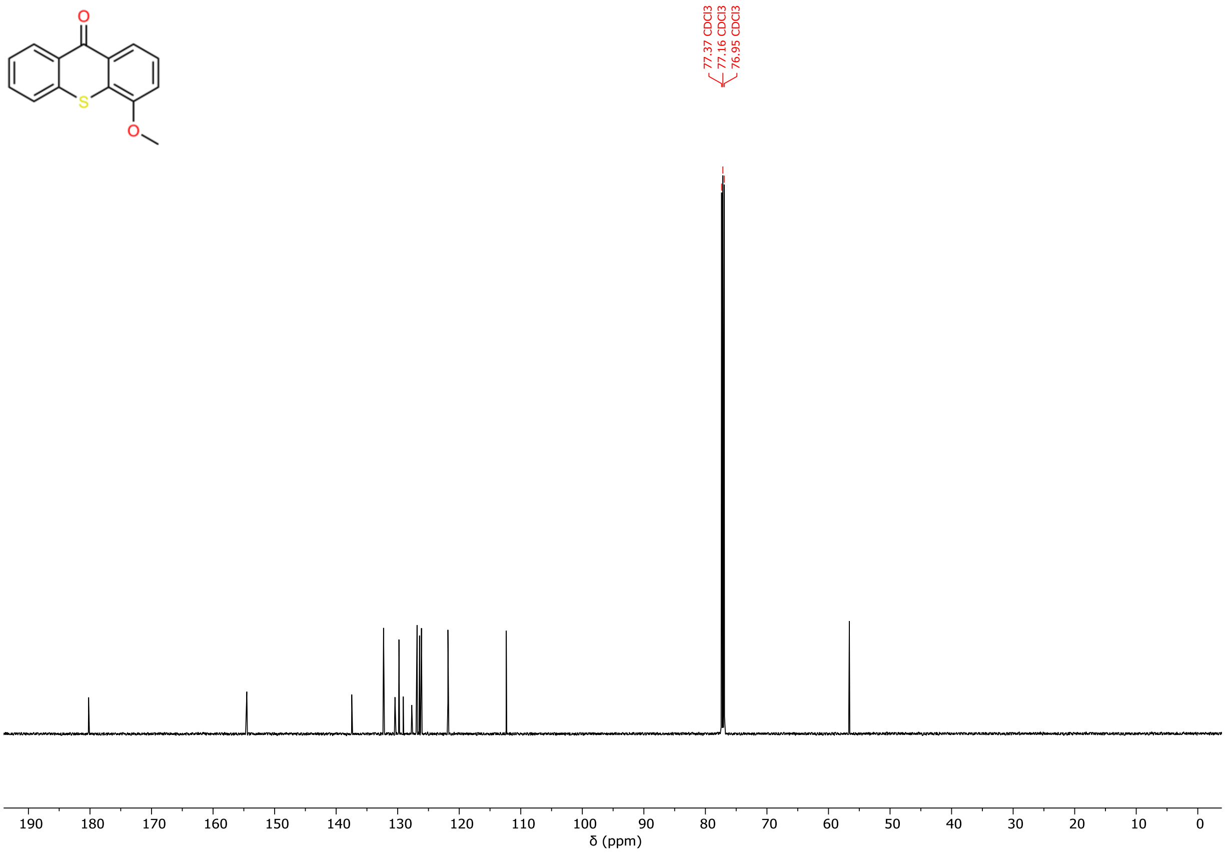

Supplementary Fig. 55 | ^{13}C NMR spectrum of thioxanthone **14** (151 MHz, $CDCl_3$, 25 °C).

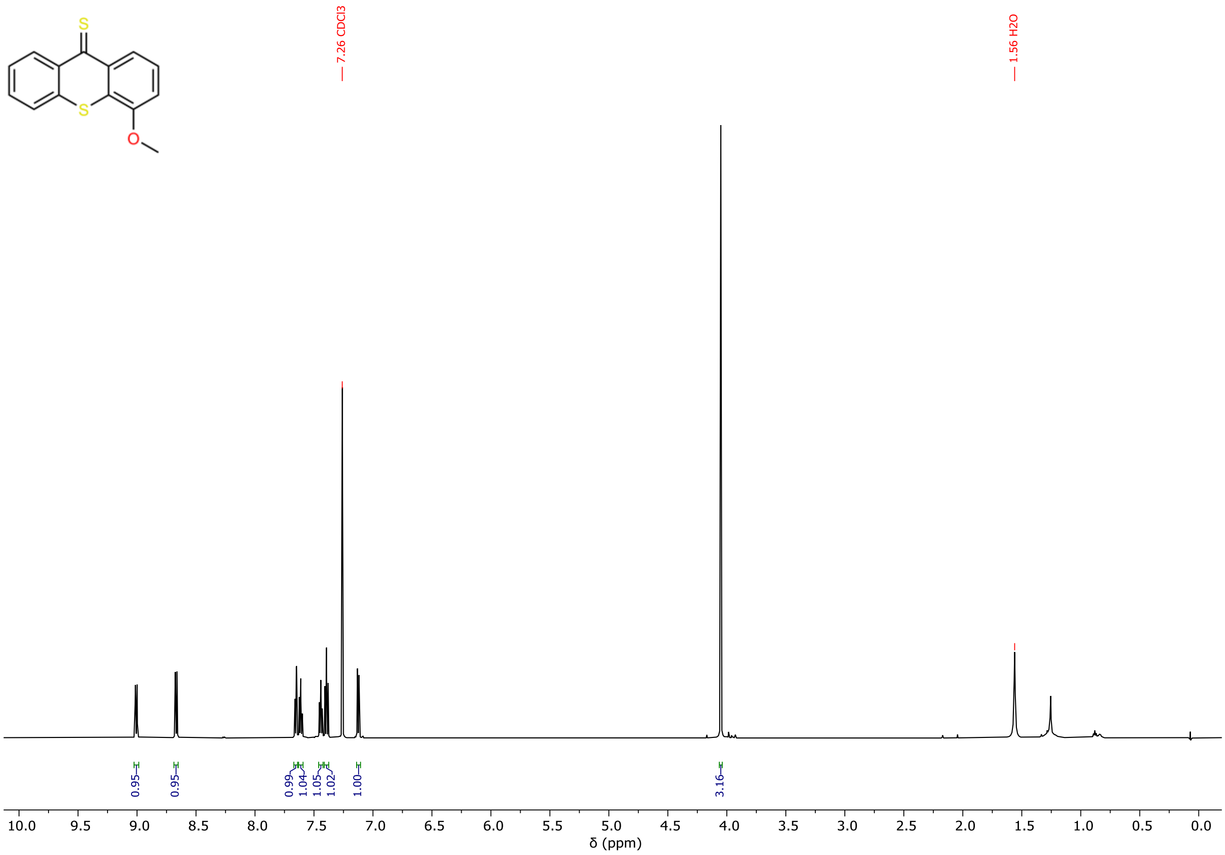

Supplementary Fig. 56 | ^{1}H NMR spectrum of thioketone **15** (600 MHz, $CDCl_3$, 25 °C).

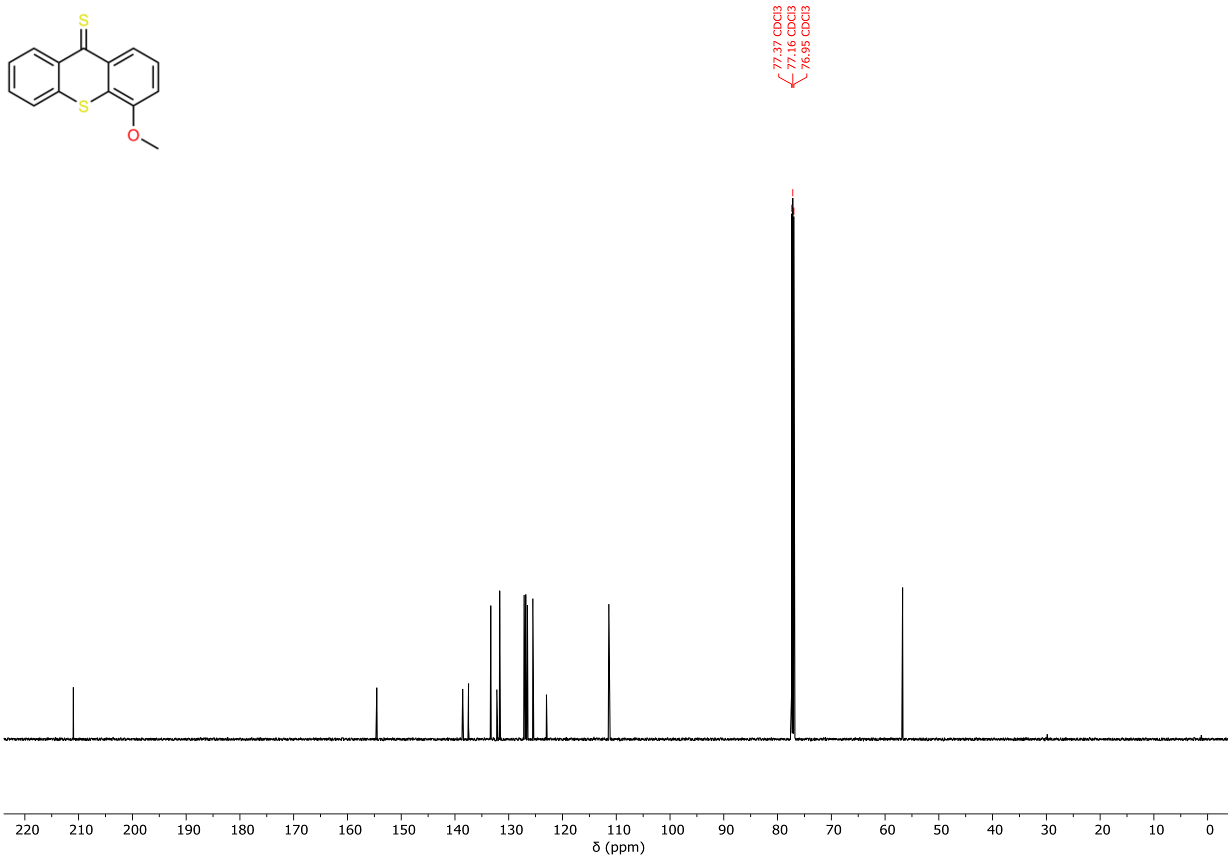

Supplementary Fig. 57 | ^{13}C NMR spectrum of thioketone **15** (151 MHz, $CDCl_3$, 25 °C).

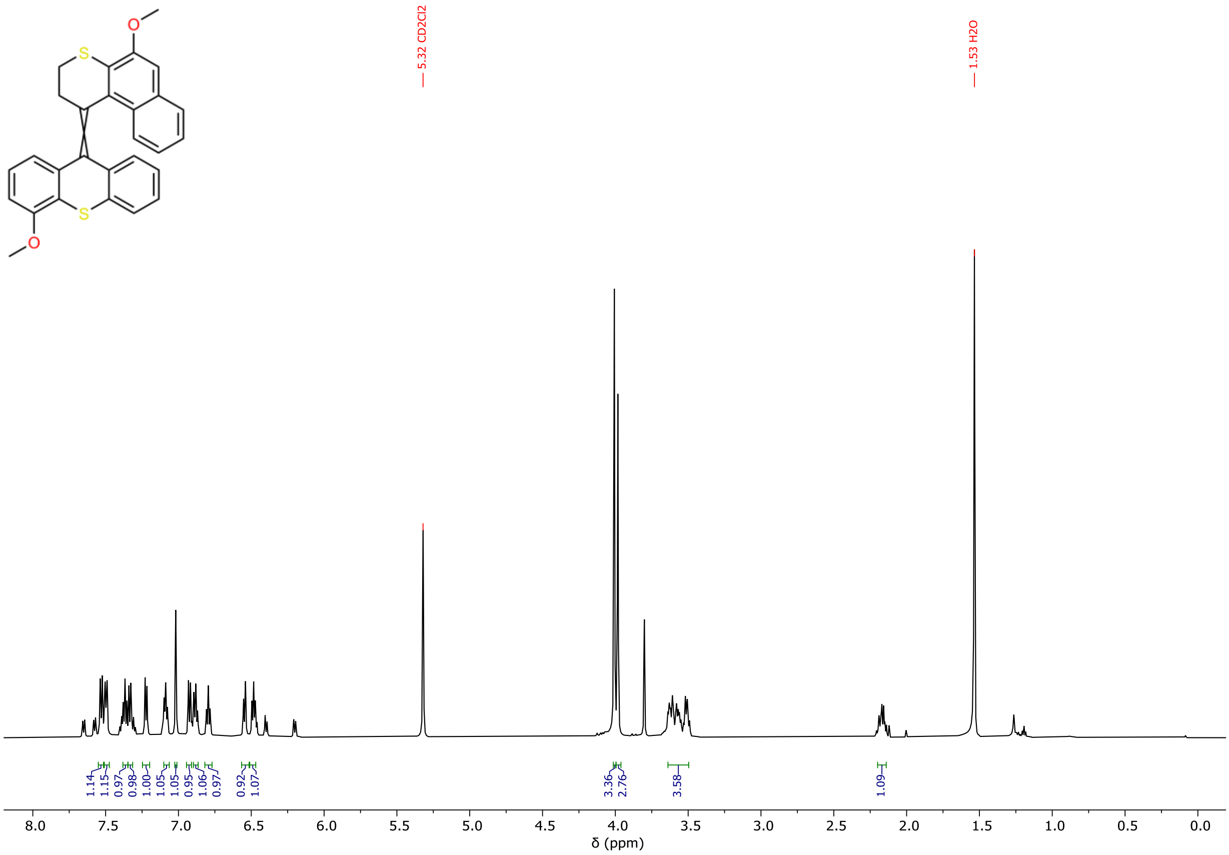

Supplementary Fig. 58 | ^{1}H NMR spectrum of overcrowded alkene **BMS** (600 MHz, CD_2Cl_2, 25 °C).

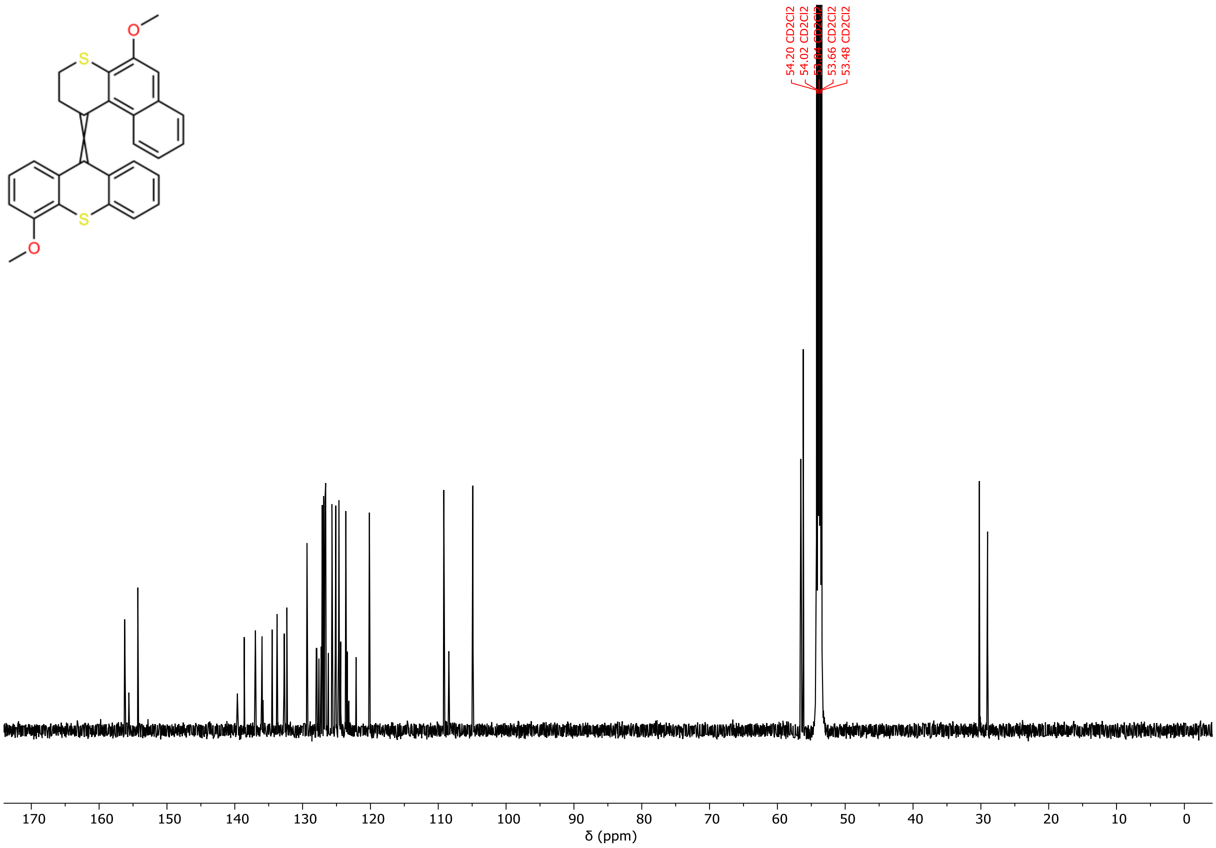

Supplementary Fig. 59 | ^{13}C NMR spectrum of overcrowded alkene **BMS** (151 MHz, CD_2Cl_2, 25 °C).

3. Supplementary References

1. van Dijken, D. J., Chen, J., Stuart, M. C. A., Hou, L. & Feringa, B. L. Amphiphilic molecular motors for responsive aggregation in water. *J. Am. Chem. Soc.* **138**, 660–669 (2016).

2. Chen, J. *et al.* Artificial muscle-like function from hierarchical supramolecular assembly of photoresponsive molecular motors. *Nat. Chem.* **10**, 132–138 (2018).

3. Wu, X. D., Liu, D. Z., Li, A. J. & Zhou, X. Q. N-(2-(2-Methoxyphenylthio)benzyl)-2-aryloxyethylamines: synthesis and evaluation for dual 5-HT1A/SSRI activities. *Chin. Chem. Lett.* **19**, 295–298 (2008).
