# Supplementary material for: Photoactuating artificial muscle from supramolecular assembly of an overcrowded alkene-derived molecular switch
Source: Nat Commun. 2025 Apr 24;16:3897. doi: 10.1038/s41467-025-58468-0 (PMC12022091; doi:10.1038/s41467-025-58468-0)
Supplement: Supplementary file 2 — Description of Additional Supplementary Files [file 41467_2025_58468_MOESM2_ESM.pdf]

# Description of Additional Supplementary Files

**File name: Supplementary Movie 1**
**Description:** Photoactuation of **SA** artificial muscle (60 mM) in $MgCl_2$ aqueous solution (150 mM).

**File name: Supplementary Movie 2**
**Description:** Photoactuation of **SA** artificial muscle (60 mM) in $CaCl_2$ aqueous solution (150 mM).

**File name: Supplementary Movie 3**
**Description:** Photoactuation of **SA** artificial muscle (60 mM) in $SrCl_2$ aqueous solution (150 mM).

**File name: Supplementary Movie 4**
**Description:** Photoactuation of **SA** artificial muscle (60 mM) in $BaCl_2$ aqueous solution (150 mM).
